# Pharmacophore Modeling of Janus Kinase Inhibitors: Tools for Drug Discovery and Exposition Prediction

**DOI:** 10.3390/molecules30102183

**Published:** 2025-05-16

**Authors:** Florian Fischer, Veronika Temml, Daniela Schuster

**Affiliations:** Department of Pharmaceutical and Medicinal Chemistry, Institute of Pharmacy and Research and Innovation Center for Regenerative Medicine and Novel Therapies, Paracelsus Medical University, 5020 Salzburg, Austria; florian.fischer@pmu.ac.at (F.F.); veronika.temml@pmu.ac.at (V.T.)

**Keywords:** pesticides, JAK1, JAK2, JAK3, TYK2, immunosuppression, pharmacophore modeling

## Abstract

Pesticides are essential in agriculture for protecting crops and boosting productivity, but their widespread use may pose significant health risks. Farmworkers face direct exposure through skin contact and inhalation, which may lead to hormonal imbalances, neurological disorders, and elevated cancer risks. Moreover, pesticide residues in food and water may affect surrounding communities. One of the lesser investigated issues is immunotoxicity, mostly because the chronic effects of compound exposure are very complex to study. As a case study, this work utilized pharmacophore modeling and virtual screening to identify pesticides that may inhibit Janus kinases (JAK1, JAK2, JAK3) and tyrosine kinase 2 (TYK2), which are pivotal in immune response regulation, and are associated with cancer development and increased infection susceptibility. We identified 64 potential pesticide candidates, 22 of which have previously been detected in the human body, as confirmed by the Human Metabolome Database. These results underscore the critical need for further research into potential immunotoxic and chronic impacts of the respective pesticides on human health.

## 1. Introduction

According to the European Commission (EC) (https://food.ec.europa.eu/plants/pesticides_en, accessed on 14 April 2025), pesticides are used to prevent, destroy, or control harmful organisms (‘pests’) or diseases, and to protect plants or plant products during production, storage, and transport. This category includes various agents, such as herbicides, fungicides, insecticides, and biocides. In daily agriculture, pesticides play a vital role in safeguarding crops from insects, weeds, and other detrimental organisms, which ultimately profoundly enhances agricultural yields and food security. However, the extensive application of these agrochemicals may have notable consequences, particularly for farmworkers, who are the most exposed group [1,2,3]. This exposure can occur through various pathways, with dermal contact being a prevalent route. When substances touch the skin, they can be absorbed into the bloodstream, potentially causing localized or systemic effects. Inhalation of pesticide vapors or aerosols represents another significant exposure route, which can lead to respiratory issues and further systemic absorption [1,4,5].

Moreover, the broad and often uncontrolled use of biocides can lead to significant bioaccumulation and persistent residues in key environmental matrices, including food, feed, animal-derived products, soil, and water [6,7]. These chemicals can persist in crops and animal products, entering the food chain and posing a potential public health risk. As a result, even urban consumers who are not directly involved in agriculture can be exposed to pesticides through contaminated food and water. Over time, this indirect exposure can accumulate and contribute to various health problems, including endocrine disruptions, neurological disorders, and an elevated risk of cancers, such as prostate, lung, liver, breast, and colon cancer, as well as non-Hodgkin lymphoma and leukemia [5,8,9,10,11,12]. Experimental studies have also reported that exposure to pesticides can exert damaging effects on the immune system [13,14].

Our immune system is a highly sophisticated network that coordinates various pathways and specialized cells to defend the organism against pathogens and cancerous cells. Cytokines, produced by immune cells, are crucial for regulating immune functions, inflammation, and hematopoiesis. They exert their effects by binding to specific receptors, which in turn activate JAKs. These intracellular enzymes consist of four subtypes: Janus kinase 1 (JAK1), Janus kinase 2 (JAK2), Janus kinase 3 (JAK3), and tyrosine kinase 2 (TYK2). Each of these kinases contributes to the complex signaling pathways that manage immune responses and other vital biological processes [15,16,17].

Cytokines bind to the extracellular domains of their receptors, triggering conformational changes that activate associated JAK proteins. These JAKs undergo mutual transphosphorylation, enhancing their catalytic function. Subsequently, activated JAKs phosphorylate specific tyrosine residues on the receptor, creating binding sites for signal transducer and activator of transcription proteins (STATs). The STAT family involves seven subtypes: STAT1, STAT2, STAT3, STAT4, STAT5A, STAT5B, and STAT6, each with distinct roles in cellular processes, such as immune regulation, growth, and differentiation. Once phosphorylated, STAT proteins form dimers, translocate to the nucleus, and bind to gene promoter regions, initiating transcriptional changes in target cells [18].

By intervening in this system, for example with approved JAK inhibitors (jakibs) such as tofacitinib, baricitinib, and filgotinib, a state of immunosuppression can be induced. This mechanism is therapeutically exploited in treating conditions like rheumatoid arthritis and ulcerative colitis, where effectively controlling inflammation can lead to significant improvements in patient health and quality of life [19].

However, interfering with this signaling cascade can also lead to various health risks. The inhibition of JAKs may disrupt the regulation of blood cell production and immune response, resulting in complications such as increased susceptibility to infections, thrombosis, and potentially the development of malignancies [20].

This prospective computational study was based on the hypothesis that some pesticides, though designed for agricultural use, may interfere with the JAK signaling pathways akin to jakibs. In general, the health effects of pesticide exposure are difficult to assess, mainly due to the lack of long-term exposure data. Addressing this gap is crucial, since it could provide valuable insights into the broader activity spectra and to the risks of pesticides.

Assessing the effects of pesticide exposure poses considerable challenges, as in vitro and in vivo experiments are not only expensive, but raise ethical concerns. To address these issues, in silico methods, such as pharmacophore modeling and virtual screening, can offer a highly effective complement. These computational approaches provide predictive insights, allowing for the early identification of potential hazards. By enabling the targeted prioritization of biological tests, they reduce the reliance on animal experiments and promote efficient resource deployment.

In this study, we developed and optimized pharmacophore-based models for potential jakibs, leveraging known jakibs from the literature as training data. These models are designed to identify compounds with structural features that align with the binding site of the target protein, allowing for the efficient virtual screening of large compound databases. By prioritizing compounds that match the pharmacophore model, the likelihood of testing compounds that exhibit the desired biological activity is increased.

Moreover, pharmacophore-based virtual screening (PBVS) facilitates the discovery of biologically active compounds by enabling early-stage predictions of both the desired biological activities and the potential off-target effects or toxicological risks. This approach is not limited to pharmaceutical research but is equally valuable in the development and monitoring of agrochemicals, such as herbicides or insecticides, where high target specificity and minimal impact on non-target organisms are essential. PBVS aids in the prioritization of candidates for biological testing, thereby accelerating the overall development process and lowering costs in both pharmaceutical and agrochemical contexts [21].

## 2. Results

### 2.1. Datasets

**Figure 1:**
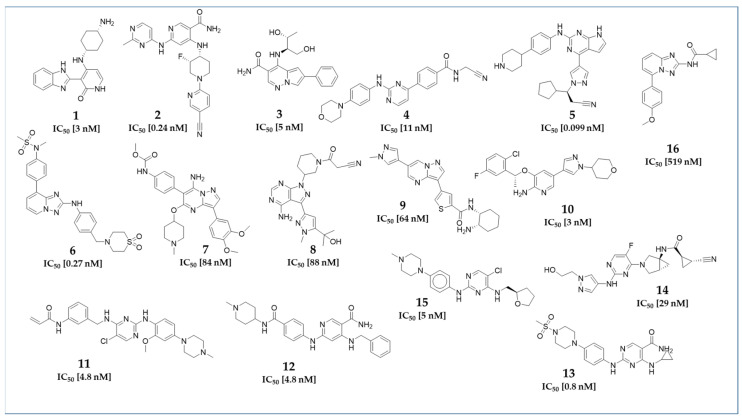
2D structures of the pharmacophore modeling training compounds for JAK1 (**1** [25], **2** [26], **3** [27], **4** [28]) JAK2 (**5** [29], **6** [30], **7** [31], **8** [32], **9** [33], **10** [34]), JAK3 (**11** [35], **12** [36], **13** [30]), and TYK2 (**14** [37], **15** [38], **16** [39]) used to develop the shown pharmacophore models, along with their corresponding IC_50_ values.

As a basis for model development, literature-based datasets were assembled. In order to train a model to distinguish active and inactive compounds, data sets of active compounds (ACs) and inactive compounds (IAs) were collected. To qualify a model’s performance in a larger database, sets of probable inactive compounds, so-called decoys (DCs), were generated. The dataset sizes of ACs, IAs, and DCs corresponding to the assigned kinase subtype are shown in Table 1. Exemplary 2D structures of representative ACs used in this project are shown in Figure 1.

### 2.2. Pharmacophore Modeling

“A pharmacophore is the ensemble of steric and electronic features that is necessary to ensure the optimal supramolecular interactions with a specific biological target and to trigger (or block) its biological response” [22]. Therefore, pharmacophore modeling is based on the theory that shared chemical functionalities and similar localization of features lead to biological activity on the same target. The chemical properties of a molecule that is able to interact with its ligand are shown in the pharmacophore model as geometric features, such as spheres and vectors, as illustrated in Figure 2, Figure 3, Figure 4 and Figure 5, and in the Supplementary Materials (Figures S1–S29).

In this project, we use structure-based (SB) and ligand-based (LB) pharmacophore modeling as part of the virtual screening approach [23]. SB modeling involves generating models based on the 3D structure of the target protein, identifying key binding sites and interactions. LB pharmacophore modeling, on the other hand, is based on the alignment of known ligands’ structures to identify common features critical for binding to the target. Both approaches are used to predict potential interactions between compounds and the target protein during virtual screening [23].

The models in this project consist of hydrogen bond donors (HBDs), hydrogen bond acceptors (HBAs), aromatic interactions (AIs), hydrophobic contacts (HCs), residue bonding points (RBPs), and exclusion volumes (Xvols). For better clarity, Xvols are specified numerically in this publication instead of being shown graphically.

**Figure 2:**
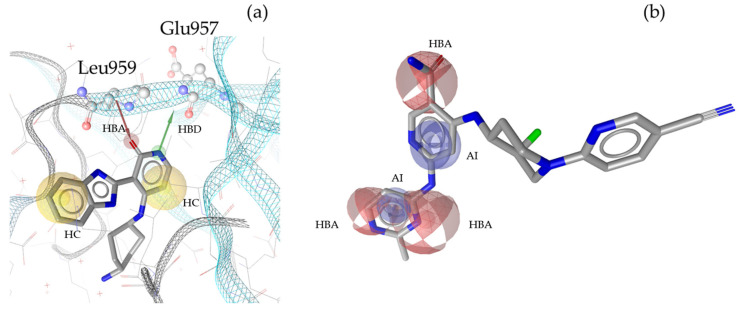
Two exemplary pharmacophore models for JAK1, illustrating key interactions and structural features. (**a**) JAK1_SB1 developed from the X-ray structure PDB: 5HX8 [25] in complex with its co-crystallized ligand compound **1** [25]. The model consists of one HBD with Glu957 and one HBA with Leu959, as well as two HCs. Furthermore, the model includes 66 Xvols. (**b**) Shows LB pharmacophore model JAK1_LB1 in complex with compound **2** [26]. This model was generated through alignment and merging of features from compound **2** [26], compound **3** [27], and compound **4** [28] JAK1_LB1 includes three HBAs, two AIs, and forty-seven Xvols. Chemical features are color-coded: HBDs—green, HBAs red, HCs—yellow, AIs—blue.

**Figure 3:**
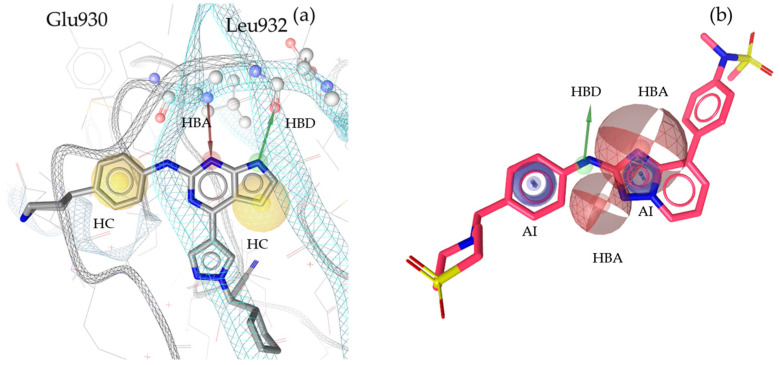
Two exemplary pharmacophore models for JAK2, illustrating key interactions and structural features. (**a**) JAK2_SB1, developed from the X-ray structure PDB: 6VNB [29] in complex with its co-crystallized ligand compound **5** [29]. This model consisted of one HBD with Leu932, one HBA with Glu930, two HCs, and nine Xvols. (**b**) Shows the LB pharmacophore model JAK2_LB1 in complex with compound **6** [30]. This model was generated through alignment and merging of features from compound **7** [31], compound **8** [32], compound **9** [33], and compound **10** [37]. JAK2_LB1 includes one HBD, two HBAs, two AIs, and nine Xvols. Chemical features are color-coded: HBDs—green, HBAs red, HCs—yellow, AIs—blue.

**Figure 4:**
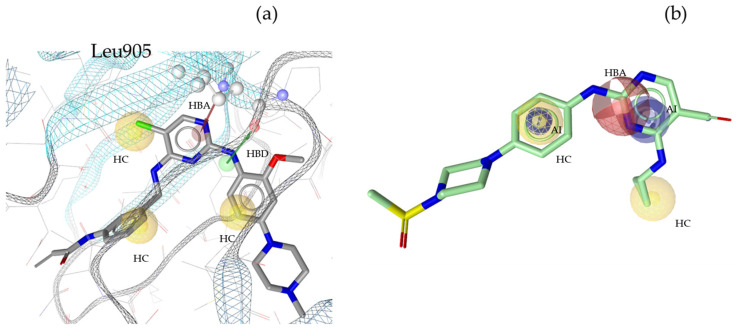
Two pharmacophore models for JAK3, illustrating key interactions and structural features. (**a**) JAK3_SB1, developed from the X-ray structure PDB: 4Z16 [35] in complex with its co-crystallized ligand compound **11** [35]. This model includes one HBD with Leu905, one HBA, three HCs, and twenty Xvols. (**b**) Shows LB pharmacophore model JAK3_LB1 in complex with compound **12**. This model was generated through alignment and merging of features from compound **12** [36] and compound **13** [40]. JAK3_LB1 includes one HBA, two AIs, two HCs, and twenty-nine Xvols. Chemical features are color-coded: HBDs—green, HBAs red, HCs—yellow, AIs—blue.

**Figure 5:**
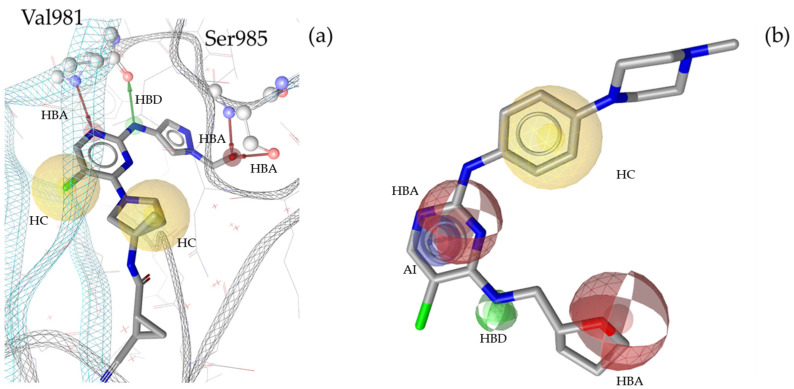
Two pharmacophore models for TYK2, illustrating key interactions and structural features. (**a**) TYK2_SB1, based on the X-ray structure PDB: 6VNS [37] in complex with the co-crystallized ligand compound **14** [37]. This model consists of one HBD with Val981, one HBA and two HBAs directed to Ser985, two HCs, and twenty-six Xvols. (**b**) Shows LB pharmacophore model TYK2_LB1 in complex with compound **15** [38]. This model was generated through alignment and merging of features from compound **15** [38] and compound **16** [39] TYK2_LB1 includes one HBD, two HBAs, one HC, one AI, and thirty-five Xvols. Chemical features are color-coded: HBDs—green, HBAs red, HCs—yellow, AIs—blue.

Multiple pharmacophore models are used to capture the diversity of compounds in the training set. In this project, both structure-based (SB) and ligand-based (LB) pharmacophore models were generated [24]. In total, eight models for JAK1 (four SB + four LB), ten models for JAK2 (two SB + eight LB), ten models for JAK3 (three SB + seven LB), and nine models for TYK2 (three SB + six LB) were generated. Examples of SB- and LB-based models in complex with identified training set hits are presented in Figure 2, Figure 3, Figure 4 and Figure 5.

The 2D structures of the literature-known Janus kinase inhibitors, used specifically for the generation of the pharmacophore models presented in the manuscript, are shown in Figure 1.

These molecules served as the basis for the generation of the displayed pharmacophore models. The complete datasets used for the development and training of the models are provided in the Supplementary Materials section (Tables S1–S8). The remaining pharmacophore models, along with the structures they are based on, are presented in the Supplementary Materials (Figures S1–S29).

### 2.3. Theoretical Evaluation of the Generated Pharmacophore Models

**Figure 6:**
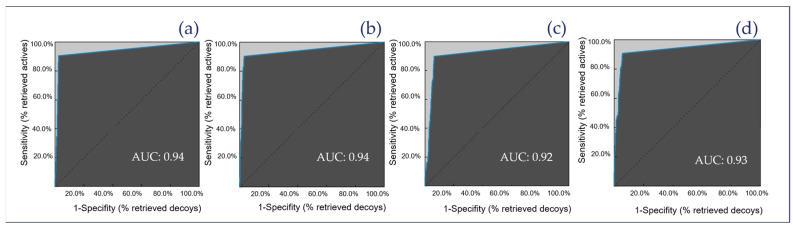
ROC curves of the overall models, including the AUC. Panel (**a**) shows JAK1, panel (**b**) shows JAK2, panel (**c**) shows JAK3, and panel (**d**) shows TYK2.

Each model was optimized to maximize the identification of ACs from the training set while excluding a high number of IAs and DCs. Table 1 presents the results of the theoretical evaluation for the overall models of JAK1, JAK2, JAK3, and TYK2. The theoretical evaluation of the generated pharmacophore models focused on key performance metrics, such as model accuracy, enrichment factor (EF), and yield of active compounds (YoA). These metrics were derived from the classification of compounds into true positives (TPs), false positives (FPs), true negatives (TNs), and false negatives (FNs). Each pharmacophore model was systematically optimized to ensure the highest possible identification of active ACs from the training set. Simultaneously, the models were refined to maximize their ability to distinguish IAs and DCs, ensuring both robustness and precision in virtual screening. Figure 6a–d show the overall receiver operating characteristic (ROC) curves for these models, illustrating their predictive performance as well as the area under the curve (AUC). The results for each individual model from this evaluation process are provided in the Supplementary Materials (Tables S11–S14). The ROC curves of the individual models are shown in the Supplementary Materials (Figures S30–S33).

### 2.4. Identified Pesticides During Virtual Screening Campaign

**Figure 7:**
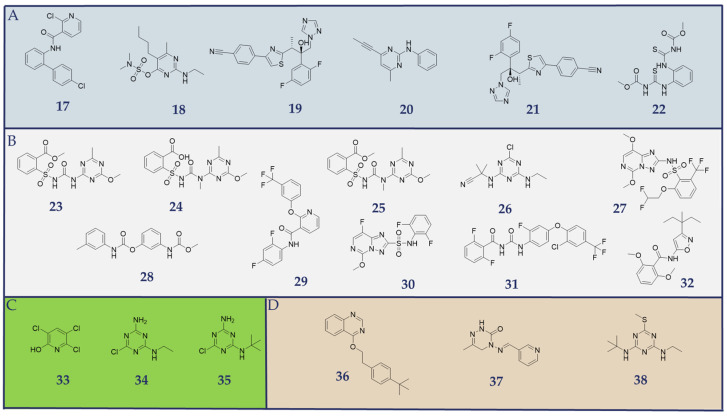
Identified pesticides from the virtual screening that are listed as qualitatively detected in the Human Metabolome Database. The hit list included fungicides (Section (**A**)), such as boscalid (**17**), bupirimate (**18**), isavuconazole (**19**), mepanipyrim (**20**), ravuconazole, (**21**), thiophanate-methyl (**22**). (Section (**B**)) comprises herbicides, including metsulfuron-methyl (**23**), tribenuron (**24**), tribenuron-methyl (**25**), cyanazine (**26**), penoxsulam (**27**), phenmedipham (**28**), diflufenican (**29**), florasulam (**30**), flufenoxuron (**31**), and isoxaben (**32**). (Section (**C**)) represents metabolites, such as 3,5,6-trichloro-2-pyridinol (**33**), deisopropylatrazine (**34**), and desethylterbutylazine (**35**). (Section (**D**)) includes insecticides, such as fenazaquin (**36**), pymetrozine (**37**), and terbutryn (**38**).

**Figure 8:**
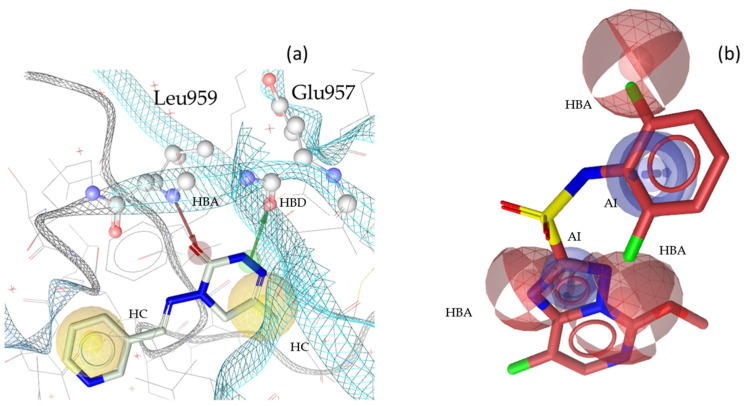
Exemplary virtual hits for JAK1. (**a**) Identified insecticide **36** (pymetrozine) in complex with model JAK1_SB1. (**b**) Identified herbicide **30** (florasulam) in model JAK1_LB1. Chemical features are color-coded: HBDs—green, HBAs red, HCs—yellow, AIs—blue.

**Figure 9:**
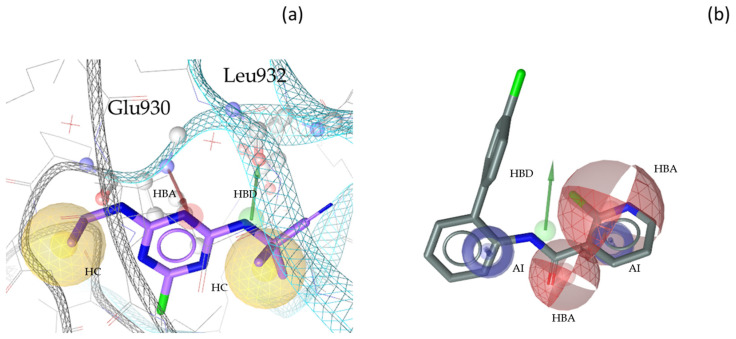
Exemplary virtual hits for JAK2. (**a**) Identified insecticide **27** (cynacine) in complex with model JAK2_SB1. (**b**) The fungicide **17** (boscalid) mapping model JAK2_LB1. Chemical features are color-coded: HBDs—green, HBAs red, HCs—yellow, AIs—blue.

**Figure 10:**
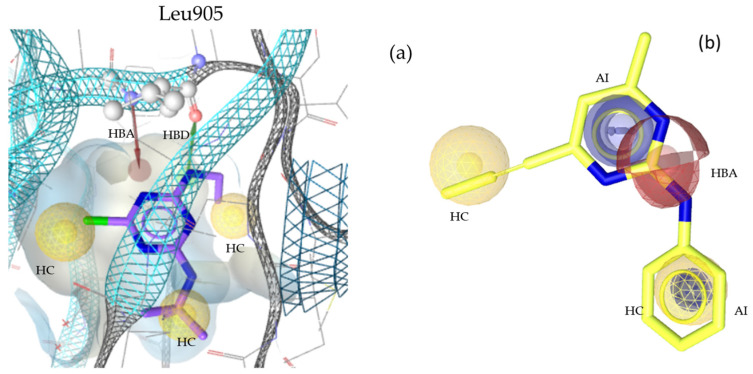
Exemplary virtual hits for JAK3. (**a**) The identified herbicide **26** (cyanazine) in complex with model JAK3_SB1. (**b**) The fungicide **20** (mepanipyrim) in model JAK3_LB1. Chemical features are color-coded: HBDs—green, HBAs red, HCs—yellow, AIs—blue.

**Figure 11:**
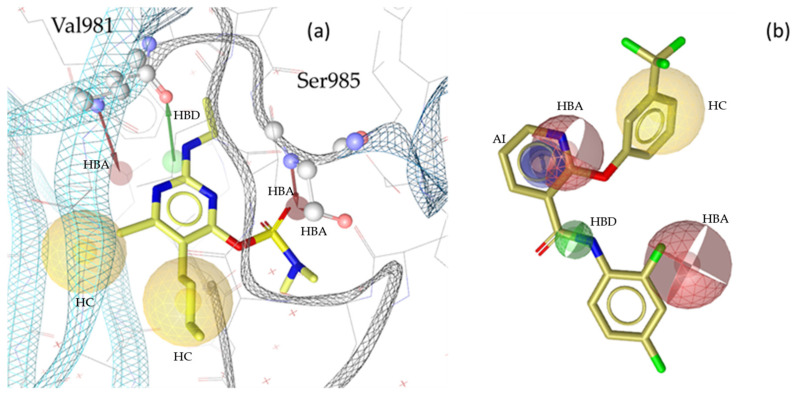
Exemplary virtual hits for TYK2. (**a**) The identified fungicide **18** (bupirimate) fitting into model TYK2_SB1. (**b**) The herbicide **28** (diflufenican) mapping to model TYK2_LB1. Chemical features are color-coded: HBDs—green, HBAs red, HCs—yellow, AIs—blue.

The pharmacophore models identified 64 pesticides potentially inhibiting one or more JAKs by virtually screening the LUXPEST database [41]. A subsequent comparison with the Human Metabolome Database [42] revealed that 22 substances identified in the virtual screening have been qualitatively detected in the human body. The 2D structures of these substances are presented in Figure 7. Examples of identified pesticides in complex with their respective models, as described in detail in Section 2.2, are illustrated in Figure 8, Figure 9, Figure 10 and Figure 11. The comprehensive list of all 64 identified pesticides obtained from the virtual screening of the LUXPEST [41] database assigned to the corresponding JAK model, is provided in the Supplementary Materials in Tables S15–S18.

## 3. Discussion

PBVS offers a rapid and cost-efficient approach to early drug discovery, providing significant advantages over traditional high-throughput screening (HTS). By leveraging pharmacophore models, PBVS allows for the efficient prioritization of promising compounds from vast chemical libraries, reducing both time and costs while minimizing environmental impact. This approach filters out inactive or toxic substances before they reach experimental evaluation, streamlining the drug discovery process [43].

High-quality pharmacophore models can swiftly screen large, chemical databases, facilitating the identification of potential active compounds and enhancing the exploration of chemical space. This enables researchers to prioritize compounds for further experimental validation, significantly reducing resource consumption and time compared to conventional methods [44].

However, the accuracy of PBVS is highly dependent on the careful construction of well-validated models and the use of high-quality training datasets, as the reliability of pharmacophore models is intrinsically linked to the data from which they are derived.

Jakibs are widely recognized for their ability to suppress the immune system. Patients taking these inhibitors display increased risks of infections, including herpes, influenza, and fungal infections like pneumocystis. Respiratory and urinary tract infections are also more frequent, along with adverse events, such as joint and musculoskeletal disorders. Reports of malignant neoplasms, including hematopoietic, skin, and respiratory cancers, are also more prevalent. Additionally, this substance class is associated with an increased risk of venous thromboembolism compared to placebo or tumor necrosis factor (TNF) inhibitors [20,45,46,47,48].

In Europe, the risk assessment of pesticides is hindered by significant shortcomings within the regulatory framework. Despite the EU Pesticide Regulation 1107/2009 (http://data.europa.eu/eli/reg/2009/1107/oj, accessed on 14 April 2025) being one of the strictest globally, there is still room for improvement considering chronic toxicity and long-term effects [49]. However, there is much to consider. A 2024 U.S. study examined the link between agricultural pesticide use and cancer incidence by analyzing county-level data on pesticides, cancer rates, and factors like smoking and population demographics. Using latent class analysis, the researchers identified pesticide use profiles and assessed their impact on cancer incidence, while considering factors like land use and social vulnerability. The study highlighted regional trends between pesticide exposure and cancer. Atrazine, an herbicide with a 1,3,5-triazine core structure, was consistently identified as a major contributor to increased cancer risk, particularly for colon cancer. These findings emphasize the varied effects of pesticides on different cancer types across regions [50]. Atrazine was also identified as a virtual hit for JAK2, JAK3, and TYK2; however, it was not present in the HMD. The JAK model collection identified similar pesticides from this structural scaffold, including cyanazine (**26**), metsulfuron-methyl (**23**), tribenuron (**24**), tribenuron-methyl (**25**), and terbutryn (**37**). Additionally, the virtual hit and fungicide boscalid (**17**) is associated with higher risks for leukemia, non-Hodgkin lymphoma, and pancreatic cancer [50].

The Food Safety Commission of Japan assessed the anilinopyrimidine fungicide mepanipyrim (**20**) based on various studies. Results showed hepatocellular hypertrophy, liver degeneration, and increased kidney weight in rats. A genotoxic mechanism was ruled out, and the exact mechanism of cancer development remains unclear; therefore, based on our findings, further investigations into the inhibition of JAKs are recommended [51].

Diflufenican (**29**) is a selective herbicide widely used in agriculture to control broadleaf weeds in crops such as corn and soybeans. As a phenyl ether herbicide, it exhibits moderate toxicity at high exposure levels, potentially leading to liver issues in animals. While current data suggests no increased cancer risk or immune-related effects, further studies are recommended due to its identification by pharmacophore models of all four JAK subtypes in this project. Additionally, the degradation product TFA (trifluoroacetic acid) raised concerns because of its persistence in the environment, acting as a “forever chemical” that can accumulate in both ecosystems and human tissues, potentially posing long-term environmental and health risks [52].

Counter to the immune-suppressive hypothesis, there are also studies suggesting that certain pesticides, such as thiophanate-methyl (**22**), activate immune responses rather than suppressing them. These substances stimulate inflammatory pathways, enhance macrophage proliferation, and promote cytokine production, including interleukins (IL) IL-1β, IL-6, TNF-α, and interferon-gamma. This underscores the dual nature of pesticide impacts, highlighting their capacity to both suppress and activate immune functions depending on specific mechanisms of action and exposure contexts [53].

Penoxsulam (**27**) is a herbicide from the sulfonamide class that inhibits acetolactate synthase in various weeds, aquatic plants, and grasses [54]. The EPA classifies it as having potential carcinogenicity, based on the observation of mononuclear cell leukemia in rats during carcinogenicity testing. A study conducted by D.M. Patel et al. examined the relationship between agricultural pesticides and the risk of childhood leukemia in Denmark. The herbicides phenmedipham (**28**) and tribenuron-methyl (**25**) were identified as potential risk factors. While higher applications of phenmedipham (**28**) were associated with an increased risk of childhood leukemia, the findings were not statistically significant. Both herbicides are classified as possible human carcinogens, highlighting concerns about their use in residential areas [55].

Interesting anti-inflammatory effects were reported for the herbicide cyanazine (**23**). It effectively suppressed lipopolysaccharide-induced increases in key pro-inflammatory cytokines, including TNF-α and IL-6, thereby inhibiting gonadal inflammation [56]. As the relevant cytokines are also prominently involved in the JAK signaling pathway, it would be interesting to follow up with in vitro experiments on this compound.

Significant knowledge gaps remain in understanding the off-target effects of pesticides, particularly in their interaction with molecular pathways, such as JAK signaling. While computational approaches, like pharmacophore-based virtual screening, offer valuable insights into potential risks, biological validation of these models is essential to confirm their accuracy. The next step is the biological validation of the virtual hits through a single protein assay, providing evidence for these predictions. Additional steps, such as cell-based assays, may offer more comprehensive insights, improving our understanding of the broader impacts of pesticide exposure. It could also be that pesticides applied in mixtures act synergistically, amplifying their biological effects. Such synergistic effects have already been observed in salmon, where mixtures of pesticides in the water led to severe disruptions in their behavior and survival. Similar effects may also occur in humans, particularly with prolonged or repeated exposure. However, this remains an area requiring further research, as pesticides will be still essential in the future to enhance crop yields and food safety [57].

It takes a long time for the effects of pesticide use to manifest in epidemiological studies, like the ones mentioned above. It would therefore be desirable to anticipate potential chronic interference with human, animal, and crop organisms already in the discovery process. Computational methods, as presented in this study, could aid in identifying potentially problematic molecules.

## 4. Materials and Methods

### 4.1. Generation of Databases

A dataset of ACs and IAs was compiled for the individual targets JAK1, JAK2, JAK3, and TYK2, using data from ChEMBL (European Bioinformatics Institute, EMBL-EBI, Hinxton, United Kingdom; https://www.ebi.ac.uk/chembl/, accessed on 14 April 2025) PubChem (National Center for Biotechnology Information, Bethesda, MD, USA; https://pubchem.ncbi.nlm.nih.gov/, accessed on 14 April 2025), and the Protein Data Bank (Research Collaboratory for Structural Bioinformatics, RCSB, Piscataway, NJ, USA; https://www.rcsb.org/, accessed on 14 April 2025) [58,59,60]. Additionally, a decoy set was generated using the online tool Directory of Useful Decoys (DOCK group, University of California San Francisco, San Francisco, CA, USA; https://dude.docking.org/, accessed on 14 April 2025) [61], comprising random structures assumed to be inactive but with similar physicochemical properties to the active compounds, used to evaluate model restrictiveness. Active compounds were limited to those with an IC_50_ ≤ 1000 nM, while inactive compounds were defined as those with an IC_50_ > 40.000 nM for JAK1 and >50.000 for JAK2, JAK3, and TYK2. Compounds with IC_50_ values in this intermediate range were subsequently excluded from the dataset. Lists detailing the manually selected ACs as well as IAs is given in the Supplementary Materials (Tables S1–S8) [19,23,24,25,26,27,29,30,31,32,33,34,36,37,38,62,63,64,65,66,67,68,69,70,71,72,73,74,75,76,77,78,79,80,81,82,83,84,85,86,87,88,89,90,91,92,93,94,95,96,97,98,99,100,101,102,103,104,105,106,107,108,109,110,111,112,113,114,115,116,117,118,119,120,121,122,123,124,125,126,127,128,129,130,131,132,133,134,135,136,137,138,139,140,141,142,143,144,145,146,147,148,149,150,151,152,153,154,155,156,157,158,159,160,161,162,163,164,165,166,167,168,169,170,171,172,173,174,175,176,177,178,179,180,181,182,183,184,185,186,187,188,189,190,191,192,193,194,195,196,197,198,199,200,201,202,203,204,205,206,207,208,209,210,211,212,213,214,215,216,217,218,219,220,221,222,223,224,225,226,227,228,229,230,231,232,233,234,235,236,237,238,239,240,241,242,243,244,245,246,247,248,249,250,251,252,253,254,255,256,257,258,259,260,261,262,263,264,265,266,267,268,269,270,271,272,273,274,275,276,277,278,279,280,281,282,283,284,285,286,287,288,289,290,291,292,293,294,295,296,297,298,299,300,301,302,303,304,305,306,307,308,309,310,311,312]. Due to the large number of references [70,71,72,73,74,75,76,77,78,79,80,81,82,83,84,85,86,87,88,89,90,91,92,93,94,95,96,97,98,99,100,101,102,103,104,105,106,107,108,109,110,111,112,113,114,115,116,117,118,119,120,121,122,123,124,125,126,127,128,129,130,131,132,133,134,135,136,137,138,139,140,141,142,143,144,145,146,147,148,149,150,151,152,153,154,155,156,157,158,159,160,161,162,163,164,165,166,167,168,169,170,171,172,173,174,175,176,177,178,179,180,181,182,183,184,185,186,187,188,189,190,191,192,193,194,195,196,197,198,199,200,201,202,203,204,205,206,207,208,209,210,211,212,213,214,215,216,217,218,219,220,221,222,223,224,225,226,227,228,229,230,231,232,233,234,235,236,237,238,239,240,241,242,243,244,245,246,247,248,249,250,251,252,253,254,255,256,257,258,259,260,261,262,263,264,265,266,267,268,269,270,271,272,273,274,275,276,277,278,279,280,281,282,283,284,285,286,287,288,289,290,291,292,293,294,295,296,297,298,299,300,301,302,303,304,305,306,307,308,309,310,311,312], those that exclusively concern compounds of the training sets are separately continued and mentioned in the bibliography to the Supplementary Materials. The 3D structures for pharmacophore model training and virtual screening were generated from the isomeric SMILES codes, with a maximum of 200 conformers generated for each compound using the iCon BEST [313] algorithm in LigandScout 4.4.5 (Inte:Ligand GmbH, Vienna, Austria) [314].

### 4.2. Pharmacophore Model Generation

All models were generated and optimized using LigandScout software version 4.4.5 [314]. SB models were generated using protein–ligand complex structures, while LB models were created by aligning multiple bioactive compounds [24]. For SB modeling, X-ray crystal structures of the protein–ligand complexes were used, including JAK1 (PDB: 4FK6 [62], 6SMB [63], 5WO4 [64], 5HX8 [25]), JAK2 (PDB: 6VNB [29], 7TEU [65]), JAK3 (PDB: 3ZEP [66], 4Z16 [35], 5TTV [67], 5LWM [68]), and TYK2 (PDB: 6VNS [37], 3LXN [315], 3NZ0 [316]). The selection of crystal structures was guided by quality criteria, including a resolution below 3 Å and highly defined electron density within the binding pocket. Crystal structures that generated three or fewer features using automatic generation with standard settings [314] were excluded. For LB modeling, the merged feature mode was applied to align the selected molecules in 3D from the active compound data set. During the optimization process, each pharmacophore feature was individually refined to maximize the detection of ACs, while minimizing the identification of IAs and DCs. To optimize the models, each feature was manually optimized and adjusted during subsequent screening stages. Features that did not enhance model selectivity were discarded, while Xvols were added or removed as needed, and feature tolerances were fine-tuned to maximize model performance.

### 4.3. Theoretical Validation

The assessment metrics calculated in this study encompassed sensitivity (a), specificity (b), accuracy (c), YoA (d), EF (e), and (ROC) curve. The ROC curve is a plot used to evaluate the performance of a classification model. It depicts the relationship between the true positive rate (TPR) and the false positive rate (FPR), with TPR on the y-axis and FPR on the x-axis. AUC is a metric derived from the ROC curve that represents a model’s ability to distinguish between positive and negative classes. AUC values range from 0 to 1, with higher values indicating better classification performance.

Continuous monitoring of these metrics facilitated the refinement process [21]. Pharmacophore models that did not meet performance criteria (EF < 4) were excluded. The overall performance of all JAK models, according to their subtype, is presented in Table 1, while the results of the individual models for each kinase are provided Tables S11–S14.

(a) Sensitivity = number of ACs identified by the model/number of ACs in the dataset
(b) Specificity = number of ACs not identified by the model/number of IAs in the dataset
(c) Accuracy = (number of TP/number of TN)/number of all the compounds in the database
(d) YoA = number of TP/number of total hits
(e) EF = YoA/(number of ACs in the database/number of all compounds in the database)

### 4.4. Virtual Screening

The LUXPEST S69 a Pesticide Screening List for Luxembourg (https://www.norman-network.com/nds/SLE/) Les Garennes sur Loire, France (28 May 2020) [41], containing 386 pesticides, was screened after being converted into a 3D database using LigandScout 4.4.5. Using iCon BEST [313], 400 conformers were generated per pesticide, following the same approach used in the creation of the training sets, as outlined in Section 4.1. An overview of all identified hits is included in the Tables S15–S18.

The identified pesticides were cross-referenced with the Human Metabolome Database to determine whether these compounds have been qualitatively detected in the human body [42].

## Figures and Tables

**Table 1:** Theoretical evaluation results of the overall generated pharmacophore models of JAK1, JAK2, JAK3, and TYK2 focused on model EF, YoA, along with TPs, FPs, TNs, and FNs.

Overall Evaluation	JAK1 TOTAL (8 Models)	JAK2 TOTAL (10 Models)	JAK3 TOTAL (10 Models)	TYK2 TOTAL (9 Models)
active hits/TPs	95	167	116	68
inactive hits	0	15	2	40
decoy hits	79	75	292	136
FPs	79	90	294	176
TNs	3232	2799	4247	2935
number of ACs in database	105	185	129	75
number of IAs in database	48	49	42	61
number of DCs in database	3263	2840	4499	3050
Model Accuracy	0.97	0.96	0.93	0.94
YoA	0.55	0.65	0.28	0.28
EF	17.76	10.80	10.24	11.84
sensitivity	0.90	0.90	0.86	0.91
specificity	1.00	0.99	1.00	1.00

## Data Availability

The data presented in this study are available in the Supplementary Materials. If further data are required, they are available from the corresponding author upon request.

## Supplementary Materials

The following supporting information can be downloaded at: https://www.mdpi.com/article/10.3390/molecules30102183/s1. Figure S1: JAK1_SB2; Figure S2: JAK1_SB3; Figure S3: JAK1_SB4; Figure S4: JAK1_LB2; Figure S5: JAK1_LB3; Figure S6: JAK1_LB4; Figure S7: JAK2_SB2; Figure S8: JAK2_LB2; Figure S9: JAK2_LB3; Figure S10: JAK2_LB4; Figure S11: JAK2_LB5; Figure S12: JAK2_LB6; Figure S13: JAK2_LB7; Figure S14: JAK2_LB8; Figure S15: JAK3_SB2; Figure S16: JAK3_SB3; Figure S17: JAK3_SB4; Figure S18: JAK3_LB2; Figure S19: JAK3_LB3; Figure S20: JAK3_LB4; Figure S21: JAK3_LB5; Figure S22: JAK3_LB6; Figure S23: TYK2_SB2; Figure S24: TYK2_SB3; Figure S25: TYK2_LB2; Figure S26: JAK2_LB3; Figure S27: TYK2_LB4; Figure S28: TYK2_LB5; Figure S29: TYK2_LB6; Figure S30: ROC-curves JAK1; Figure S31: ROC-curves JAK2; Figure S32: ROC-curves JAK3; Figure S33: ROC-curves TYK2. Table S1: JAK1 active compounds; Table S2: JAK1 inactive compounds; Table S3: JAK2 active compounds; Table S4: JAK2 inactive compounds; Table S5: JAK3 active compounds; Table S6: JAK3 inactive compounds; Table S7: TYK2 active compounds; Table S8: TYK2 inactive compounds; Table S9: Pharmacophore features overview; Table S10: Amino acid interactions of structure-based models; Table S11: Theoretical evaluation results JAK1; Table S12: Theoretical evaluation results JAK2; Table S13: Theoretical evaluation results JAK3; Table S14: Theoretical evaluation results TYK2; Table S15: Pesticide Hits JAK1; Table S16: Pesticide Hits JAK2; Table S17: Pesticide Hits JAK3; Table S18: Pesticide Hits TYK2.
