# Supplementary material for: Pharmacophore Modeling of Janus Kinase Inhibitors: Tools for Drug Discovery and Exposition Prediction
Source: Molecules. 2025 May 16;30(10):2183. doi: 10.3390/molecules30102183 (PMC12114199; doi:10.3390/molecules30102183)
Supplement: Supplementary file 1 [file molecules-30-02183-s001.zip › molecules-3526396-supplementary.pdf]

molecules

Supplementary Information

1. Data set assembly 4
1.1 actives JAK1 4
1.2 inacctives JAK1 9
1.3 actives JAK2 14
1.4 inactives JAK2 25
1.5 actives JAK3 27
1.6.inactives JAK3 35
1.7 actives TYK2 37
1.8 inactives TYK2 41
2. Pharmacophore models 45
2.1 JAK1 MODELS 45
2.1.2 JAK1_SB2 45
2.1.3 JAK1_SB3 45
2.1.1 JAK1_SB4 46
2.1.6 JAK1_LB2 46
2.1.5 JAK1_LB3 47
2.1.8 JAK1_LB4 47
2.2 JAK2 MODELS 48
2.2.2 JAK2_SB2 48
2.2.4 JAK2_LB2 48
2.2.5 JAK2_LB3 49
2.2.6 JAK2_LB4 49
2.2.7 JAK2_LB5 49
2.2.8 JAK2_LB6 50
2.2.9 JAK2_LB7 51
2.2.10 JAK2_LB8 51
2.3 JAK3 MODELS 52
2.3.1 JAK3_SB2 52

 https://doi.org/10.3390/10.3390/molecules30102183

2.3.3 JAK3_SB3 52
2.3.4 JAK3_SB4 53
2.3.6 JAK3_LB2 53
2.3.7 JAK3_LB3 54
2.3.8 JAK3_LB4 54
2.3.9 JAK3_LB5 55
2.3.10 JAK3_LB6 55
2.4 TYK2 MODELS 56
2.4.2 TYK2_SB2 56
2.4.3 TYK2_SB3 56
2.4.5 TYK2_LB2 57
2.4.6 TYK2_LB3 57
2.4.4 TYK2_LB4 58
2.4.8 TYK2_LB5 59
2.4.9 TYK2_LB6 59
2.5 Pharmacophore features 60
2.6. Amino acid interactions of the structure-based models 61
3. Theoretical evaluation 63
3.1 JAK1 63
3.1.1 Results of the theoretical evaluation process (JAK1) 63
3.1.2 ROC-curves JAK1 64
3.2 JAK2 65
3.2.1 Results of the theoretical evaluation process (JAK2) 65
3.2.2 ROC-curves JAK2 65
3.3 JAK3 66
3.3.1 Results of the theoretical evaluation process (JAK3) 66
3.3.2 ROC-curves JAK3 66
3.4 TYK2 67
3.4.1 Results of the theoretical evaluation process (TYK2) 67
3.4.2 ROC-curves TYK2 68
4. Idetified Pesticides 69
4.1.JAK1 HITS 69
4.2 JAK2 HITS 70
4.3 JAK3 HITS 71

4.4 TYK2 HITS 72

5. References: 73

1. Data set assembly

1.1 actives JAK1

Table S1 presents the training set of active Janus kinase inhibitors from the literature for Janus kinase 1, including the ChEMBLl ID, SMILES code, and IC50 values.

Target	Compound	SMILES	Values	Ref
JAK1_AC	CHEMBL2105759	CCS(=O)(=O)N1CC(CC#N)(n2cc(-c3ncnc4[nH]ccc34)cn2)C1	4	19
JAK1_AC	CHEMBL4466236	Cc1nccc(Nc2cc(N[1]3CCN(c4ccc(C#N)cn4)C[1]3F)c(C(N)=O)cn2)n1	0.24	25
JAK1_AC	CHEMBL3780091	N[1]1CC[1](Nc2cc[nH]c(=O)c2-c2nc3ccccc3[nH]2)CC1	3	25
JAK1_AC	CHEMBL3593777	N#Cc1ccc(N2CC[1](Nc3c(C(N)=O)cnc4[nH]ccc34)[1](F)C2)nc1	456	26
JAK1_AC	CHEMBL4447360	C[1](O)[1](CO)Nc1c(C(N)=O)cnn2cc(-c3ccccc3)cc12	5	26
JAK1_AC	CHEMBL1078178	N#CCNC(=O)c1ccc(-c2ccnc(Nc3ccc(N4CCOCC4)cc3)n2)cc1	11	28
JAK1_AC	CHEMBL4568087	Cn1cc(-c2cnc3c(-c4csc(C(=O)N[1]5CCCC[1]5N)c4)cnn3c2)cn1	220	33
JAK1_AC	CHEMBL4637163	N#CC[1](C1CCCC1)n1cc(-c2ncnc3c2CCN3)cn1	459	34
JAK1_AC	CHEMBL3601120	C=CC(=O)Nc1cccc(CNc2nc(Nc3cnn(CCO)c3)ncc2Cl)c1	11	35
JAK1_AC	CHEMBL4644578	C[1]1[1]2CN(c3nc(Nc4cnn(CCO)c4)ncc3F)C[1]12NC(=O)[1]1C[1]1C#N	6	37
JAK1_AC	CHEMBL3360349	O=C(NCCc1ccn(-c2ccccc2)n1)c1ccc(-c2cccc3nc(NC(=O)C4CC4)nn23)cc1	0.6	38
JAK1_AC	CHEMBL3360356	O=C(Nc1nc2cccc(-c3ccc(CN4CCC(F)(F)CC4)cc3)n2n1)C1CC1	253	39
JAK1_AC	CHEMBL2035044	CN1C/C=C/CCOc2cc(ccn2)-c2ccnc(n2)Nc2cccc(c2)C1	170	40
JAK1_AC	CHEMBL4441479	COc1nn(C)cc1Nc1ncc(C)c(-c2c[nH]c3c(NC(=O)c4cccnc4C)cccc23)n1	18	64
JAK1_AC	CHEMBL4446962	COc1nn(C)cc1Nc1ncc(C)c(-c2c[nH]c3c(NCc4ccc(C)nc4C)cccc23)n1	4	64
JAK1_AC	CHEMBL4071005	COc1cc(Nc2nn([1]3COCC[1]3C#N)cc2C(N)=O)ccc1Cl	0.1	65
JAK1_AC	CHEMBL4078799	Cn1cc(-c2ccc(Nc3nn([1]4COCC[1]4C#N)cc3C(N)=O)cc2)cn1	6	66

JAK1_AC	CHEMBL4083094	C=CC(=O)N1CC[1](C)[1](N(C)c2ncnc3[nH]ccc23)C1	988	69
JAK1_AC	CHEMBL1081484	COc1cc(Nc2nc3cccc(-c4ccc(C(=O)N5CCOCC5)c(F)c4)c3o2)cc(OC)c1OC	22	76
JAK1_AC	CHEMBL1089773	CN1CCN(c2ccc(-c3cnc4cccc(-c5cc(F)c(CN6CCOCC6)c(F)c5)c4n3)cc2)CC1	28	77
JAK1_AC	CHEMBL1171837	Cc1ccc(C(=O)Nc2ccc(CN3CCN(C)CC3)c(C(F)(F)F)c2)cc1C#Cc1cnc2cccnn12	32.2	79
JAK1_AC	CHEMBL3301606	CC1(C)CN(C(=O)c2ccc(-c3cccc4nc(NC(=O)C5CC5)nn34)cc2)C1	458	81
JAK1_AC	CHEMBL1784637	CNC(=O)c1ncc(C#Cc2cc(C(=O)Nc3ccc(CN4CCN(CCO)CC4)c(C(F)(F)F)c3)ccc2C)n1C	55.3	94
JAK1_AC	CHEMBL1789941	N#CC[1](C1CCCC1)n1cc(-c2ncnc3[nH]ccc23)cn1	6	95
JAK1_AC	CHEMBL1835153	NC(=O)c1cnc(N[1](C2CC2)C(F)(F)F)c2c1[nH]c1cc(-c3cnc(N)nc3)ccc12	457	97
JAK1_AC	CHEMBL1983268	CN1CCN(c2ccc(C(=O)Nc3n[nH]c4ccc(Cc5cc(F)cc(F)c5)cc34)c(NC3CCOCC3)c2)CC1	112	106
JAK1_AC	CHEMBL2062805	CN1CCN(c2ccc(Nc3nc4c(-c5ccc(S(C)(=O)=O)cc5)cccn4n3)cc2)CC1	22	111
JAK1_AC	CHEMBL2103874	CNS(=O)(=O)C[1]1CC[1](N(C)c2[nH]cnc3nccc2-3)CC1	196	112
JAK1_AC	CHEMBL21156	CC(C)(C)c1nc2c3ccc(F)cc3c3c(=O)[nH]ccc3c2[nH]1	15	115
JAK1_AC	CHEMBL2181312	Fc1cc2cc(c1)-c1ccnc(n1)Nc1ccc(OCCN3CCCC3)c(c1)COCC=CCOC2	380	122
JAK1_AC	CHEMBL2208034	O=C(Nc1ccc(-c2ccnc(Nc3ccc(N4CCOCC4)cc3)n2)cc1)[1]1CCCN1	134.3	123
JAK1_AC	CHEMBL2208035	O=C1CC[1](C(=O)Nc2ccc(-c3ccnc(Nc4ccc(N5CCOCC5)cc4)n3)cc2)N1	0.9	123
JAK1_AC	CHEMBL221959	C[1]1CCN(C(=O)CC#N)C[1]1N(C)c1ncnc2[nH]ccc12	15	125
JAK1_AC	CHEMBL4290597	COc1cc2c(=O)n([1]3CCCN(C(=O)CC#N)C3)c3c4cc[nH]c4ncc3c2cc1OC	456	127
JAK1_AC	CHEMBL2322135	c1cc2c(ncc3[nH]nc(C4CCCCC4)c32)[nH]1	3	129
JAK1_AC	CHEMBL2325897	N#C[1]1CCN(C(=O)[1](NC(=O)c2c[nH]c3ncc(C4CC4)nc23)C2CC2)C1	0.47	130
JAK1_AC	CHEMBL2443139	CC(C)Nc1ccc(Oc2cc(O)cc(O)c2-c2cc(C(=O)NC3CCN(C)CC3)no2)cc1	10	137

JAK1_AC	CHEMBL3116050	C[1](Nc1nc(Nc2cn(C)cn2)c2cc[nH]c2n1)c1ncc(F)cn1	2	143
JAK1_AC	CHEMBL3137308	NC(=O)c1cnc2[nH]ccc2c1N[1]1[1]2CC3C[1]1C[1](O)(C3)C2	459	146
JAK1_AC	CHEMBL4783938	CNC(=O)c1cnc2[nH]ccc2c1N[1]1CCN(Cc2ccc(Cl)cc2)[1](C)C1	0.145	150
JAK1_AC	CHEMBL3359927	C[1]1(F)CCC[1]1Nc1c(C(N)=O)cnn2cccc12	9	155
JAK1_AC	CHEMBL3622150	CCn1c(C(=O)N(C2CC2)C2CC2)cc2c3c(ncn3C)c(Nc3cc(C)[nH]n3)nc21	16	158
JAK1_AC	CHEMBL3589169	CN1CCN(C(=O)c2ccc3[nH]c4c(C(N)=O)cc(-c5ccc(Cl)cc5)cc4c3c2)CC1	458	159
JAK1_AC	CHEMBL3593591	C[1]1CCN(C(=O)CC#N)C[1]1n1c(=O)[nH]c2cnc3[nH]ccc3c21	457	160
JAK1_AC	CHEMBL3622134	CCn1c(C(=O)N(C2CC2)C2CC2)cc2c3c(ncn3C)c(Nc3nccs3)nc21	40	164
JAK1_AC	CHEMBL3644618	CCc1cc(O)c(F)cc1-c1ccc2c(-c3nc4c([nH]3)CCN(C(=O)c3ccc(F)cc3)C4)n[nH]c2c1	0.1	169
JAK1_AC	CHEMBL4068357	C[1]1CCN(C(=O)N2CCCC2)C[1]1N(C)c1ncnc2[nH]ccc12	37.5	169
JAK1_AC	CHEMBL4279720	C[1]1(NC#N)CCc2ccc(-c3ncnc4[nH]ccc34)cc21	5	170
JAK1_AC	CHEMBL3645121	CS(=O)(=O)N1CCN(c2ccc(Nc3ncc(C(N)=O)c(NC4CC4)n3)cc2)CC1	458	171
JAK1_AC	CHEMBL3652403	CN(c1ncnc2[nH]ccc12)[1]1CC[1](CS(=O)(=O)N2CCC[1](OC(=O)C(C)(C)C)C2)CC1	0.0227	175
JAK1_AC	CHEMBL3655103	CN(c1ncnc2[nH]ccc12)[1]1C[1](NS(=O)(=O)N2CCC(c3cc[nH]n3)CC2)C1	1	176
JAK1_AC	CHEMBL3658126	CNC(=O)c1ccc(Nc2ncc3c(n2)CCN(c2cc(NC(=O)c4cc(C(F)(F)F)ccn4)ccc2Cl)C3)cc1	12	177
JAK1_AC	CHEMBL3667467	C[1]1[1]2CN(S(C)(=O)=O)C[1]2C[1]1Nc1c(C(N)=O)cnn2cc(-c3cnc(N)nc3)cc12	0.2	180
JAK1_AC	CHEMBL4096145	CC1(C)[1](Nc2c(C(N)=O)cnn3cc(-c4ccccc4)cc23)CC[1]1(C)N	0.4	181
JAK1_AC	CHEMBL3671816	CC1(C)[1](Nc2c(C(N)=O)cnn3cc(-c4ccccc4)cc23)CC[1]1(C)O	0.1	182
JAK1_AC	CHEMBL3904952	COc1ncc(F)cc1[1]1CCCN1c1ccn2ncc(C(=O)NC3CCC(O)CC3)c2n1	546	183
JAK1_AC	CHEMBL3694581	Clc1cnc2nc1NCc1cccc(c1)OCCCc1cccc(c1)N2	452	185

JAK1_AC	CHEMBL3703048	C[1](O)(c1ccc(Nc2nn([1]3CC[1](N4CCC4)C[1]3C#N)cc2C(N)=O)cc1)C(F)(F)F	0.04	186
JAK1_AC	CHEMBL4643061	Cc1[nH]nc2[nH]c(=O)cc([1]3C[1]4C[1]3C[1]4CC#N)c12	77	187
JAK1_AC	CHEMBL3747305	COc1ccc(-c2nc3c(NCC4CCNCC4)c(Cl)cnc3[nH]2)cc1OC	459	188
JAK1_AC	CHEMBL3787112	Nc1ncnc2c1c(-c1cccc(O)c1)cn2[1]1C[1](CN2CCC2)C1	260	189
JAK1_AC	CHEMBL3814293	NC(=O)c1ccc2c(c1)nc([1]1CC[1](O)CC1)n2CCO	80	191
JAK1_AC	CHEMBL3815154	CNCCn1c2ccc(C(N)=O)cc2nc1[1]1CC[1](O)CC1	150	191
JAK1_AC	CHEMBL4239361	NC(=O)c1cnc2[nH]ccc2c1NC12CC3CC(CC(C3)C1)C2	110	195
JAK1_AC	CHEMBL388978	CN[1]1C[1]2O[1](C)([1]1OC)n1c3ccccc3c3c4c(c5c6ccccc6n2c5c31)C(=O)NC4	1	197
JAK1_AC	CHEMBL3900105	N#CC[1]1CC[1](c2nnn3cnc4[nH]ccc4c23)CC1	0.11	200
JAK1_AC	CHEMBL3911320	Nc1n[nH]c2ccc(C(=O)N3CCC[1]3c3ccc(Cl)cc3)cc12	90	202
JAK1_AC	CHEMBL3982495	N#CCC1(n2cc(-c3ncnc4[nH]ccc34)cn2)CN(C2CCN(C(=O)c3cc(Cl)cc(C(F)(F)F)n3)CC2)C1	5	206
JAK1_AC	CHEMBL3960628	N#CCC(=O)N1CCC2(CCC(Nc3nc(Nc4cnn(CC5CC5)c4)ncc3Cl)CC2)CC1	0.4	208
JAK1_AC	CHEMBL3944875	Cn1cc(Nc2ncc(Cl)c(NC3COC4C(NS(C)(=O)=O)COC34)n2)cn1	31	209
JAK1_AC	CHEMBL3953929	CCCCCC(CC#N)n1cc(-c2ncnc3[nH]ccc23)cn1	0.34	212
JAK1_AC	CHEMBL3963513	CN(c1cc(F)c(C#N)c(F)c1)[1]1CCN(c2ncnc3[nH]ccc23)C1	0.055	212
JAK1_AC	CHEMBL3975634	CN[1]1CC2OC([1]1OC)n1c3ccccc3c3c4c(c5c6ccccc6n2c5c31)C(O)=NC4	458	214
JAK1_AC	CHEMBL4062471	NC(=O)c1cnn2cccc2c1NCc1cccc(NC(=O)Cn2sc3ccccc3c2=O)c1	196	218
JAK1_AC	CHEMBL4095596	CC(CCCCCC(=O)NO)n1cc(-c2ncnc3[nH]ccc23)cn1	11	223
JAK1_AC	CHEMBL4103698	CN(c1ncnc2[nH]ccc12)[1]1C[1](NS(=O)(=O)c2ccc(F)cc2)C1	3	225
JAK1_AC	CHEMBL4113249	C[1](O)(c1ccc(Nc2nn([1]3COCC[1]3C#N)cc2C(N)=O)cc1)C(F)F	0.03	226

JAK1_AC	CHEMBL4116008	CCS(=O)(=O)N1CCN(c2ccc(Nc3ncc(C(N)=O)c(NC4CC4)n3)cc2)CC1	12	227
JAK1_AC	CHEMBL4160840	O=C(NO)c1ccc(-c2ccnc(Nc3ccc(N4CCOCC4)cc3)n2)cc1	69	228
JAK1_AC	CHEMBL4173676	CN1CCN(c2cc(Nc3nccc(Nc4ccc(F)c(Cl)c4F)n3)cc(S(C)(=O)=O)c2)CC1	3	229
JAK1_AC	CHEMBL4225016	CN(c1ncnc2[nH]ccc12)[1]1CCN(S(=O)(=O)c2cccc([N+](=O)[O-])c2)C1	459	230
JAK1_AC	CHEMBL4225595	N#Cc1cccc(S(=O)(=O)N2CC[1](Nc3ncnc4[nH]ccc34)C2)c1	510	230
JAK1_AC	CHEMBL4238926	NC(=O)c1cnc2[nH]ccc2c1NC1C2CC3CC1CC(O)(C3)C2	4	234
JAK1_AC	CHEMBL4286867	O=C(CCCCCCC(=O)Nc1ccc(-c2ccnc(Nc3ccc(N4CCOCC4)cc3)n2)cc1)NO	52.1	240
JAK1_AC	CHEMBL4561663	NC(=O)c1ccccc1Nc1cc(Nc2ccc(F)cn2)ncc1C(N)=O	26	242
JAK1_AC	CHEMBL4297507	C[1]1CN(C(=O)CC#N)[1]12CCN(c1ncnc3[nH]ccc13)C2	45871	245
JAK1_AC	CHEMBL4435170	[2H]C([2H])([2H])NC(=O)c1nnc(NC(=O)C2CC2)cc1Nc1cccc(-c2ncn(C)n2)c1OC	100.	247
JAK1_AC	CHEMBL4446102	NC(=O)c1cnc2[nH]ccc2c1NC1C2CC3CC1CC(C2)C3O	4	250
JAK1_AC	CHEMBL4448494	Cc1nc2c(F)cc(-c3nc(Nc4ccc5c(n4)CCN(CCN(C)C)C5)ncc3F)cc2n1C(C)C	787	252
JAK1_AC	CHEMBL4453646	Clc1cccc(Nc2nc(Nc3cn[nH]c3)ncc2Cl)c1	457	254
JAK1_AC	CHEMBL4469844	C[1](c1ncc(F)cn1)n1c(=O)[nH]c2cnc(-c3cnc4ccc(F)cn34)nc21	780	255
JAK1_AC	CHEMBL4466696	Cn1cc(-c2cc3nccnc3c(NC[1]3CNCCO3)n2)cn1	501.19	258
JAK1_AC	CHEMBL4563534	N#CCCNC(=O)Cc1nc2cnc3[nH]ccc3c2n1[1]1CC[1](CC#N)CC1	0.14	259
JAK1_AC	CHEMBL4476770	NC(=O)c1nc(-c2c(F)cccc2Cl)oc1Nc1ccc(C(=O)N2CCOCC2)cc1	23	262
JAK1_AC	CHEMBL4539949	CCc1cc(O)c(F)cc1-c1cc(NS(C)(=O)=O)c2cn[nH]c2c1	398	263
JAK1_AC	CHEMBL4520790	Cn1cc(Nc2nccc(N3C[1]4CC[1](C3)N4C(=O)NCC#N)n2)cn1	13	264
JAK1_AC	CHEMBL4564126	Cc1cnc(-c2cc(Cl)ccc2F)cc1-n1c(=O)n(CC(N)=O)c2cnccc21	20	267
JAK1_AC	CHEMBL4635876	OCCn1cc(Nc2ncc(Cl)c(NCc3cc(F)ccc3F)n2)cn1	0.1585	276

JAK1_AC	CHEMBL4641006	N#C[1]1(C2CC2)C(=O)N2C[1]1COCC(=O)NCCCc1cccc(n1)Nc1cc2ccn1	130	278
JAK1_AC	CHEMBL4641716	N#CC1CN(C(=O)c2ccc(-c3cccc4nc(NC(=O)C5CC5)nn34)cc2)C1	61	279
JAK1_AC	CHEMBL4645565	CC(O)(C#Cc1ccc(N2CCOCC2)c(Nc2ncnc3[nH]ccc23)c1)c1nccs1	182.6	280
JAK1_AC	CHEMBL4740298	Cn1cc(-c2cn3nccc3c(-c3cnn([1]4(CC#N)C[1](C#N)C4)c3)n2)cn1	21	282
JAK1_AC	CHEMBL4744172	O=C(Nc1c[nH]nc1-c1cc(Cl)ccc1OC(F)F)c1cnn2cccnc12	456	283
JAK1_AC	CHEMBL4789273	Cc1ccc(C(=O)/C=C/C(=O)N2CCc3ccc(Nc4ncc(C)c(-c5cnn(C(C)C)c5)n4)cc3C2)cc1	24	287

1.2 inacctives JAK1

Table S2 presents the training set of inacctives (IAs) Janus kinase inhibitors from the literature for Janus kinase 1, ChEMBLl ID, SMILES code, and IC50 values

Target	Compound	SMILES	Values	Ref
JAK1_IA	CHEMBL1446198	CCC(=O)Nc1cccc(-c2cnc3ccccc3n2)c1	50000	85
JAK1_IA	CHEMBL1537091	CNC(=S)Nc1cccc(-c2cnc3ccccc3n2)c1	50000	85
JAK1_IA	CHEMBL1553519	CS(=O)(=O)Nc1cccc(-c2cnc3ccccc3n2)c1	50000	85
JAK1_IA	CHEMBL1572928	O=C(Nc1cccc(-c2cnc3ccccc3n2)c1)c1ccco1	50000	85
JAK1_IA	CHEMBL3645535	CN(C)CC(=O)Nc1cccc(-c2cnc3ccccc3n2)c1	50000	85
JAK1_IA	CHEMBL3645538	O=C(CCCCl)Nc1cccc(-c2cnc3ccccc3n2)c1	50000	85
JAK1_IA	CHEMBL3645539	O=C(Cn1cncn1)Nc1cccc(-c2cnc3ccccc3n2)c1	50000	85
JAK1_IA	CHEMBL3645542	COc1ccc(NC(=O)Nc2cccc(-c3cnc4ccccc4n3)c2)cc1	50000	85
JAK1_IA	CHEMBL3645543	O=S(=O)(Nc1cccc(-c2cnc3ccccc3n2)c1)c1cccs1	50000	85
JAK1_IA	CHEMBL3645544	O=C(Nc1cccc(-c2cnc3ccccc3n2)c1)C1CC1	50000	85

JAK1_IA	CHEMBL3645546	CN1CCN(CC(=O)Nc2cccc(-c3cnc4ccccc4n3)c2)CC1	50000	85
JAK1_IA	CHEMBL3645548	O=C(CCCl)Nc1cccc(-c2cnc3ccccc3n2)c1	50000	85
JAK1_IA	CHEMBL3645549	O=C(CN1CCOCC1)Nc1cccc(-c2cnc3ccccc3n2)c1	50000	85
JAK1_IA	CHEMBL3645550	CC(Cl)C(=O)Nc1cccc(-c2cnc3ccccc3n2)c1	50000	85
JAK1_IA	CHEMBL278041	Oc1ccc(-c2nc(-c3ccc(F)cc3)c(-c3ccncc3)[nH]2)cc1	100000	140
JAK1_IA	CHEMBL3120960	Cn1c(-c2c[nH]nn2)nc(-c2ccc(F)cc2)c1-c1ccncc1	100000	140
JAK1_IA	CHEMBL3120963	Cn1c(-c2cn(CCO)nn2)nc(-c2ccc(F)cc2)c1-c1ccncc1	100000	140
JAK1_IA	CHEMBL3120969	CCOP(=O)(CCn1cc(-c2nc(-c3ccc(F)cc3)c(-c3ccncc3)n2C)nn1)OCC	100000	140
JAK1_IA	CHEMBL3120970	C[1](Nc1nccc(-c2c(-c3ccc(F)cc3)nc3nc(N)ccn23)n1)c1ccccc1	100000	140
JAK1_IA	CHEMBL3120983	Cn1c(-c2cn(-c3cccc(C(=O)O)c3)nn2)nc(-c2ccc(F)cc2)c1-c1ccncc1	100000	140
JAK1_IA	CHEMBL3120985	Cn1c(-c2cn(CCP(=O)([O-])[O-])nn2)nc(-c2ccc(F)cc2)c1-c1ccncc1.[Na+].[Na+]	100000	140
JAK1_IA	CHEMBL3120986	Cl.Cn1c(-c2cn([1]3CCNC[1]3F)nn2)nc(-c2ccc(F)cc2)c1-c1ccncc1	100000	140
JAK1_IA	CHEMBL3120995	Cn1c(-c2cn(-c3ccc(C(=O)O)cc3)nn2)nc(-c2ccc(F)cc2)c1-c1ccncc1	100000	140
JAK1_IA	CHEMBL3314270	Cn1c(-c2cn([1]3[1](O)[1](O)[1](CO)O[1]3O)nn2)nc(-c2ccc(F)cc2)c1-c1ccncc1	100000	140
JAK1_IA	CHEMBL3314271	CSc1nccc(-c2c(-c3ccc(F)cc3)nc3nc(N)ccn23)n1	100000	140
JAK1_IA	CHEMBL3314272	C#Cc1ccn2c(-c3ccnc(SC)n3)c(-c3ccc(F)cc3)nc2n1	100000	140
JAK1_IA	CHEMBL3314273	CSc1nccc(-c2c(-c3ccc(F)cc3)nc3nc(-c4cn(CC(=O)[O-])nn4)ccn23)n1.[Na+]	100000	140
JAK1_IA	CHEMBL3314274	CCOP(=O)(CCn1cc(-c2ccn3c(-c4ccnc(SC)n4)c(-c4ccc(F)cc4)nc3n2)nn1)OCC	100000	140
JAK1_IA	CHEMBL3314275	CSc1nccc(-c2c(-c3ccc(F)cc3)nc3nc(-c4cn(CCP(=O)([O-])[O-])nn4)ccn23)n1.[Na+].[Na+]	100000	140
JAK1_IA	CHEMBL3314278	CCOC(=O)Cn1cc(-c2ccc(-c3nc(-c4ccc(F)cc4)c(-c4cc[nH]c(=O)c4)[nH]3)cc2)nn1	80100	140
JAK1_IA	CHEMBL3314280	O=c1cc(-c2[nH]c(-c3ccc(-c4cn(CCP(=O)([O-])[O-])nn4)cc3)nc2-c2ccc(F)cc2)cc[nH]1.[Na+].[Na+]	100000	140
JAK1_IA	CHEMBL3314281	O=c1cc(-c2[nH]c(-c3ccc(O)cc3)nc2-c2ccc(F)cc2)cc[nH]1	54600	140

JAK1_IA	CHEMBL3314282	O=C([O-])c1cc(-c2nc(-c3ccc(F)cc3)c(-c3cc[nH]c(=O)c3)[nH]2)ccc1O.[Na+]	100000	140
JAK1_IA	CHEMBL3314285	CSc1nccc(-c2[nH]c(-c3ccc(O)cc3)nc2-c2ccc(F)cc2)n1	100000	140
JAK1_IA	CHEMBL3314286	C#Cc1ccc(-c2nc(-c3ccc(F)cc3)c(-c3ccnc(SC)n3)[nH]2)cc1	100000	140
JAK1_IA	CHEMBL3731087	Cc1cc(O)nc2c1c(C)nn2C	900000	187
JAK1_IA	CHEMBL4635464	Cc1[nH]nc2[nH]c(=O)cc(-c3ccccc3)c12	50000	187
JAK1_IA	CHEMBL4636368	Cc1[nH]nc2[nH]c(=O)cc([1]3CC[1](N)CC3)c12	40000	187
JAK1_IA	CHEMBL4638380	O=c1cc(C(F)F)c2c[nH]nc2[nH]1	100000	187
JAK1_IA	CHEMBL4638953	Cc1[nH]nc2[nH]c(=O)cc([1]3CC[1](NC(=O)[1]4CC4(F)F)CC3)c12	40000	187
JAK1_IA	CHEMBL4645113	O=c1cc(C2CCCCC2)c2c[nH]nc2[nH]1	15000	187
JAK1_IA	CHEMBL4647133	Cc1cc(=O)[nH]c2nn(C)c(C)c12	500000	187
JAK1_IA	CHEMBL4649054	Cc1[nH]nc2[nH]c(=O)cc([1]3CC[1](C(=O)NC4CC4)CC3)c12	40000	187
JAK1_IA	CHEMBL4276946	CC(=O)n1nc(C)c(-c2ccc(Cl)c(Cl)c2)c1N	100000	237
JAK1_IA	CHEMBL4289149	N#Cc1cc(Nc2cc(NC3CC3)c3ncc(C(N)=O)n3n2)cc(C(F)(F)F)c1	50000	242
JAK1_IA	CHEMBL4440718	[2H]C([2H])([2H])NC(=O)c1nnc(Nc2ccccn2)cc1Nc1ccccc1S(C)(=O)=O	40000	249
JAK1_IA	CHEMBL4526283	CNC(=O)c1cnc(Nc2ccc(F)cn2)cc1Nc1ccccc1S(C)(=O)=O	50000	249
JAK1_IA	CHEMBL4634634	C[1]1C[1]1C(=O)N1CCN(c2cnc(C#N)c(-c3cnn(C)c3)n2)C[1]1C	100000	275

1.3 actives JAK2

Table S3 presents the training set of active Janus kinase inhibitors from the literature for Janus kinase 2, including ChEMBLl ID, SMILES code, and IC50 values

Target	Compound	SMILES	Values	Ref
JAK2_AC	CHEMBL4470355	Cc1cncc(Nc2cc(NC3CCN(c4ccc(C#N)cn4)CC3)c(C(N)=O)cn2)n1	4	26

JAK2_AC	CHEMBL4549451	COc1ccc(-c2cc3c(N[1](CO)CC4CC4)c(C(N)=O)cnn3c2)cn1	25	27
JAK2_AC	CHEMBL4297507	C[1]1CN(C(=O)CC#N)[1]12CCN(c1ncnc3[nH]ccc13)C2	458	28
JAK2_AC	CHEMBL4303389	C[1](Nc1cc(-c2cnn(C)c2)cc(Nc2cnccn2)n1)c1ccc(F)cc1	0.27	28
JAK2_AC	CHEMBL1287853	Cc1cnc(Nc2ccc(OCCN3CCCC3)cc2)nc1Nc1cccc(S(=O)(=O)NC(C)(C)C)c1	0.75	29
JAK2_AC	CHEMBL2105759	CCS(=O)(=O)N1CC(CC#N)(n2cc(-c3ncnc4[nH]ccc34)cn2)C1	0.29	29
JAK2_AC	CHEMBL4743499	N#CC[1](C1CCCC1)n1cc(-c2nc(Nc3ccc(C4CCNCC4)cc3)nc3[nH]ccc23)cn1	0.099	29
JAK2_AC	CHEMBL4634073	COC(=O)Nc1ccc(-c2c(OC3CCN(C)CC3)nc3c(-c4ccc(OC)c(OC)c4)cnn3c2N)cc1	84	31
JAK2_AC	CHEMBL4590390	Cn1nc(-c2nn(C3CCCN(C(=O)CC#N)C3)c3ncnc(N)c23)cc1C(C)(C)O	8	32
JAK2_AC	CHEMBL4568087	Cn1cc(-c2cnc3c(-c4csc(C(=O)N[1]5CCCC[1]5N)c4)cnn3c2)cn1	64	33
JAK2_AC	CHEMBL4634591	C[1](Oc1cc(-c2cnn(C3CCOCC3)c2)cnc1N)c1cc(F)ccc1Cl	3	34
JAK2_AC	CHEMBL3601120	C=CC(=O)Nc1cccc(CNc2nc(Nc3cnn(CCO)c3)ncc2Cl)c1	32	35
JAK2_AC	CHEMBL4644578	C[1]1[1]2CN(c3nc(Nc4cnn(CCO)c4)ncc3F)C[1]12NC(=O)[1]1C[1]1C#N	128	37
JAK2_AC	CHEMBL3426891	CN1CCN(c2ccc(Nc3ncc(Cl)c(NC[1]4CCCO4)n3)cc2)CC1	6	38
JAK2_AC	CHEMBL3360356	O=C(Nc1nc2cccc(-c3ccc(CN4CCC(F)(F)CC4)cc3)n2n1)C1CC1	15.51	39
JAK2_AC	CHEMBL4089118	N#C[1]1CCOC[1]1n1cc(C(N)=O)c(Nc2ccc([1](O)C(F)(F)F)cc2)n1	0.47	66
JAK2_AC	CHEMBL2376439	CC(=O)N[1]1CCc2ccc(Oc3cnc4[nH]cc(C(=O)N[1](C(=O)N5CC(C#N)C5)C5CC5)c4n3)cc21	39	68
JAK2_AC	CHEMBL1923571	COc1ccccc1-c1ccc2cnc(Nc3ccc(C(=O)N4CCOCC4)cc3)nn12	0.17	71
JAK2_AC	CHEMBL1078889	CS(=O)(=O)Nc1ccc(-c2ccnc(Nc3ccc(CN4CCS(=O)(=O)CC4)cc3)n2)cc1	2	74
JAK2_AC	CHEMBL1079594	N#Cc1ccc2ncn(-c3ncc4[nH]c(=O)n([1]5CCOc6c(F)cccc65)c4n3)c2c1	5	75
JAK2_AC	CHEMBL1081290	COc1cc(Nc2nc3cccc(-c4ccccc4)c3o2)cc(OC)c1OC	15	76
JAK2_AC	CHEMBL1081484	COc1cc(Nc2nc3cccc(-c4ccc(C(=O)N5CCOCC5)c(F)c4)c3o2)cc(OC)c1OC	3	76
JAK2_AC	CHEMBL1089815	Cc1cc(-c2cccc3ncc(-c4ccc(C(=O)N5CCOCC5)c(C)c4)nc23)ccc1CN1CCOCC1	13	77

JAK2_AC	CHEMBL1171837	Cc1ccc(C(=O)Nc2ccc(CN3CCN(C)CC3)c(C(F)(F)F)c2)cc1C#Cc1cnc2cccnn12	169	79
JAK2_AC	CHEMBL120563	CN(C)C(=S)SSC(=S)N(C)C	223	80
JAK2_AC	CHEMBL1231124	Cc1cc(Nc2nc(N[1](C)c3ncc(F)cn3)ncc2Cl)[nH]n1	0.4	81
JAK2_AC	CHEMBL4564280	CN(C)C(=O)/C(C#N)=C/c1ccc(-c2nc3cnc4[nH]ccc4c3n2C2CCCCC2)o1	343	81
JAK2_AC	CHEMBL1270399	Cc1cn2c(-c3cn[nH]c3)cnc2c(Nc2cc([1](C)N3C[1](C)O[1](C)C3)ns2)n1	10	82
JAK2_AC	CHEMBL1289097	CC(C)[1](Nc1nc2cc[nH]c(=O)c2c2cc(F)ccc12)C(F)(F)F	1	83
JAK2_AC	CHEMBL1630791	C[1]1CCN(C(=O)CO)C[1]1N(C)c1ncnc2[nH]ccc12	480	87
JAK2_AC	CHEMBL1650724	C[1](Nc1ncc(Cl)c(Nc2cc(C3CC3)[nH]n2)n1)c1ncc(F)cn1	3	88
JAK2_AC	CHEMBL1682386	Cc1cc(Nc2nc(N3CCN(C(=O)Cc4ccc(Cl)cc4)CC3)nn3cccc23)n[nH]1	0.6	90
JAK2_AC	CHEMBL2062807	CS(=O)(=O)c1ccc(-c2cccn3nc(Nc4cccc(CN5CCS(=O)(=O)CC5)c4)nc23)cc1	0.64	11
JAK2_AC	CHEMBL1774056	Cc1cc(Nc2nc(N[1](C)c3ccc(F)cn3)c(C#N)cc2F)n[nH]1	1	93
JAK2_AC	CHEMBL1784637	CNC(=O)c1ncc(C#Cc2cc(C(=O)Nc3ccc(CN4CCN(CCO)CC4)c(C(F)(F)F)c3)ccc2C)n1C	536	94
JAK2_AC	CHEMBL1789941	N#CC[1](C1CCCC1)n1cc(-c2ncnc3[nH]ccc23)cn1	0.036	96
JAK2_AC	CHEMBL1835153	NC(=O)c1cnc(N[1](C2CC2)C(F)(F)F)c2c1[nH]c1cc(-c3cnc(N)nc3)ccc12	0.4	97
JAK2_AC	CHEMBL191003	Nc1nc(Nc2ccc(S(N)(=O)=O)cc2)nn1C(=O)c1c(F)cccc1F	1	100
JAK2_AC	CHEMBL1933343	Cc1cnc2nc1-c1cccc(c1)OCC/C=C/CN(C)Cc1cccc(c1)N2	180	101
JAK2_AC	CHEMBL1934340	CN(c1cccc(-c2ccc3cnc(Nc4ccc(C5CCN(CC(N)=O)CC5)cc4)nn23)c1)S(C)(=O)=O	0.09	102
JAK2_AC	CHEMBL1938654	CNC(=O)c1cnc(N)c2cc(-c3ccc(S(=O)(=O)NC(C)(C)C)cc3)sc12	1	103
JAK2_AC	CHEMBL1944698	CN1C/C=C/CCOc2cccc(c2)-c2ccnc(n2)Nc2cccc(c2)C1	73	104
JAK2_AC	CHEMBL1946150	CCS(=O)(=O)CNc1cc2cc(c1)Nc1nccc(n1)-c1cccc(c1)OCC/C=C/CN(C)C2	18	105
JAK2_AC	CHEMBL2035053	Cc1cnc2nc1-c1ccnc(c1)OCC/C=C/COCc1cc(ccc1OCCN1CCCC1)N2	14	105
JAK2_AC	CHEMBL1983268	CN1CCN(c2ccc(C(=O)Nc3n[nH]c4ccc(Cc5cc(F)cc(F)c5)cc34)c(NC3CCOCC3)c2)CC1	40	106

JAK2_AC	CHEMBL1995703	Cc1cnc(Nc2ccc(N3CCN(C)CC3)cc2)nc1Nc1cccc(S(=O)(=O)NC(C)(C)C)c1	6	107
JAK2_AC	CHEMBL2006765	CCCNC(=O)c1ccc(Nc2nc(NCC(F)(F)F)c3cc[nH]c3n2)cc1	57	108
JAK2_AC	CHEMBL2012881	O=c1[nH]nc2cc(CO)c3ccc(-c4ccc[nH]4)cc3n12	650	109
JAK2_AC	CHEMBL2035187	C1=N/C2=N/c3ccc(OCCN4CCCC4)c(c3)COC/C=C/COCc3cccc(c3)C(=C1)N2	1	110
JAK2_AC	CHEMBL3961053	O=C(NO)C1CCN(CCOc2ccc3cc2COC/C=C/COCc2cccc(c2)-c2ccnc(n2)N3)CC1	0.97	110
JAK2_AC	CHEMBL2103874	CNS(=O)(=O)C[1]1CC[1](N(C)c2[nH]cnc3nccc2-3)CC1	457	112
JAK2_AC	CHEMBL3039513	CC[1](C)(Nc1ccnc(-c2c[nH]c3ncccc23)n1)C(=O)NCC(F)(F)F	13	114
JAK2_AC	CHEMBL3301607	O=C(Nc1nc2cccc(-c3ccc(CN4CCS(=O)(=O)CC4)cc3)n2n1)C1CC1	25	114
JAK2_AC	CHEMBL21156	CC(C)(C)c1nc2c3ccc(F)cc3c3c(=O)[nH]ccc3c2[nH]1	1	116
JAK2_AC	CHEMBL2151321	CC(C)Oc1cc(-n2cnc3ccc(N[1](C)c4ccc(F)cn4)nc32)n[nH]1	68.3	118
JAK2_AC	CHEMBL2170414	NC(=O)c1ccc(Nc2ncc(F)c(Nc3ccccc3Cl)n2)cc1	33.4	119
JAK2_AC	CHEMBL2170419	O=C(O)c1ccc(Nc2ncc(F)c(Nc3ccccc3Cl)n2)cc1	91	120
JAK2_AC	CHEMBL2178800	Cc1ccc(Cl)cc1-c1nn(C)cc1NC(=O)c1cnn2cccnc12	34.5	121
JAK2_AC	CHEMBL2181088	Cc1cnc2nc1-c1cccc(c1)COCC=CCOCc1cc(ccc1OCCN1CCCC1)N2	7	122
JAK2_AC	CHEMBL2208035	O=C1CC[1](C(=O)Nc2ccc(-c3ccnc(Nc4ccc(N5CCOCC5)cc4)n3)cc2)N1	0.9	123
JAK2_AC	CHEMBL221029	C[1](Nc1ccnc(-n2cnc3ccncc32)n1)c1ccccc1	4	124
JAK2_AC	CHEMBL221959	C[1]1CCN(C(=O)CC#N)C[1]1N(C)c1ncnc2[nH]ccc12	0.5	126
JAK2_AC	CHEMBL4291720	COc1cc2c(=O)n(C3CCCCCC3)c3c4cc[nH]c4ncc3c2cc1OC	0.89	127
JAK2_AC	CHEMBL2322135	c1cc2c(ncc3[nH]nc(C4CCCCC4)c32)[nH]1	10	129
JAK2_AC	CHEMBL2325897	N#C[1]1CCN(C(=O)[1](NC(=O)c2c[nH]c3ncc(C4CC4)nc23)C2CC2)C1	0.3	130
JAK2_AC	CHEMBL2381975	Cc1cc(Nc2nc(N[1](C)c3ccc(F)cn3)c(F)c(N3CCN(S(C)(=O)=O)CC3)n2)n[nH]1	3	133
JAK2_AC	CHEMBL2403108	Cc1cc(Nc2ncc(Cl)c(Nc3ccccc3S(=O)(=O)C(C)C)n2)c(OC(C)C)cc1C1CCNCC1	610	134

JAK2_AC	CHEMBL2414541	O=C(O)c1ccc(Nc2nc3c(-c4cnn(C5CCCCC5)c4)cccn3n2)cc1	31	136
JAK2_AC	CHEMBL2443044	CN1CCC(NC(=O)c2cc(-c3c(O)cc(O)cc3Oc3ccc([N+](=O)[O-])cc3)on2)CC1	10	137
JAK2_AC	CHEMBL272424	O=c1[nH]ccc2c3[nH]c(-c4c(F)cccc4Cl)nc3c3ccc(F)cc3c12	0.2	139
JAK2_AC	CHEMBL302449	Cn1c2ccccc2c2c3c(c4c5ccccc5n(CCC#N)c4c21)CNC3=O	67.2	141
JAK2_AC	CHEMBL3115325	COc1ccc(Nc2nc(N3CCOCC3)c3nc[nH]c3n2)cc1OC	110	142
JAK2_AC	CHEMBL3116050	C[1](Nc1nc(Nc2cn(C)cn2)c2cc[nH]c2n1)c1ncc(F)cn1	2	143
JAK2_AC	CHEMBL3126350	NS(=O)(=O)c1cc(Cl)c(Nc2nc3ccncc3c3c(=O)[nH]ccc23)c(Cl)c1	0.15	144
JAK2_AC	CHEMBL3128069	Cc1nc([1](C)(O)CO)sc1-c1cnc(N)c(O[1](C)c2cc(F)ccc2-n2nccn2)c1	12	145
JAK2_AC	CHEMBL4116008	CCS(=O)(=O)N1CCN(c2ccc(Nc3ncc(C(N)=O)c(NC4CC4)n3)cc2)CC1	6	146
JAK2_AC	CHEMBL603469	C[1]12O[1](C[1]1(O)CO)n1c3ccccc3c3c4c(c5c6ccccc6n2c5c31)CNC4=O	0.9	146
JAK2_AC	CHEMBL4874046	CNC(=O)c1ccc(Nc2ncc(C(F)(F)F)c(Nc3ccccc3OC)n2)cc1	17.89	147
JAK2_AC	CHEMBL3234884	CS(=O)(=O)c1ccc(Nc2sc(-c3ccccc3)cc2C(N)=O)nc1	16	148
JAK2_AC	CHEMBL3298194	COc1cc(Nc2nc3cccc(-c4cccc5[nH]cnc45)c3o2)cc(OC)c1OC	460	149
JAK2_AC	CHEMBL4777200	CNC(=O)c1cnc2[nH]ccc2c1N[1]1CCN(Cc2cccc(Cl)c2)C[1]1F	28	150
JAK2_AC	CHEMBL3301625	C=CC(=O)Nc1cccc(Nc2nc(Nc3ccc(OCCOC)cc3)ncc2F)c1	533.7	151
JAK2_AC	CHEMBL3330130	CCc1ccc(C(F)(F)F)cc1-c1[nH]c(-c2ccnc(N)n2)cc1C(N)=O	2	152
JAK2_AC	CHEMBL3352835	COC1CCN(c2nccc(Nc3cc(NC(=O)c4c(Cl)cccc4Cl)ccn3)n2)CC1	44	153
JAK2_AC	CHEMBL3359908	CC1(C)CCC[1]1Nc1c(C(N)=O)cnn2cccc12	16	155
JAK2_AC	CHEMBL3393341	C[1]1CCCN(c2ncnc3[nH]cc(-c4cccc(C#N)c4)c23)C1	575	156
JAK2_AC	CHEMBL3403545	CCc1ccc(Cl)cc1-n1cc(-c2ncnc3[nH]ccc23)cc1C(N)=O	1	157
JAK2_AC	CHEMBL3622143	CCn1c(C(=O)N(C2CC2)C2CC2)cc2c3c(ncn3C)c(Nc3nc(C)c(C(=O)NC)s3)nc21	0.9	158

JAK2_AC	CHEMBL3589176	COc1ccc(-c2cc(C(N)=O)c3[nH]c4cc(C(=O)N5CCOCC5)ccc4c3c2)cc1F	457	159
JAK2_AC	CHEMBL3856117	O=c1[nH]c2cnc3[nH]ccc3c2n1C1CCCCCC1	23	160
JAK2_AC	CHEMBL3593776	N#Cc1ccc(N2CCC(Nc3c(C(N)=O)cnc4[nH]ccc34)CC2)nn1	12	161
JAK2_AC	CHEMBL3622196	COc1ccc(-c2cc(C(N)=O)c3[nH]c4ccc(C(=O)N5CCOCC5)cc4c3c2)cc1	2	163
JAK2_AC	CHEMBL3622127	CNc1nc2c(cc(C(=O)N(C3CC3)C3CC3)n2C[1](O)CO)c2c1ncn2C	456	164
JAK2_AC	CHEMBL3685796	Cc1cc(Nc2nccc(C)n2)cc(-c2cnc([1]3(O)CC[1](C(=O)O)C(C)(C)C3)s2)c1	242	166
JAK2_AC	CHEMBL3622821	CC[1]1CN(C(=O)NCC(F)(F)F)C[1]1c1cnc2cnc3[nH]ccc3n12	458	166
JAK2_AC	CHEMBL3642343	CN(c1ccc(-c2cccn3nc(Nc4ccc(CN5CCS(=O)(=O)CC5)cc4)nc23)cc1)S(C)(=O)=O	0.27	168
JAK2_AC	CHEMBL3644618	CCc1cc(O)c(F)cc1-c1ccc2c(-c3nc4c([nH]3)CCN(C(=O)c3ccc(F)cc3)C4)n[nH]c2c1	0.3	169
JAK2_AC	CHEMBL3884848	Cc1cccc(Nc2ccnc(Nc3ccc(N4CCN(C)CC4)c(C)c3)n2)c1	50	170
JAK2_AC	CHEMBL4279720	C[1]1(NC#N)CCc2ccc(-c3ncnc4[nH]ccc34)cc21	71	170
JAK2_AC	CHEMBL3649628	Cc1ccc(-c2cc(C(N)=O)c3[nH]c4ccc(C(=O)O)cc4c3c2)c(F)c1	1	172
JAK2_AC	CHEMBL3650980	CCOC(=O)c1c[nH]c2ncnc(-c3ccc(N)cc3)c12	53	173
JAK2_AC	CHEMBL4209458	C=CC(=O)Nc1ccc2ncn(-c3cncc(NC(C)(C)C)n3)c2c1	40	174
JAK2_AC	CHEMBL3652404	C[1]1CN(S(=O)(=O)C[1]2CC[1](N(C)c3ncnc4[nH]ccc34)CC2)C[1]1CO	0.0165	175
JAK2_AC	CHEMBL3655103	CN(c1ncnc2[nH]ccc12)[1]1C[1](NS(=O)(=O)N2CCC(c3cc[nH]n3)CC2)C1	52	176
JAK2_AC	CHEMBL3658108	Cc1ccc(NC(=O)c2cccc(C(F)(F)F)c2)cc1N1CCc2nc(Nc3ccc(C(=O)N4CCN(C)CC4)nc3)ncc2C1	3	177
JAK2_AC	CHEMBL3659504	Cc1cc(-n2cnc3ccc(N[1](C)c4ccc(F)cn4)nc32)n[nH]1	3	178
JAK2_AC	CHEMBL3665151	CS(=O)(=O)N1CCCC(Nc2ncccc2-c2cnc3[nH]ccc3n2)CC1	0.0089	179
JAK2_AC	CHEMBL3671804	CC1(C)[1](Nc2c(C(N)=O)cnn3cc(-c4cccnc4)cc23)CC[1]1(C)N	11	181

JAK2_AC	CHEMBL3675912	O=C(NC1CCC(O)CC1)c1cnn2ccc(N3CCCC3c3cc(F)ccc3CO)nc12	42.3	183
JAK2_AC	CHEMBL3689514	CC1CN(C(=O)CC#N)C12CCCN(c1ncnc3[nH]ccc13)C2	1	184
JAK2_AC	CHEMBL3694592	Clc1cnc2nc1NCc1cc(Br)cc(c1)OCCCc1cccc(c1)N2	5	185
JAK2_AC	CHEMBL3699558	CC(C)(C(=O)NCC(F)(F)F)c1ccc(Nc2nn([1]3CCCC[1]3C#N)cc2C(N)=O)cc1	0.66	186
JAK2_AC	CHEMBL4643061	Cc1[nH]nc2[nH]c(=O)cc([1]3C[1]4C[1]3C[1]4CC#N)c12	12	187
JAK2_AC	CHEMBL3798482	Cn1c(C(=O)NCCCN2CCOCC2)cc2c(-c3ccc(NC(=O)C(C)(C)C)cc3)ncnc21	6	190
JAK2_AC	CHEMBL386637	CN(C)[1]1CCCN(c2ccc(C(F)(F)F)cc2NC(=O)c2cc(C#Cc3cnc(N)nc3)ccc2F)C1	550	192
JAK2_AC	CHEMBL386760	COc1ccc(N(C(=O)Oc2c(C)cccc2C)c2ccnc(Nc3cc(OC)c(OCCCN4CCN(C)CC4)c(OC)c3)n2)c(OC)c1	240	193
JAK2_AC	CHEMBL388978	CN[1]1C[1]2O[1](C)([1]1OC)n1c3ccccc3c3c4c(c5c6ccccc6n2c5c31)C(=O)NC4	0.115	195
JAK2_AC	CHEMBL4238357	Cn1cc(-c2nn(CC3CCCN(C(=O)CC#N)C3)c3ncnc(N)c23)cn1	78.4	195
JAK2_AC	CHEMBL4238926	NC(=O)c1cnc2[nH]ccc2c1NC1C2CC3CC1CC(O)(C3)C2	458	195
JAK2_AC	CHEMBL3940537	Cc1cnc(Nc2ccc(N3CCOCC3)cc2)nc1-c1cc2c(o1)CCN(S(C)(=O)=O)C2	0.7	198
JAK2_AC	CHEMBL3896019	Cc1c(-c2c(F)cc(C(N)=O)c3[nH]c4cc(C(C)(C)O)ccc4c23)cccc1-n1c(=O)c2cccc(F)c2n(C)c1=O	170	199
JAK2_AC	CHEMBL3918580	Cc1c(-c2ccc(C(N)=O)c3[nH]c4cc(C(C)(C)O)ccc4c23)cccc1-n1cnc2ccccc2c1=O	240	199
JAK2_AC	CHEMBL3954894	ClC[1]1CC[1](c2nnn3cnc4[nH]ccc4c23)CC1	1	200
JAK2_AC	CHEMBL3911320	Nc1n[nH]c2ccc(C(=O)N3CCC[1]3c3ccc(Cl)cc3)cc12	286	202
JAK2_AC	CHEMBL391492	CONC(=O)c1cc2c(N[1](C)c3ccccc3)nc(-n3cnc4ccncc43)nc2n1C	6	203
JAK2_AC	CHEMBL3925083	CN(c1ncnc2[nH]ccc12)[1]1CC[1](CS(=O)(=O)NC2CCC2)CC1	101	204
JAK2_AC	CHEMBL3935288	N#CCC1(n2cc(-c3ncnc4[nH]ccc34)cn2)CN(C2CCN(C(=O)c3ccc(-c4cccc5ncccc45)c(F)c3)CC2)C1	10	206
JAK2_AC	CHEMBL3984749	CN1CN([1]2CC[1](CN3CCCS3(=O)=O)CC2)c2c(cnc3[nH]ccc23)C1=O	0.62	207
JAK2_AC	CHEMBL3944875	Cn1cc(Nc2ncc(Cl)c(NC3COC4C(NS(C)(=O)=O)COC34)n2)cn1	51	208

JAK2_AC	CHEMBL3943922	Cn1cc(Nc2ncc(Cl)c(NC3CC4CN(c5ccc(C#N)cn5)CC4C3)n2)cn1	3	208
JAK2_AC	CHEMBL3953929	CCCCCC(CC#N)n1cc(-c2ncnc3[nH]ccc23)cn1	0.26	211
JAK2_AC	CHEMBL4113706	CN(c1ccc(S(N)(=O)=O)cn1)[1]1CCN(c2ncnc3[nH]ccc23)C1	41.1	212
JAK2_AC	CHEMBL3972665	CC(F)(F)C(=O)N1CC[1](CC#N)(n2cc(C(N)=O)c(Nc3ccc(F)nc3)n2)[1](F)C1	0.1	213
JAK2_AC	CHEMBL3975634	CN[1]1CC2OC([1]1OC)n1c3ccccc3c3c4c(c5c6ccccc6n2c5c31)C(O)=NC4	0.55	214
JAK2_AC	CHEMBL3986824	Nc1cncc(-c2cn3ccnc3c(Nc3ccc(N4CCN(C5COC5)CC4)cc3)n2)n1	120	216
JAK2_AC	CHEMBL4060528	COC[1](C(=O)Nc1cccc2c(-c3nc(Nc4cn(C)nc4C)ncc3F)c[nH]c12)N1CCN(C)CC1	322	217
JAK2_AC	CHEMBL4072239	NC(=O)c1cnc(Nc2ccccc2)cc1NCc1ccccc1	203	218
JAK2_AC	CHEMBL4113249	C[1](O)(c1ccc(Nc2nn([1]3COCC[1]3C#N)cc2C(N)=O)cc1)C(F)F	0.2	226
JAK2_AC	CHEMBL4175964	CS(=O)(=O)Nc1ccc(-c2ccnc(Nc3ccc(N4CCC(C(=O)NCCCC(=O)NO)CC4)cc3)n2)cc1	1	228
JAK2_AC	CHEMBL4165758	Cc1cnc(Nc2cc(N3CCOCC3)cc(N3CCOCC3)c2)nc1Nc1cccc(CO)c1	3	229
JAK2_AC	CHEMBL4225016	CN(c1ncnc2[nH]ccc12)[1]1CCN(S(=O)(=O)c2cccc([N+](=O)[O-])c2)C1	18	230
JAK2_AC	CHEMBL4227082	Cc1cn2c(-c3cn[nH]c3)cnc2c(Nc2cc(Cc3ccccn3)ns2)n1	500	231
JAK2_AC	CHEMBL4228766	C1=C/COc2ccc(o2)-c2ccnc(n2)Nc2ccc(OCCN3CCCC3)c(c2)COC/1	46	232
JAK2_AC	CHEMBL4590082	Cc1cnc(Nc2ccc(N3CCOCC3)cc2)nc1-c1cc2c(o1)CCN(C(=O)CC#N)C2	0.5	233
JAK2_AC	CHEMBL4242626	N#CCCn1cc(-c2nc(Nc3ccc(NC(=O)CCCCCCC(=O)NO)cc3)nc3[nH]ccc23)cn1	0.04	235
JAK2_AC	CHEMBL4434658	NC(=O)c1ccc(NC(=O)N2CCN(c3ccnc(Nc4ccc(N5CCOCC5)cc4)n3)CC2)cc1	11	246
JAK2_AC	CHEMBL4437903	Cc1cc(Nc2cnc(C#N)c(N[1](C)c3ccc(F)cc3)n2)n[nH]1	33	248
JAK2_AC	CHEMBL4447631	Cc1cnc(Nc2ccc(N3CCC(N(C)CCO)CC3)c(F)c2)nc1-c1cnn(C(C)C)c1	0.7	251
JAK2_AC	CHEMBL4448494	Cc1nc2c(F)cc(-c3nc(Nc4ccc5c(n4)CCN(CCN(C)C)C5)ncc3F)cc2n1C(C)C	170	252
JAK2_AC	CHEMBL4453646	Clc1cccc(Nc2nc(Nc3cn[nH]c3)ncc2Cl)c1	2	253
JAK2_AC	CHEMBL4536396	C[1](c1ccc(F)cn1)n1c(=O)[nH]c2cnc(-c3cnc4ccc(F)cn34)nc21	0.3	255

JAK2_AC	CHEMBL482189	CC(C)(C)NS(=O)(=O)c1ccc(-c2ccc3[nH]nc(N)c3c2)c(Cl)c1	39	258
JAK2_AC	CHEMBL4470339	Cc1nc2cnc3[nH]ccc3c2n1[1]1CC[1](CC#N)CC1	0.2	259
JAK2_AC	CHEMBL4475494	CC(C)(O)[1](F)CN1Cc2cc(NC(=O)c3cnn4cccnc34)c(N3CCOCC3)cc2C1=O	486	261
JAK2_AC	CHEMBL4476770	NC(=O)c1nc(-c2c(F)cccc2Cl)oc1Nc1ccc(C(=O)N2CCOCC2)cc1	26	262
JAK2_AC	CHEMBL4531121	CCS(=O)(=O)Nc1cc(-c2ccc(O)cc2)cc2[nH]ncc12	10	263
JAK2_AC	CHEMBL4520790	Cn1cc(Nc2nccc(N3C[1]4CC[1](C3)N4C(=O)NCC#N)n2)cn1	67	264
JAK2_AC	CHEMBL4564551	Cc1nn(CC2CC2)nc1-c1ncnc2[nH]ccc12	41.9	268
JAK2_AC	CHEMBL4582651	CO[1]1(C(=O)N[1](C)c2ccc(-n3cc(F)cn3)nc2)CC[1](c2nc(C)cc(Nc3cc(C)[nH]n3)n2)CC1	2	269
JAK2_AC	CHEMBL4594441	COc1cc(Nc2ncc(C)c(Nc3ccc4oc(=O)[nH]c4c3)n2)cc(C)c1F	12	271
JAK2_AC	CHEMBL4634979	COc1cc(-c2cnc3cccc(-c4cc(F)c(CN5CCS(=O)(=O)CC5)c(F)c4)c3n2)ccc1OCCCN1CCOCC1	0.2512	276
JAK2_AC	CHEMBL4641006	N#C[1]1(C2CC2)C(=O)N2C[1]1COCC(=O)NCCCc1cccc(n1)Nc1cc2ccn1	100	278
JAK2_AC	CHEMBL4644662	C=C(C#N)C(O)c1ccc(-c2cccc3nc(NC(=O)C4CC4)nn23)cc1	55	279
JAK2_AC	CHEMBL4740298	Cn1cc(-c2cn3nccc3c(-c3cnn([1]4(CC#N)C[1](C#N)C4)c3)n2)cn1	8	282
JAK2_AC	CHEMBL475251	COc1cc(Nc2ncc(F)c(Nc3ccc4c(n3)NC(=O)C(C)(C)O4)n2)cc(OC)c1OC	6	285
JAK2_AC	CHEMBL4789273	Cc1ccc(C(=O)/C=C/C(=O)N2CCc3ccc(Nc4ncc(C)c(-c5cnn(C(C)C)c5)n4)cc3C2)cc1	0.9	287
JAK2_AC	CHEMBL4786783	C#Cc1nc(Nc2cccc(CC(N)=O)c2)nc2[nH]cnc12	220	288
JAK2_AC	CHEMBL4794655	Cc1nc(Cl)cc(Nc2c[nH]nc2-c2nc(-c3ccc(CN4CCOCC4)cc3)c[nH]2)n1	8	290
JAK2_AC	CHEMBL4797515	CNc1nc(C)c(C(=O)/C=C/c2ccsc2)s1	17.64	291
JAK2_AC	CHEMBL4862283	COc1ccc2nc(N3CCC(C(CC#N)n4cc(-c5ncnc6[nH]ccc56)cn4)CC3)sc2c1	1	292
JAK2_AC	CHEMBL495727	O=C(Nc1c[nH]nc1-c1nc2ccc(CN3CCOCC3)cc2[nH]1)NC1CC1	1	296
JAK2_AC	CHEMBL5078528	COc1cc(Nc2ncc(C)c(N3CCC4(CCNC4=O)CC3)n2)cc(OC)c1OC	6	297
JAK2_AC	CHEMBL5083330	O=C(CCCCCn1cc(Nc2ncc3ccn(Cc4ccccc4F)c3n2)cn1)NO	10	298

JAK2_AC	CHEMBL5084861	COc1cc(-c2ccc(O)cc2)cc2cnc(Nc3cnn(C)c3)nc12	0.1585	299
JAK2_AC	CHEMBL5169760	Cc1nc(-c2ccccc2Nc2nc(Nc3ccc(CN4CCN(C)CC4)cc3)ncc2Cl)n[nH]1	130	300
JAK2_AC	CHEMBL5175536	CN(c1ncnc2[nH]ccc12)N1CCC[1](C(=O)NCC(F)(F)F)C1	13	301
JAK2_AC	CHEMBL5269065	CC(C)C(Nc1cnnc(-c2c[nH]c3ncc(F)cc23)n1)C(=O)NCC(C)(C)C	10	302
JAK2_AC	CHEMBL5279426	Cn1nc(C2NN(C3CCCN(C(=O)CC#N)C3)c3ncnc(N)c32)cc1C(C)(C)O	8	303
JAK2_AC	CHEMBL5281221	Cc1cnc(Nc2ccc(N3CCN(C)CC3)cc2)nc1Nc1ccc(S(=O)(=O)NC(C)(C)C)cc1	0.51	304
JAK2_AC	CHEMBL5281283	C[1]1CCN(C(=O)CC#N)C[1]1n1ccc2cnc3[nH]ccc3c21	0.3	305
JAK2_AC	CHEMBL559626	CC/C(=C(/CC)c1ccc(O)c(CN(CC)CC)c1)c1ccc(O)c(CN(CC)CC)c1	60	306
JAK2_AC	CHEMBL570002	Cc1cc(Nc2cnc(C#N)c(N[1](C)c3ncc(F)cn3)n2)n[nH]1	3	308
JAK2_AC	CHEMBL570899	Cc1cc(Nc2cncc(N[1](C)c3ncc(F)cn3)n2)n[nH]1	3	308
JAK2_AC	CHEMBL583895	C[1](Nc1cc(-c2c(N)nn3cccnc23)ncn1)c1ccc(F)cc1	0.2	309
JAK2_AC	CHEMBL589869	Oc1ccc(C2Nc3ccccc3-c3ncnc4[nH]cc2c34)cc1F	140	310

1.4 inactives JAK2

Table S4 presents the training set of inacctives (IAs) Janus kinase inhibitors from the literature for Janus kinase 2, ChEMBLl ID, SMILES code, and IC50 values

Target	Compound	SMILES	Values	Ref
JAK2_IA	CHEMBL1221633	O=C(CCc1cn(Cc2ccc(Cl)cc2)c2ccccc12)Nc1ccc([N+](=O)[O-])cc1	106700	80
JAK2_IA	CHEMBL3645543	O=S(=O)(Nc1cccc(-c2cnc3ccccc3n2)c1)c1cccs1	55500	85
JAK2_IA	CHEMBL1448	O=C(Nc1ccc([N+](=O)[O-])cc1Cl)c1cc(Cl)ccc1O	100000	86
JAK2_IA	CHEMBL185140	O=C(CCc1cn(Cc2ccc(Cl)cc2)c2ccccc12)Nc1ccncc1	133600	98

JAK2_IA	CHEMBL4476380	Nc1nc(Nc2ccc(S(N)(=O)=O)cc2)nn1C(=O)Nc1ccc(OCC(=O)O)cc1	56530	100
JAK2_IA	CHEMBL3622826	NCCn1c(C2CCNCC2)nc2cc(C(N)=O)ccc21	437300	114
JAK2_IA	CHEMBL406845	CC(C)(C)n1nc(-c2ccc(Cl)cc2)c2c(N)ncnc21	50000000	116
JAK2_IA	CHEMBL228043	C/C(O)=C(\C#N)C(=O)Nc1cc(Br)ccc1Br	500000	128
JAK2_IA	CHEMBL278041	Oc1ccc(-c2nc(-c3ccc(F)cc3)c(-c3ccncc3)[nH]2)cc1	100000	140
JAK2_IA	CHEMBL3120960	Cn1c(-c2c[nH]nn2)nc(-c2ccc(F)cc2)c1-c1ccncc1	100000	140
JAK2_IA	CHEMBL3120963	Cn1c(-c2cn(CCO)nn2)nc(-c2ccc(F)cc2)c1-c1ccncc1	100000	140
JAK2_IA	CHEMBL3120969	CCOP(=O)(CCn1cc(-c2nc(-c3ccc(F)cc3)c(-c3ccncc3)n2C)nn1)OCC	100000	140
JAK2_IA	CHEMBL3120970	C[1](Nc1nccc(-c2c(-c3ccc(F)cc3)nc3nc(N)ccn23)n1)c1ccccc1	100000	140
JAK2_IA	CHEMBL3120983	Cn1c(-c2cn(-c3cccc(C(=O)O)c3)nn2)nc(-c2ccc(F)cc2)c1-c1ccncc1	100000	140
JAK2_IA	CHEMBL3120985	Cn1c(-c2cn(CCP(=O)([O-])[O-])nn2)nc(-c2ccc(F)cc2)c1-c1ccncc1.[Na+].[Na+]	100000	140
JAK2_IA	CHEMBL3120986	Cl.Cn1c(-c2cn([1]3CCNC[1]3F)nn2)nc(-c2ccc(F)cc2)c1-c1ccncc1	100000	140
JAK2_IA	CHEMBL3120995	Cn1c(-c2cn(-c3ccc(C(=O)O)cc3)nn2)nc(-c2ccc(F)cc2)c1-c1ccncc1	100000	140
JAK2_IA	CHEMBL3314270	Cn1c(-c2cn([1]3[1](O)[1](O)[1](CO)O[1]3O)nn2)nc(-c2ccc(F)cc2)c1-c1ccncc1	100000	140
JAK2_IA	CHEMBL3314271	CSc1nccc(-c2c(-c3ccc(F)cc3)nc3nc(N)ccn23)n1	100000	140
JAK2_IA	CHEMBL3314272	C#Cc1ccn2c(-c3ccnc(SC)n3)c(-c3ccc(F)cc3)nc2n1	100000	140
JAK2_IA	CHEMBL3314273	CSc1nccc(-c2c(-c3ccc(F)cc3)nc3nc(-c4cn(CC(=O)[O-])nn4)ccn23)n1.[Na+]	100000	140
JAK2_IA	CHEMBL3314274	CCOP(=O)(CCn1cc(-c2ccn3c(-c4ccnc(SC)n4)c(-c4ccc(F)cc4)nc3n2)nn1)OCC	100000	140
JAK2_IA	CHEMBL3314275	CSc1nccc(-c2c(-c3ccc(F)cc3)nc3nc(-c4cn(CCP(=O)([O-])[O-])nn4)ccn23)n1.[Na+].[Na+]	100000	140
JAK2_IA	CHEMBL3314285	CSc1nccc(-c2[nH]c(-c3ccc(O)cc3)nc2-c2ccc(F)cc2)n1	100000	140
JAK2_IA	CHEMBL3314286	C#Cc1ccc(-c2nc(-c3ccc(F)cc3)c(-c3ccnc(SC)n3)[nH]2)cc1	100000	140
JAK2_IA	CHEMBL3360840	Nc1nc2cccc(-c3ccc(CN4CCS(=O)(=O)CC4)cc3)n2n1	100000	154

JAK2_IA	CHEMBL3355482	CN(Cc1ccccc1)C(=O)c1ccc(S(=O)(=O)Nc2ccccc2)cc1	100000	154
JAK2_IA	CHEMBL3622924	Cc1cc(C)cc(Nc2nccc(-n3cc(C)c(CN4CC[1](O)C4)c3)n2)c1	115300	167
JAK2_IA	CHEMBL3622956	Cc1nn(-c2ccnc(Nc3ccc4c(c3)c(Cl)c(C)n4C)n2)cc1CN1CC(O)C1	111900	167
JAK2_IA	CHEMBL3622957	Cc1nn(-c2ccnc(Nc3ccc4c(c3)c(Cl)c(C)n4C)n2)cc1CN1CC[1](O)C1	162400	167
JAK2_IA	CHEMBL3622958	Cc1nn(-c2ccnc(Nc3ccc4c(c3)c(C)c(C)n4C)n2)cc1CN1CC(O)C1	138200	167
JAK2_IA	CHEMBL3885292	Cc1cc(Nc2ncc(C#N)c(Nc3cccc(S(=O)(=O)NC(C)(C)C)c3)n2)ccc1N1CCN(C)CC1	100000	194
JAK2_IA	CHEMBL3906494	Cc1c[nH]c2ncc(-c3cccc(NC(=O)/C=C/CN(C)C)c3)cc12	100000	197
JAK2_IA	CHEMBL3919033	C=CC(=O)Nc1cccc(-c2cnc3[nH]cc(-c4cccc(C)c4F)c3c2)c1	100000	197
JAK2_IA	CHEMBL3920230	C=CC(=O)Nc1cccc(-c2cnc3[nH]cc(-c4cc(C)c(F)cc4OC)c3c2)c1	100000	197
JAK2_IA	CHEMBL3921295	CN(C)C/C=C/C(=O)Nc1cccc(-c2cnc3[nH]cc(Cl)c3c2)c1	100000	197
JAK2_IA	CHEMBL3925725	COc1c(F)cc(F)cc1-c1c[nH]c2ncc(-c3cc(NC(=O)/C=C/CN(C)C)ccc3F)cc12	100000	197
JAK2_IA	CHEMBL3934456	C=CC(=O)Nc1cccc(-c2cnc3c(c2)CC(=O)N3)c1	100000	197
JAK2_IA	CHEMBL3943435	Cc1cccc(-c2c[nH]c3ncc(-c4cccc(NC(=O)/C=C/CN(C)C)c4)cc23)c1F	100000	197
JAK2_IA	CHEMBL3948029	COc1cc(F)c(C)cc1-c1c[nH]c2ncc(-c3cccc(NC(=O)/C=C/CN(C)C)c3)cc12	100000	197
JAK2_IA	CHEMBL4098682	Cc1ccc(CNc2nc(N)nc3[nH]c4cc(C)c(O)cc4c23)cc1	100000	224
JAK2_IA	CHEMBL4279618	CN(c1ncnc2[nH]ccc12)[1]1CN(Cc2ccccc2)CC12CC2	98000	239
JAK2_IA	CHEMBL446576	Nc1n[nH]c2cc(-c3ccccc3)ccc12	145300	258
JAK2_IA	CHEMBL501406	Nc1n[nH]c2cc(Br)ccc12	131000	258
JAK2_IA	CHEMBL4585913	Nc1n[nH]c2ccc(NC(=O)c3c(Cl)cccc3-n3cnnn3)cc12	76600	270
JAK2_IA	CHEMBL462168	COc1cc2nccc(Oc3ccc4c(NC(=O)c5cccc(C(F)(F)F)c5)nn(C)c4c3)c2cc1OC	25000000	274
JAK2_IA	CHEMBL4228653	CCNC(=O)c1cc2c(-c3cccc(OCCCCCCOc4ccc(Nc5nc(-c6cnn(CCCCCCC(=O)NO)c6)c6cc[nH]c6n5)cc4)c3)nc(N)nc2s1	100000	275
JAK2_IA	CHEMBL4634634	C[1]1C[1]1C(=O)N1CCN(c2cnc(C#N)c(-c3cnn(C)c3)n2)C[1]1C	100000	275

JAK2_IA	CHEMBL4756015	O=C1C=CC2(C=C1)OCC(=O)N2c1nc(-c2cccc3ccccc23)nn1-c1ccccc1	59000	286

1.5 actives JAK3

Table S5 presents the training set of active Janus kinase inhibitors from the literature for Janus kinase 3, including ChEMBLl ID, SMILES code, and IC50 values

Target	Compound	SMILES	Values	Ref
JAK3_AC	CHEMBL3593777	N#Cc1ccc(N2CC[1](Nc3c(C(N)=O)cnc4[nH]ccc34)[1](F)C2)nc1	0.3	26
JAK3_AC	CHEMBL4549451	COc1ccc(-c2cc3c(N[1](CO)CC4CC4)c(C(N)=O)cnn3c2)cn1	1	27
JAK3_AC	CHEMBL4456730	Cn1cc(Nc2ncnc3c2cnn3Cc2cccc(NC#N)c2)cn1	689.2	32
JAK3_AC	CHEMBL4582632	C=CC(=O)N1CCCC(n2nc(Br)c3c(N)ncnc32)C1	34	32
JAK3_AC	CHEMBL4568087	Cn1cc(-c2cnc3c(-c4csc(C(=O)N[1]5CCCC[1]5N)c4)cnn3c2)cn1	33	33
JAK3_AC	CHEMBL4634591	C[1](Oc1cc(-c2cnn(C3CCOCC3)c2)cnc1N)c1cc(F)ccc1Cl	228	34
JAK3_AC	CHEMBL3601115	C=CC(=O)Nc1cccc(CNc2nc(Nc3cnn(CCOC)c3)ncc2Cl)c1	0.5	35
JAK3_AC	CHEMBL3601223	C=CC(=O)Nc1cccc(CNc2nc(Nc3ccc(N4CCN(C)CC4)cc3OC)ncc2Cl)c1	458	35
JAK3_AC	CHEMBL4096246	CN1CCC(NC(=O)c2ccc(Nc3cc(NCc4ccccc4)c(C(N)=O)cn3)cc2)CC1	4.8	36
JAK3_AC	CHEMBL4644578	C[1]1[1]2CN(c3nc(Nc4cnn(CCO)c4)ncc3F)C[1]12NC(=O)[1]1C[1]1C#N	194	37
JAK3_AC	CHEMBL3360318	COc1ccc(-c2cccc3nc(NC(=O)C4CC4)nn23)cc1	528	39
JAK3_AC	CHEMBL3301607	O=C(Nc1nc2cccc(-c3ccc(CN4CCS(=O)(=O)CC4)cc3)n2n1)C1CC1	149.35	39
JAK3_AC	CHEMBL4085457	C=CC(=O)N1C[1](Nc2ncnc3[nH]ccc23)CC[1]1C	0.3	69
JAK3_AC	CHEMBL4085582	C=CC(=O)Nc1cccc(-c2ncnc3[nH]ccc23)c1	0.3	69
JAK3_AC	PubChem SID: 381324674	CN(C)C(=O)C(=Cc1oc(cc1)c2nc3cnc4[nH]ccc4c3n2C5CCCCC5)C#N	3	70

JAK3_AC	CHEMBL104963	O=C1Nc2ccc(C(=O)c3cccs3)cc2/C1=C/c1ccc[nH]1	26	73
JAK3_AC	CHEMBL4855542	CC(=O)Nc1ccc(/C=C2\C(=O)Nc3ccc(C(=O)c4cccs4)cc32)cc1	700	73
JAK3_AC	CHEMBL1079594	N#Cc1ccc2ncn(-c3ncc4[nH]c(=O)n([1]5CCOc6c(F)cccc65)c4n3)c2c1	1	75
JAK3_AC	CHEMBL1081977	COc1cc(Nc2nc3cccc(-c4ccc(NS(C)(=O)=O)cc4)c3o2)cc(OC)c1OC	130	76
JAK3_AC	CHEMBL1093119	Cc1cc(-c2cnc3cccc(-c4cc(F)c(CN5CCOCC5)c(F)c4)c3n2)ccc1C(=O)N1CCOCC1	130	77
JAK3_AC	CHEMBL131577	Cc1cccc(Cl)c1NC(=O)c1cnc(NC(=O)C2CC2)s1	44	84
JAK3_AC	CHEMBL1427221	O=C(CCl)Nc1cccc(-c2cnc3ccccc3n2)c1	0.2	85
JAK3_AC	CHEMBL1650951	CC(C)(O)C(=O)N1CCN(c2ccc(C(F)(F)F)c(C3=C(c4c[nH]c5ccccc45)C(=O)NC3=O)c2)CC1	0.5	89
JAK3_AC	CHEMBL1684800	COc1ccc(Cn2ncc(NC(=O)c3cc(NC(=O)Nc4ccc(Cl)c(C(F)(F)F)c4)ccc3C)c2N)cc1	1	91
JAK3_AC	CHEMBL1688215	Nc1nccc(-c2ccc3c(N)n[nH]c3c2)n1	990	92
JAK3_AC	CHEMBL1774056	Cc1cc(Nc2nc(N[1](C)c3ccc(F)cn3)c(C#N)cc2F)n[nH]1	7	93
JAK3_AC	CHEMBL1784637	CNC(=O)c1ncc(C#Cc2cc(C(=O)Nc3ccc(CN4CCN(CCO)CC4)c(C(F)(F)F)c3)ccc2C)n1C	57.5	94
JAK3_AC	CHEMBL1789941	N#CC[1](C1CCCC1)n1cc(-c2ncnc3[nH]ccc23)cn1	2	95
JAK3_AC	CHEMBL261386	O=c1[nH]ccc2c3[nH]c(-c4ccccc4Cl)nc3c3ccc(F)cc3c12	77	97
JAK3_AC	CHEMBL189963	CC(=O)c1c(C)c2cnc(Nc3ccc(N4CCNCC4)cn3)nc2n(C2CCCC2)c1=O	63.1	99
JAK3_AC	CHEMBL2035183	C1=C/COCc2cc(ccc2OCCN2CCCC2)Nc2nccc(n2)-c2ccc(s2)COC/1	650	104
JAK3_AC	CHEMBL1983268	CN1CCN(c2ccc(C(=O)Nc3n[nH]c4ccc(Cc5cc(F)cc(F)c5)cc34)c(NC3CCOCC3)c2)CC1	349	105
JAK3_AC	CHEMBL2006765	CCCNC(=O)c1ccc(Nc2nc(NCC(F)(F)F)c3cc[nH]c3n2)cc1	496	107
JAK3_AC	CHEMBL3917405	O=C(CCCCCCOc1ccc2cc1COC/C=C/COCc1cccc(c1)-c1ccnc(n1)N2)NO	10	110
JAK3_AC	CHEMBL2062803	CS(=O)(=O)c1ccc(-c2cccn3nc(Nc4ccc(N5CCOCC5)cc4)nc23)cc1	17	110
JAK3_AC	CHEMBL2103874	CNS(=O)(=O)C[1]1CC[1](N(C)c2[nH]cnc3nccc2-3)CC1	95.1	112
JAK3_AC	CHEMBL3765517	CC(C)[1](C)n1cc(-c2cc(N3CCS(=O)(=O)CC3)n(C)n2)c2[nH]nc(N)c2c1=O	4	113

JAK3_AC	CHEMBL21156	CC(C)(C)c1nc2c3ccc(F)cc3c3c(=O)[nH]ccc3c2[nH]1	5	115
JAK3_AC	CHEMBL215019	CNc1ncc2cc(-c3cc(C(=O)Nc4ccc(Cl)c(C(F)(F)F)c4)ccc3C)ccc2n1	16	116
JAK3_AC	CHEMBL385937	Cc1ccc(C(=O)Nc2cccc(C(F)(F)F)c2)cc1-c1ccc2nc(N)ncc2c1	20	117
JAK3_AC	CHEMBL2181088	Cc1cnc2nc1-c1cccc(c1)COCC=CCOCc1cc(ccc1OCCN1CCCC1)N2	89	121
JAK3_AC	CHEMBL2208035	O=C1CC[1](C(=O)Nc2ccc(-c3ccnc(Nc4ccc(N5CCOCC5)cc4)n3)cc2)N1	61.7	122
JAK3_AC	CHEMBL221959	C[1]1CCN(C(=O)CC#N)C[1]1N(C)c1ncnc2[nH]ccc12	0.24	126
JAK3_AC	CHEMBL2325895	CC(C)(C)[1](NC(=O)c1c[nH]c2ncc(C3CC3)nc12)C(=O)N1CC(C#N)C1	0.26	129
JAK3_AC	CHEMBL234936	O=C1NCc2c1c1c3ccccc3n3c1c1c2c2cc(CO)ccc2n1CC(CO)C3	3	130
JAK3_AC	CHEMBL2376157	C[1](NC(=O)c1c[nH]c2ncc(-c3ncn4cc(F)ccc34)nc12)C(=O)N1CC(C#N)C1	0.3	131
JAK3_AC	CHEMBL2381986	Cc1cc(Nc2nc(N[1](C)c3ccc(F)cn3)c(F)c(N3CCS(=O)(=O)CC3)n2)n[nH]1	4	132
JAK3_AC	CHEMBL240432	c1ccc(-c2c(-c3ccc(OCCn4ccnc4)cc3)oc3nccc(NCCN4CCNCC4)c23)cc1	25	135
JAK3_AC	CHEMBL247468	c1ccc(-c2c(-c3ccc(OCCN4CCCC4)cc3)oc3ncnc(NCCN4CCNCC4)c23)cc1	960	138
JAK3_AC	CHEMBL272424	O=c1[nH]ccc2c3[nH]c(-c4c(F)cccc4Cl)nc3c3ccc(F)cc3c12	0.6	139
JAK3_AC	CHEMBL3116050	C[1](Nc1nc(Nc2cn(C)cn2)c2cc[nH]c2n1)c1ncc(F)cn1	35	142
JAK3_AC	CHEMBL3137308	NC(=O)c1cnc2[nH]ccc2c1N[1]1[1]2CC3C[1]1C[1](O)(C3)C2	0.7	145
JAK3_AC	CHEMBL3137331	CNC(=O)c1ccc(Nc2ncc(C(F)(F)F)c(NCc3nccnc3N(C)S(C)(=O)=O)n2)cc1	114.9	146
JAK3_AC	CHEMBL3234895	CC(C)(O)COCc1cccc(Nc2sc(-c3c(F)cc(C(C)(C)O)cc3F)cc2C(N)=O)n1	763	147
JAK3_AC	CHEMBL4850588	CNC(=O)c1ccc(Nc2ncc(C(F)(F)F)c(NCc3ccccc3Cl)n2)cc1	44.18	147
JAK3_AC	CHEMBL4640647	C=CC(=O)Nc1cccc(Nc2nc(Nc3ccc(B(O)O)cc3)ncc2C(F)(F)F)c1	0.3	151
JAK3_AC	CHEMBL3359927	C[1]1(F)CCC[1]1Nc1c(C(N)=O)cnn2cccc12	1	155
JAK3_AC	CHEMBL3393341	C[1]1CCCN(c2ncnc3[nH]cc(-c4cccc(C#N)c4)c23)C1	54	156
JAK3_AC	CHEMBL3622143	CCn1c(C(=O)N(C2CC2)C2CC2)cc2c3c(ncn3C)c(Nc3nc(C)c(C(=O)NC)s3)nc21	19	157

JAK3_AC	CHEMBL3589169	CN1CCN(C(=O)c2ccc3[nH]c4c(C(N)=O)cc(-c5ccc(Cl)cc5)cc4c3c2)CC1	35	158
JAK3_AC	CHEMBL3622135	CCn1c(C(=O)N(C2CC2)C2CC2)cc2c3c(ncn3C)c(Nc3nc(C)cs3)nc21	23	163
JAK3_AC	CHEMBL3622376	c1nc(-c2cn(CC3CO3)nn2)c2cc[nH]c2n1	35	164
JAK3_AC	CHEMBL3622956	Cc1nn(-c2ccnc(Nc3ccc4c(c3)c(Cl)c(C)n4C)n2)cc1CN1CC(O)C1	78.12	167
JAK3_AC	CHEMBL3642412	CS(=O)(=O)c1ccc(-c2cccn3nc(Nc4cccc([1]5CCN(CC(N)=O)C[1]5O)c4)nc23)cc1	13.23	167
JAK3_AC	CHEMBL3645073	COc1ccc(-c2cccn3nc(Nc4ccc(C5CCN(C(=O)OC(C)(C)C)CC5)cc4)nc23)c(F)c1	999	167
JAK3_AC	CHEMBL4086057	CCc1cc(O)ccc1-c1ccc2c(-c3ncc[nH]3)n[nH]c2c1	0.8	169
JAK3_AC	CHEMBL3645121	CS(=O)(=O)N1CCN(c2ccc(Nc3ncc(C(N)=O)c(NC4CC4)n3)cc2)CC1	0.8	171
JAK3_AC	CHEMBL3650985	C=C(C)C(=O)Nc1cccc(-c2ncnc3[nH]cc(C(=O)OCC)c23)c1	0.15	173
JAK3_AC	CHEMBL3651021	C=C(F)C(=O)Nc1cc(-c2ncnc3[nH]cc(C(=O)OCC)c23)ccc1F	0.013	173
JAK3_AC	CHEMBL3652408	COCCOC1CCCN(S(=O)(=O)C[1]2CC[1](N(C)c3ncnc4[nH]ccc34)CC2)C1	0.0368	175
JAK3_AC	CHEMBL3665180	CC(C)CS(=O)(=O)N1CCC[1](Nc2nc(N)ncc2-c2cnc3[nH]ccc3n2)C1	0.001155	178
JAK3_AC	CHEMBL3667478	CC1(C)CN(c2ccc(C#N)cn2)CC[1]1Nc1c(C(N)=O)cnn2cc(N3CC[1](O)C3=O)cc12	0.2	180
JAK3_AC	CHEMBL4076794	COc1cccc(-c2cc3c(N[1]4CC[1](C)(N)C4(C)C)c(C(N)=O)cnn3c2)c1	0.4	181
JAK3_AC	CHEMBL3671816	CC1(C)[1](Nc2c(C(N)=O)cnn3cc(-c4ccccc4)cc23)CC[1]1(C)O	0.2	182
JAK3_AC	CHEMBL3689514	CC1CN(C(=O)CC#N)C12CCCN(c1ncnc3[nH]ccc13)C2	4	184
JAK3_AC	CHEMBL3689517	N#CCC(=O)N1CC(F)(F)C12CCCN(c1ncnc3[nH]ccc13)C2	4	184
JAK3_AC	CHEMBL4636507	Cc1[nH]nc2[nH]c(=O)cc([1]3CC[1](CC(=O)N4CCC(C)(C)CC4)CC3)c12	57	187
JAK3_AC	CHEMBL388978	CN[1]1C[1]2O[1](C)([1]1OC)n1c3ccccc3c3c4c(c5c6ccccc6n2c5c31)C(=O)NC4	0.0913	195
JAK3_AC	CHEMBL4248202	Cn1cc(-c2nn(C3CCCN(C(=O)CC#N)C3)c3ncnc(N)c23)cn1	32.3	195
JAK3_AC	CHEMBL3895231	CCOC(=O)COc1cccc(Nc2ncc(C)c(-c3cc4ccccc4o3)n2)c1	87.6	198
JAK3_AC	CHEMBL3931093	Brc1ncc(CN2CCC(c3nnn4cnc5[nH]ccc5c34)CC2)s1	15	200

JAK3_AC	CHEMBL3905150	Cn1cc(-c2cn3ncc(C(N)=O)c(N[1]4CCN(c5ccc(C#N)nn5)CC4(C)C)c3n2)cn1	0.5	201
JAK3_AC	CHEMBL3911320	Nc1n[nH]c2ccc(C(=O)N3CCC[1]3c3ccc(Cl)cc3)cc12	219	202
JAK3_AC	CHEMBL3930506	O=C(/C=C/CN1CCCCC1)Nc1cc2c(Nc3ccc(F)c(Cl)c3)ncnc2cn1	793	205
JAK3_AC	CHEMBL3944322	Cn1cc(Nc2ncc(Cl)c(NC3CCC4(CC3)CCN(C(=O)CC#N)C4)n2)cn1	39	208
JAK3_AC	CHEMBL3951665	CN(C)C(=O)/C(C#N)=C/c1ccc(Nc2cnc3[nH]cc(C(=O)NC(C)(C)C)c3n2)cc1	0.2	209
JAK3_AC	CHEMBL3953720	C=CC(=O)Nc1cccc(-n2cnc3cnc(Nc4ccc(N5CCN(C(C)=O)C(C)C5)cc4)nc32)c1	61.2	210
JAK3_AC	CHEMBL4108070	CN(c1ccc(C(=O)Nc2nccs2)cn1)[1]1CCN(c2ncnc3[nH]ccc23)C1	522	212
JAK3_AC	CHEMBL3979920	Cn1cc(-c2nc(N[1]3CCCC[1]3N)c(F)c3c2C(=O)NC3)cn1	114	215
JAK3_AC	CHEMBL4065693	C=CC(=O)Nc1cccc(CNc2cc(Nc3ccccc3)ncc2C(N)=O)c1	1	218
JAK3_AC	CHEMBL4068509	C=CC(=O)N1CCN(CCCn2c(=O)c(-c3c(Cl)c(OC)cc(OC)c3Cl)cc3cnc(NC)nc32)CC1	556	220
JAK3_AC	CHEMBL4095596	CC(CCCCCC(=O)NO)n1cc(-c2ncnc3[nH]ccc23)cn1	436	223
JAK3_AC	CHEMBL4160840	O=C(NO)c1ccc(-c2ccnc(Nc3ccc(N4CCOCC4)cc3)n2)cc1	517	228
JAK3_AC	CHEMBL4175555	CS(=O)(=O)Nc1ccc(-c2ccnc(Nc3ccc(N4CCC(C(=O)Nc5ccc(/C=C/C(=O)NO)cc5)CC4)cc3)n2)cc1	121	228
JAK3_AC	CHEMBL4165758	Cc1cnc(Nc2cc(N3CCOCC3)cc(N3CCOCC3)c2)nc1Nc1cccc(CO)c1	371	229
JAK3_AC	CHEMBL2443026	CN(C)c1ccc(Oc2cc(O)cc(O)c2-c2cc(C(=O)NC3CCN(C4CCC5(CC4)OCCO5)CC3)no2)cc1	10	236
JAK3_AC	CHEMBL4248386	C=CS(=O)(=O)Nc1cccc(-c2ccc(C(N)=O)c3[nH]c(C)c(C)c23)c1C	11	236
JAK3_AC	CHEMBL4277900	CC1=Nc2c(F)cc(-c3nc(Nc4ccc(C5CCN(C)CC5)cn4)ncc3F)cc2C12CCCC2	154	238
JAK3_AC	CHEMBL4293578	CN(c1ncnc2[nH]ccc12)[1]1CN(S(=O)(=O)c2cccc(C#N)c2)CC12CC2	110	239
JAK3_AC	CHEMBL4286867	O=C(CCCCCCC(=O)Nc1ccc(-c2ccnc(Nc3ccc(N4CCOCC4)cc3)n2)cc1)NO	80.1	240
JAK3_AC	CHEMBL4289281	C=CC(=O)Nc1cccc(Nc2nc(Nc3ccc(OC)c(OCCCN4CCOCC4)c3)ncc2Cl)c1	0.69	243
JAK3_AC	CHEMBL4446102	NC(=O)c1cnc2[nH]ccc2c1NC1C2CC3CC1CC(C2)C3O	1	250

JAK3_AC	CHEMBL4466059	O=C(CCCCCCn1cc(Nc2ncc(Cl)c(NC3CC3)n2)cn1)NO	2	253
JAK3_AC	CHEMBL4526485	N#CC[1]1CC[1](n2c(=O)[nH]c3cnc(Nc4cc(Cl)c[nH]c4=O)nc32)CC1	0.7	255
JAK3_AC	CHEMBL4464404	C=CC(=O)Nc1cccc(Oc2nc(Nc3ccc(N4CCOCC4)cc3)nc3[nH]cc(-c4ccncc4)c23)c1	0.6	256
JAK3_AC	CHEMBL4470339	Cc1nc2cnc3[nH]ccc3c2n1[1]1CC[1](CC#N)CC1	12	259
JAK3_AC	CHEMBL4473956	C#CC(=O)N1CCN(c2ccc([N+](=O)[O-])c(C3=C(c4c[nH]c5ccccc45)C(=O)NC3=O)c2)CC1	0.1	260
JAK3_AC	CHEMBL4476770	NC(=O)c1nc(-c2c(F)cccc2Cl)oc1Nc1ccc(C(=O)N2CCOCC2)cc1	41	262
JAK3_AC	CHEMBL4516561	CCS(=O)(=O)Nc1cc(-c2ccc(O)c(F)c2)cc2[nH]ncc12	10	263
JAK3_AC	CHEMBL364623	Cc1cc(Nc2ncc(C(=O)Nc3c(C)cccc3Cl)s2)nc(C)n1	6	265
JAK3_AC	CHEMBL4560698	Cc1cnc(Nc2ccc(N3CCC(N(C)CCCO)CC3)c(F)c2)nc1-c1cnn(C(C)C)c1	23	266
JAK3_AC	CHEMBL460472	COc1ccc2c(Oc3ccc(NC(=O)c4c(C)n(CC(C)(C)O)n(-c5ccccc5)c4=O)nc3)ccnc2c1	1000	272
JAK3_AC	CHEMBL461139	O=c1[nH]cnc2[nH]c(-c3ccnc(/C=C/c4ccc(CN5CCOCC5)cc4)c3)cc12	310	273
JAK3_AC	CHEMBL4635876	OCCn1cc(Nc2ncc(Cl)c(NCc3cc(F)ccc3F)n2)cn1	1	276
JAK3_AC	CHEMBL4635399	C=CC(=O)Nc1cccc(Nc2nc(Nc3ccc(NC(=O)CN4CCOCC4)cc3)ncc2Cl)c1	10	277
JAK3_AC	CHEMBL4641006	N#C[1]1(C2CC2)C(=O)N2C[1]1COCC(=O)NCCCc1cccc(n1)Nc1cc2ccn1	43	278
JAK3_AC	CHEMBL4644662	C=C(C#N)C(O)c1ccc(-c2cccc3nc(NC(=O)C4CC4)nn23)cc1	285	279
JAK3_AC	CHEMBL4645565	CC(O)(C#Cc1ccc(N2CCOCC2)c(Nc2ncnc3[nH]ccc23)c1)c1nccs1	892	280
JAK3_AC	CHEMBL4646281	C=CC(=O)NCCOc1cc(Cc2ccccc2)cc2[nH]c(-c3n[nH]c4cc(C(F)F)ccc34)cc12	320	281
JAK3_AC	CHEMBL4748313	C=CC(=O)Nc1cccc(Cn2ncc3cnc(Nc4cnn(CCOC)c4)nc32)c1	0.1	284
JAK3_AC	CHEMBL4789273	Cc1ccc(C(=O)/C=C/C(=O)N2CCc3ccc(Nc4ncc(C)c(-c5cnn(C(C)C)c5)n4)cc3C2)cc1	28	287
JAK3_AC	CHEMBL4795804	O=C(Nc1c[nH]nc1-c1nc(-c2ccc(CN3CCCCC3)cc2)c[nH]1)c1cccc(Cl)c1	13	290
JAK3_AC	CHEMBL4867261	N#CCCn1cc(-n2c(-c3cccs3)cc3cnc4[nH]ccc4c32)cn1	160	293
JAK3_AC	CHEMBL4870513	COc1cc(Nc2nc(NCc3cccc(NC(=O)CCl)c3)n3ccnc3c2C(N)=O)cc(OC)c1	562	294

JAK3_AC	CHEMBL495727	O=C(Nc1c[nH]nc1-c1nc2ccc(CN3CCOCC3)cc2[nH]1)NC1CC1	1	296
JAK3_AC	CHEMBL564940	COC(=O)[1]1(O)Cn2c3ccccc3c3c4c(c5c6ccccc6n(c5c32)C[1]1O)C(=O)NC4=O	1	307
JAK3_AC	CHEMBL583895	C[1](Nc1cc(-c2c(N)nn3cccnc23)ncn1)c1ccc(F)cc1	7	309
JAK3_AC	CHEMBL584944	CCC[1](Nc1cc(-c2c(N)nn3cccnc23)ncn1)c1ccc(F)cc1	10	309
JAK3_AC	CHEMBL3644620	CCc1cc(O)c(F)cc1-c1ccc2c(-c3nc4c([nH]3)CCN(C(=O)c3cnc(N5CCCCC5)cn3)C4)n[nH]c2c1	0.5	257

1.6.inactives JAK3

Table S6 presents the training set of inacctives (IAs) Janus kinase inhibitors from the literature for Janus kinase 3, ChEMBLl ID, SMILES code, and IC50 values

Target	Compound	SMILES	Values	Ref
JAK3_IA	CHEMBL1446198	CCC(=O)Nc1cccc(-c2cnc3ccccc3n2)c1	50000	85
JAK3_IA	CHEMBL1537091	CNC(=S)Nc1cccc(-c2cnc3ccccc3n2)c1	50000	85
JAK3_IA	CHEMBL1553519	CS(=O)(=O)Nc1cccc(-c2cnc3ccccc3n2)c1	50000	85
JAK3_IA	CHEMBL1572928	O=C(Nc1cccc(-c2cnc3ccccc3n2)c1)c1ccco1	50000	85
JAK3_IA	CHEMBL3645535	CN(C)CC(=O)Nc1cccc(-c2cnc3ccccc3n2)c1	50000	85
JAK3_IA	CHEMBL3645538	O=C(CCCCl)Nc1cccc(-c2cnc3ccccc3n2)c1	50000	85
JAK3_IA	CHEMBL3645539	O=C(Cn1cncn1)Nc1cccc(-c2cnc3ccccc3n2)c1	50000	85
JAK3_IA	CHEMBL3645542	COc1ccc(NC(=O)Nc2cccc(-c3cnc4ccccc4n3)c2)cc1	50000	85
JAK3_IA	CHEMBL3645543	O=S(=O)(Nc1cccc(-c2cnc3ccccc3n2)c1)c1cccs1	50000	85
JAK3_IA	CHEMBL3645544	O=C(Nc1cccc(-c2cnc3ccccc3n2)c1)C1CC1	50000	85
JAK3_IA	CHEMBL3645546	CN1CCN(CC(=O)Nc2cccc(-c3cnc4ccccc4n3)c2)CC1	50000	85
JAK3_IA	CHEMBL3645548	O=C(CCCl)Nc1cccc(-c2cnc3ccccc3n2)c1	50000	85
JAK3_IA	CHEMBL185140	O=C(CCc1cn(Cc2ccc(Cl)cc2)c2ccccc12)Nc1ccncc1	52000	98
JAK3_IA	CHEMBL1938646	Nc1ncc(C(=O)NCc2ccccc2)c2sc(-c3ccc(N4CCOCC4)cc3)cc12	125000	103
JAK3_IA	CHEMBL4283458	COc1cc2c(=O)n(C3CCN(C)CC3)c3c4cc[nH]c4ncc3c2cc1OC	61570	127
JAK3_IA	CHEMBL4284499	COc1cc2c(=O)n(CC3CCCCC3)c3c4cc[nH]c4ncc3c2cc1OC	76900	127
JAK3_IA	CHEMBL278041	Oc1ccc(-c2nc(-c3ccc(F)cc3)c(-c3ccncc3)[nH]2)cc1	100000	140
JAK3_IA	CHEMBL3120960	Cn1c(-c2c[nH]nn2)nc(-c2ccc(F)cc2)c1-c1ccncc1	100000	140
JAK3_IA	CHEMBL3120963	Cn1c(-c2cn(CCO)nn2)nc(-c2ccc(F)cc2)c1-c1ccncc1	100000	140
JAK3_IA	CHEMBL3120969	CCOP(=O)(CCn1cc(-c2nc(-c3ccc(F)cc3)c(-c3ccncc3)n2C)nn1)OCC	100000	140
JAK3_IA	CHEMBL3120970	C[1](Nc1nccc(-c2c(-c3ccc(F)cc3)nc3nc(N)ccn23)n1)c1ccccc1	100000	140

JAK3_IA	CHEMBL3120983	Cn1c(-c2cn(-c3cccc(C(=O)O)c3)nn2)nc(-c2ccc(F)cc2)c1-c1ccncc1	100000	140
JAK3_IA	CHEMBL3120995	Cn1c(-c2cn(-c3ccc(C(=O)O)cc3)nn2)nc(-c2ccc(F)cc2)c1-c1ccncc1	100000	140
JAK3_IA	CHEMBL3314270	Cn1c(-c2cn([1]3[1](O)[1](O)[1](CO)O[1]3O)nn2)nc(-c2ccc(F)cc2)c1-c1ccncc1	100000	140
JAK3_IA	CHEMBL3314271	CSc1nccc(-c2c(-c3ccc(F)cc3)nc3nc(N)ccn23)n1	100000	140
JAK3_IA	CHEMBL3314272	C#Cc1ccn2c(-c3ccnc(SC)n3)c(-c3ccc(F)cc3)nc2n1	100000	140
JAK3_IA	CHEMBL3314274	CCOP(=O)(CCn1cc(-c2ccn3c(-c4ccnc(SC)n4)c(-c4ccc(F)cc4)nc3n2)nn1)OCC	100000	140
JAK3_IA	CHEMBL3314276	C#Cc1ccc(-c2nc(-c3ccc(F)cc3)c(-c3cc[nH]c(=O)c3)[nH]2)cc1	100000	140
JAK3_IA	CHEMBL3314285	CSc1nccc(-c2[nH]c(-c3ccc(O)cc3)nc2-c2ccc(F)cc2)n1	78300	140
JAK3_IA	CHEMBL3314286	C#Cc1ccc(-c2nc(-c3ccc(F)cc3)c(-c3ccnc(SC)n3)[nH]2)cc1	100000	140
JAK3_IA	CHEMBL3120985	Cn1c(-c2cn(CCP(=O)([O-])[O-])nn2)nc(-c2ccc(F)cc2)c1-c1ccncc1.[Na+].[Na+]	100000	140
JAK3_IA	CHEMBL3355482	CN(Cc1ccccc1)C(=O)c1ccc(S(=O)(=O)Nc2ccccc2)cc1	100000	154
JAK3_IA	CHEMBL3885292	Cc1cc(Nc2ncc(C#N)c(Nc3cccc(S(=O)(=O)NC(C)(C)C)c3)n2)ccc1N1CCN(C)CC1	100000	194
JAK3_IA	CHEMBL104466	COc1cc2ncc(N[1]3CC[1](O)CC3)nc2cc1OC	50000	311
JAK3_IA	CHEMBL156277	COc1ccccc1C1=C(Nc2cccc(Cl)c2)C(=O)NC1=O	50000	312
JAK3_IA	CHEMBL4875188	COc1ccccc1C1=C(Nc2cccc(C#N)c2)C(=O)NC1=O	50000	312
JAK3_IA	CHEMBL258694	Cc1nnc(-c2cn3ncnc(Nc4cnc5[nH]ccc5c4)c3c2C(C)C)o1	50000	313
JAK3_IA	CHEMBL261592	Cc1cc2cc(Nc3ncnn4cc(-c5nnc(C)o5)c(C(C)C)c34)cnc2[nH]1	50000	313
JAK3_IA	CHEMBL259922	Cc1ccc(C(=O)Nc2ccon2)cc1Nc1ncnc2c1cnn2-c1ccccc1	50000	314
JAK3_IA	CHEMBL377300	Cc1cc2c(F)c(Oc3ncnn4cc(OC[1](C)O)c(C)c34)ccc2[nH]1	50000	315
JAK3_IA	CHEMBL377734	O=C(NC1CC1)c1cc(NCc2cnc(Nc3ccccn3)s2)c(F)cc1F	50000	316
JAK3_IA	CHEMBL383172	Cc1ccc(C(=O)Nc2ccon2)cc1Nc1ncnc(NC2CCCC2)c1C#N	50000	317

1.7 actives TYK2

Table S7 presents the training set of active Janus kinase inhibitors from the literature for Tyrosine kinase 2, including the ChEMBLl ID, SMILES code, and IC50 values

Target	Compound	SMILES	Values	Ref
TYK2_AC	CHEMBL4472924	NC(=O)c1cnn2cc(-c3ccccc3)cc2c1NC1CCCC1	20	27
TYK2_AC	CHEMBL4568087	Cn1cc(-c2cnc3c(-c4csc(C(=O)N[1]5CCCC[1]5N)c4)cnn3c2)cn1	52	33
TYK2_AC	CHEMBL3601120	C=CC(=O)Nc1cccc(CNc2nc(Nc3cnn(CCO)c3)ncc2Cl)c1	140	35
TYK2_AC	CHEMBL4644138	N#C[1]1C[1]1C(=O)N[1]12C[1]1CN(c1nc(Nc3cnn(CCO)c3)ncc1F)C2	29	37
TYK2_AC	CHEMBL3426891	CN1CCN(c2ccc(Nc3ncc(Cl)c(NC[1]4CCCO4)n3)cc2)CC1	5	38
TYK2_AC	CHEMBL3360318	COc1ccc(-c2cccc3nc(NC(=O)C4CC4)nn23)cc1	519	39
TYK2_AC	CHEMBL4078799	Cn1cc(-c2ccc(Nc3nn([1]4COCC[1]4C#N)cc3C(N)=O)cc2)cn1	0.2	66
TYK2_AC	CHEMBL1081484	COc1cc(Nc2nc3cccc(-c4ccc(C(=O)N5CCOCC5)c(F)c4)c3o2)cc(OC)c1OC	63	76
TYK2_AC	CHEMBL1092925	CN1CCN(c2ccc(-c3cnc4cccc(-c5cc(F)c(CN6CCS(=O)(=O)CC6)c(F)c5)c4n3)cn2)CC1	86	77
TYK2_AC	CHEMBL1090360	O=C(Cc1ccccc1)Nc1cccc(-c2nc3sccn3c2-c2ccnc(Nc3cccc(N4CCOCC4)c3)n2)c1	1000	78
TYK2_AC	CHEMBL1789941	N#CC[1](C1CCCC1)n1cc(-c2ncnc3[nH]ccc23)cn1	0.4	96
TYK2_AC	CHEMBL221959	C[1]1CCN(C(=O)CC#N)C[1]1N(C)c1ncnc2[nH]ccc12	0.4	96
TYK2_AC	CHEMBL261386	O=c1[nH]ccc2c3[nH]c(-c4ccccc4Cl)nc3c3ccc(F)cc3c12	7	97
TYK2_AC	CHEMBL2035052	C1=C/COCc2cc(ccc2OCCN2CCCC2)Nc2nccc(n2)-c2ccnc(c2)OCC/1	30	105
TYK2_AC	CHEMBL3917405	O=C(CCCCCCOc1ccc2cc1COC/C=C/COCc1cccc(c1)-c1ccnc(n1)N2)NO	10	110
TYK2_AC	CHEMBL2103874	CNS(=O)(=O)C[1]1CC[1](N(C)c2[nH]cnc3nccc2-3)CC1	75.1	112
TYK2_AC	CHEMBL2105759	CCS(=O)(=O)N1CC(CC#N)(n2cc(-c3ncnc4[nH]ccc34)cn2)C1	7.28	113
TYK2_AC	CHEMBL3763213	CC(C)[1](C)n1cc(-c2cc(N3CCOCC3)n(C)n2)c2[nH]nc(N)c2c1=O	0.55	113

TYK2_AC	CHEMBL21156	CC(C)(C)c1nc2c3ccc(F)cc3c3c(=O)[nH]ccc3c2[nH]1	5	114
TYK2_AC	CHEMBL2181086	CCN(CC)CCC(=O)Nc1cc2cc(c1)Nc1nccc(n1)-c1cccc(c1)COCC=CCOC2	18	122
TYK2_AC	CHEMBL2208034	O=C(Nc1ccc(-c2ccnc(Nc3ccc(N4CCOCC4)cc3)n2)cc1)[1]1CCCN1	348.3	123
TYK2_AC	CHEMBL4278842	COC(=O)Cn1c(=O)c2cc(OC)c(OC)cc2c2cnc3[nH]ccc3c21	18.42	127
TYK2_AC	CHEMBL2322135	c1cc2c(ncc3[nH]nc(C4CCCCC4)c32)[nH]1	50	129
TYK2_AC	CHEMBL2443138	CN1CCC(NC(=O)c2cc(-c3c(O)cc(O)cc3Oc3ccc(N(C)C)cc3)on2)CC1	10	137
TYK2_AC	CHEMBL4874046	CNC(=O)c1ccc(Nc2ncc(C(F)(F)F)c(Nc3ccccc3OC)n2)cc1	21.94	147
TYK2_AC	CHEMBL3301607	O=C(Nc1nc2cccc(-c3ccc(CN4CCS(=O)(=O)CC4)cc3)n2n1)C1CC1	65.3	150
TYK2_AC	CHEMBL4761365	CNC(=O)c1cnc2[nH]ccc2c1N[1]1CCN(Cc2ccc(Cl)cc2)[1](C)C1	940	150
TYK2_AC	CHEMBL4787133	CNC(=O)c1cnc2[nH]ccc2c1NC1CCCN(Cc2cccc(OC)c2)CC1	42.7	150
TYK2_AC	CHEMBL3359927	C[1]1(F)CCC[1]1Nc1c(C(N)=O)cnn2cccc12	2	155
TYK2_AC	CHEMBL3545215	CCn1c(C(=O)N(C2CC2)C2CC2)cc2c3c(ncn3C)c(Nc3cc(C)n(C)n3)nc21	66	158
TYK2_AC	CHEMBL3613597	CC(C)c1nn(C)cc1-c1nc2c(N3CCN(Cc4cccnc4)CC3)c(Br)cnc2[nH]1	10	162
TYK2_AC	CHEMBL3621496	CN1CCN(C(=O)c2ccc3c(c2)[nH]c2c(C(N)=O)ccc(-c4ccccc4F)c23)CC1	753	163
TYK2_AC	CHEMBL3622134	CCn1c(C(=O)N(C2CC2)C2CC2)cc2c3c(ncn3C)c(Nc3nccs3)nc21	28	165
TYK2_AC	CHEMBL3644618	CCc1cc(O)c(F)cc1-c1ccc2c(-c3nc4c([nH]3)CCN(C(=O)c3ccc(F)cc3)C4)n[nH]c2c1	0.8	169
TYK2_AC	CHEMBL4279720	C[1]1(NC#N)CCc2ccc(-c3ncnc4[nH]ccc34)cc21	179	170
TYK2_AC	CHEMBL3658867	CN(c1ncnc2[nH]ccc12)[1]1C[1](CS(=O)(=O)c2ccc(F)c(Cl)c2)C1	242	176
TYK2_AC	CHEMBL4062062	CC1(C)[1](Nc2c(C(N)=O)cnn3cc(-c4ccc(Cl)cc4)cc23)CC[1]1(C)N	10	181
TYK2_AC	CHEMBL3694581	Clc1cnc2nc1NCc1cccc(c1)OCCCc1cccc(c1)N2	25	185
TYK2_AC	CHEMBL4643061	Cc1[nH]nc2[nH]c(=O)cc([1]3C[1]4C[1]3C[1]4CC#N)c12	20	187

TYK2_AC	CHEMBL388978	CN[1]1C[1]2O[1](C)([1]1OC)n1c3ccccc3c3c4c(c5c6ccccc6n2c5c31)C(=O)NC4	0.205	196
TYK2_AC	CHEMBL3942741	FC[1]1CC[1](c2nnn3cnc4[nH]ccc4c23)CC1	11	200
TYK2_AC	CHEMBL3911320	Nc1n[nH]c2ccc(C(=O)N3CCC[1]3c3ccc(Cl)cc3)cc12	279	202
TYK2_AC	CHEMBL3938113	O=C1NCN([1]2CC[1](CNC3(C(F)(F)F)CC3)CC2)c2c1cnc1[nH]ccc21	19	207
TYK2_AC	CHEMBL3944322	Cn1cc(Nc2ncc(Cl)c(NC3CCC4(CC3)CCN(C(=O)CC#N)C4)n2)cn1	1	208
TYK2_AC	CHEMBL4093318	NC(=O)c1cnn2cc(-c3ccccc3)cc2c1NCc1ccccc1	118	218
TYK2_AC	CHEMBL4072239	NC(=O)c1cnc(Nc2ccccc2)cc1NCc1ccccc1	774	219
TYK2_AC	CHEMBL4068357	C[1]1CCN(C(=O)N2CCCC2)C[1]1N(C)c1ncnc2[nH]ccc12	128	220
TYK2_AC	CHEMBL4088216	CN1C(=O)[1](N2CCc3cn(Cc4ccccc4)nc3C2=O)COc2ccccc21	1000	221
TYK2_AC	CHEMBL4549667	CN1C(=O)[1](N2CCc3c(nn(Cc4ccccc4)c3Br)C2=O)COc2ccccc21	1000	222
TYK2_AC	CHEMBL4095596	CC(CCCCCC(=O)NO)n1cc(-c2ncnc3[nH]ccc23)cn1	144	223
TYK2_AC	CHEMBL4175555	CS(=O)(=O)Nc1ccc(-c2ccnc(Nc3ccc(N4CCC(C(=O)Nc5ccc(/C=C/C(=O)NO)cc5)CC4)cc3)n2)cc1	46	228
TYK2_AC	CHEMBL4224807	CN(c1ncnc2[nH]ccc12)[1]1CCN(C(=O)CC#N)C1	110	230
TYK2_AC	CHEMBL4228766	C1=C/COc2ccc(o2)-c2ccnc(n2)Nc2ccc(OCCN3CCCC3)c(c2)COC/1	230	232
TYK2_AC	CHEMBL4238926	NC(=O)c1cnc2[nH]ccc2c1NC1C2CC3CC1CC(O)(C3)C2	8.3	233
TYK2_AC	CHEMBL4590082	Cc1cnc(Nc2ccc(N3CCOCC3)cc2)nc1-c1cc2c(o1)CCN(C(=O)CC#N)C2	3	233
TYK2_AC	CHEMBL4293578	CN(c1ncnc2[nH]ccc12)[1]1CN(S(=O)(=O)c2cccc(C#N)c2)CC12CC2	25	239
TYK2_AC	CHEMBL4286867	O=C(CCCCCCC(=O)Nc1ccc(-c2ccnc(Nc3ccc(N4CCOCC4)cc3)n2)cc1)NO	79.4	240
TYK2_AC	CHEMBL4287153	CNc1nc2sc(-c3cccc([1](C)NC(=O)c4ccccc4S(C)(=O)=O)c3)nc2c2c1ncn2C	27	241
TYK2_AC	CHEMBL4289426	CNc1cc(Nc2cc(C)cc(C)c2)nn2c(C(=O)NCC(C)(C)CO)cnc12	2	244
TYK2_AC	CHEMBL4435170	[2H]C([2H])([2H])NC(=O)c1nnc(NC(=O)C2CC2)cc1Nc1cccc(-c2ncn(C)n2)c1OC	0.2	247

TYK2_AC	CHEMBL4530719	CS(=O)(=O)c1ccccc1Nc1cc(Nc2ccc(F)cn2)ncc1C(N)=O	0.32	249
TYK2_AC	CHEMBL4446102	NC(=O)c1cnc2[nH]ccc2c1NC1C2CC3CC1CC(C2)C3O	5	250
TYK2_AC	CHEMBL4845662	Cc1cnc(Nc2ccc3c(c2)CNC3=O)nc1-c1cnn(CCC#N)c1	0.67	251
TYK2_AC	CHEMBL4466059	O=C(CCCCCCn1cc(Nc2ncc(Cl)c(NC3CC3)n2)cn1)NO	12	253
TYK2_AC	CHEMBL4463007	N#CC[1]1CC[1](n2c(=O)[nH]c3cnc(Nc4ccc[nH]c4=O)nc32)CC1	130	255
TYK2_AC	CHEMBL4476770	NC(=O)c1nc(-c2c(F)cccc2Cl)oc1Nc1ccc(C(=O)N2CCOCC2)cc1	0.6	262
TYK2_AC	CHEMBL4534573	CCc1cc(O)c(F)cc1-c1cc(NS(=O)(=O)N(C)C)c2cn[nH]c2c1	158.49	263
TYK2_AC	CHEMBL4520790	Cn1cc(Nc2nccc(N3C[1]4CC[1](C3)N4C(=O)NCC#N)n2)cn1	16	264
TYK2_AC	CHEMBL4645110	N#C[1]1(C2CC2)C(=O)N2C[1]1COC/C=C/COCc1cccc(n1)Nc1cc2ccn1	12	278
TYK2_AC	CHEMBL4740298	Cn1cc(-c2cn3nccc3c(-c3cnn([1]4(CC#N)C[1](C#N)C4)c3)n2)cn1	6	282
TYK2_AC	CHEMBL4759953	Cc1ccc(C(=O)/C=C/C(=O)N2CCc3cc(Nc4ncc(C)c(-c5cnn(C(C)C)c5)n4)ccc3C2)cc1	100	287
TYK2_AC	CHEMBL4789273	Cc1ccc(C(=O)/C=C/C(=O)N2CCc3ccc(Nc4ncc(C)c(-c5cnn(C(C)C)c5)n4)cc3C2)cc1	30	287
TYK2_AC	CHEMBL4789639	[2H]C([2H])([2H])NC(=O)c1cnc(NC(=O)C2CC2)cc1Nc1cccc(-c2ncc(F)cn2)c1OC	0.19	289
TYK2_AC	CHEMBL4870513	COc1cc(Nc2nc(NCc3cccc(NC(=O)CCl)c3)n3ccnc3c2C(N)=O)cc(OC)c1	1000	294
TYK2_AC	CHEMBL495727	O=C(Nc1c[nH]nc1-c1nc2ccc(CN3CCOCC3)cc2[nH]1)NC1CC1	1	295

1.8 inactives TYK2

Table S8 presents the training set of inacctives (IAs) Janus kinase inhibitors from the literature Tyrosine kinase 2, ChEMBLl ID, SMILES code, and IC50 values

Target	Compound	SMILES	Values	Ref
TYK2_IA	CHEMBL1446198	CCC(=O)Nc1cccc(-c2cnc3ccccc3n2)c1	50000	85
TYK2_IA	CHEMBL1537091	CNC(=S)Nc1cccc(-c2cnc3ccccc3n2)c1	50000	85
TYK2_IA	CHEMBL1553519	CS(=O)(=O)Nc1cccc(-c2cnc3ccccc3n2)c1	50000	85

TYK2_IA	CHEMBL1572928	O=C(Nc1cccc(-c2cnc3ccccc3n2)c1)c1ccco1	50000	85
TYK2_IA	CHEMBL3645535	CN(C)CC(=O)Nc1cccc(-c2cnc3ccccc3n2)c1	50000	85
TYK2_IA	CHEMBL3645538	O=C(CCCCl)Nc1cccc(-c2cnc3ccccc3n2)c1	50000	85
TYK2_IA	CHEMBL3645539	O=C(Cn1cncn1)Nc1cccc(-c2cnc3ccccc3n2)c1	50000	85
TYK2_IA	CHEMBL3645542	COc1ccc(NC(=O)Nc2cccc(-c3cnc4ccccc4n3)c2)cc1	50000	85
TYK2_IA	CHEMBL3645543	O=S(=O)(Nc1cccc(-c2cnc3ccccc3n2)c1)c1cccs1	50000	85
TYK2_IA	CHEMBL3645544	O=C(Nc1cccc(-c2cnc3ccccc3n2)c1)C1CC1	50000	85
TYK2_IA	CHEMBL3645546	CN1CCN(CC(=O)Nc2cccc(-c3cnc4ccccc4n3)c2)CC1	50000	85
TYK2_IA	CHEMBL3645548	O=C(CCCl)Nc1cccc(-c2cnc3ccccc3n2)c1	50000	85
TYK2_IA	CHEMBL3645549	O=C(CN1CCOCC1)Nc1cccc(-c2cnc3ccccc3n2)c1	50000	85
TYK2_IA	CHEMBL3645550	CC(Cl)C(=O)Nc1cccc(-c2cnc3ccccc3n2)c1	50000	85
TYK2_IA	CHEMBL3622826	NCCn1c(C2CCNCC2)nc2cc(C(N)=O)ccc21	759500	114
TYK2_IA	CHEMBL3655080	CN(c1ncnc2[nH]ccc12)[1]1C[1](NS(=O)(=O)CC(F)(F)F)C1	200000	225
TYK2_IA	CHEMBL3655082	CC(C)CS(=O)(=O)N[1]1C[1](N(C)c2ncnc3[nH]ccc23)C1	200000	225
TYK2_IA	CHEMBL3655086	CN(c1ncnc2[nH]ccc12)[1]1C[1](NS(=O)(=O)CC2COC2)C1	200000	225
TYK2_IA	CHEMBL3655095	CN(c1ncnc2[nH]ccc12)[1]1C[1](NS(=O)(=O)CC2CC2)C1	200000	225
TYK2_IA	CHEMBL3655097	CN(c1ncnc2[nH]ccc12)[1]1C[1](NS(=O)(=O)NCC2CC2)C1	200000	225
TYK2_IA	CHEMBL3655104	CN(CCC#N)S(=O)(=O)N[1]1C[1](N(C)c2ncnc3[nH]ccc23)C1	200000	225
TYK2_IA	CHEMBL3658860	CCCCS(=O)(=O)C[1]1C[1](N(C)c2ncnc3[nH]ccc23)C1	200000	225
TYK2_IA	CHEMBL4059667	CN(c1ncnc2[nH]ccc12)[1]1C[1](NS(=O)(=O)C2CC2)C1	200000	225
TYK2_IA	CHEMBL4059894	CCCNS(=O)(=O)C[1]1C[1](N(C)c2ncnc3[nH]ccc23)C1	200000	225
TYK2_IA	CHEMBL4063642	CN(C1CC1)S(=O)(=O)N[1]1C[1](N(C)c2ncnc3[nH]ccc23)C1	200000	225

TYK2_IA	CHEMBL4065613	COCCS(=O)(=O)N[1]1C[1](N(C)c2ncnc3[nH]ccc23)C1	200000	225
TYK2_IA	CHEMBL4066404	CCCCN(C)S(=O)(=O)C[1]1C[1](N(C)c2ncnc3[nH]ccc23)C1	200000	225
TYK2_IA	CHEMBL4066876	CN(c1ncnc2[nH]ccc12)[1]1C[1](CS(=O)(=O)NCC2CC2)C1	200000	225
TYK2_IA	CHEMBL4068319	CN(c1ncnc2[nH]ccc12)[1]1C[1](NS(=O)(=O)CC2CCC2)C1	200000	225
TYK2_IA	CHEMBL4071850	CCNS(=O)(=O)N[1]1C[1](N(C)c2ncnc3[nH]ccc23)C1	200000	225
TYK2_IA	CHEMBL4075575	CN(c1ncnc2[nH]ccc12)[1]1C[1](CS(=O)(=O)C2CC2)C1	200000	225
TYK2_IA	CHEMBL4077614	CN(c1ncnc2[nH]ccc12)[1]1C[1](NS(=O)(=O)N2CCCCC2)C1	200000	225
TYK2_IA	CHEMBL4078655	CN(c1ncnc2[nH]ccc12)[1]1C[1](NS(=O)(=O)NC2CC2)C1	200000	225
TYK2_IA	CHEMBL4079179	CN(c1ncnc2[nH]ccc12)[1]1C[1](NS(=O)(=O)CCCC(F)(F)F)C1	200000	225
TYK2_IA	CHEMBL4082035	CN(c1ncnc2[nH]ccc12)[1]1C[1](NS(=O)(=O)NCCCC#N)C1	200000	225
TYK2_IA	CHEMBL4085011	CN(c1ncnc2[nH]ccc12)[1]1C[1](NS(=O)(=O)Cc2ccccc2)C1	200000	225
TYK2_IA	CHEMBL4085264	CCN(C)S(=O)(=O)N[1]1C[1](N(C)c2ncnc3[nH]ccc23)C1	200000	225
TYK2_IA	CHEMBL4087044	COCCCS(=O)(=O)N[1]1C[1](N(C)c2ncnc3[nH]ccc23)C1	200000	225
TYK2_IA	CHEMBL4087961	CCCCS(=O)(=O)N[1]1C[1](N(C)c2ncnc3[nH]ccc23)C1	200000	225
TYK2_IA	CHEMBL4089286	CCNS(=O)(=O)C[1]1C[1](N(C)c2ncnc3[nH]ccc23)C1	200000	225
TYK2_IA	CHEMBL4090590	CCCS(=O)(=O)C[1]1C[1](N(C)c2ncnc3[nH]ccc23)C1	200000	225
TYK2_IA	CHEMBL4092805	CCS(=O)(=O)N[1]1C[1](N(C)c2ncnc3[nH]ccc23)C1	200000	225
TYK2_IA	CHEMBL4093643	CCCCNS(=O)(=O)C[1]1C[1](N(C)c2ncnc3[nH]ccc23)C1	200000	225
TYK2_IA	CHEMBL4093955	CN(CC1CC1)S(=O)(=O)C[1]1C[1](N(C)c2ncnc3[nH]ccc23)C1	200000	225
TYK2_IA	CHEMBL4098094	CCCNS(=O)(=O)N[1]1C[1](N(C)c2ncnc3[nH]ccc23)C1	200000	225
TYK2_IA	CHEMBL4099048	CN(c1ncnc2[nH]ccc12)[1]1C[1](NS(=O)(=O)CCCC#N)C1	200000	225
TYK2_IA	CHEMBL4100048	CCS(=O)(=O)C[1]1C[1](N(C)c2ncnc3[nH]ccc23)C1	200000	225

TYK2_IA	CHEMBL4100431	CN(c1ncnc2[nH]ccc12)[1]1C[1](NS(C)(=O)=O)C1	200000	225
TYK2_IA	CHEMBL4101374	CN(c1ncnc2[nH]ccc12)[1]1C[1](NS(=O)(=O)c2ccccc2)C1	200000	225
TYK2_IA	CHEMBL4101725	CCCS(=O)(=O)N[1]1C[1](N(C)c2ncnc3[nH]ccc23)C1	200000	225
TYK2_IA	CHEMBL4103435	CN(c1ncnc2[nH]ccc12)[1]1C[1](NS(=O)(=O)CCC(F)(F)F)C1	200000	225
TYK2_IA	CHEMBL4103586	CN(c1ncnc2[nH]ccc12)[1]1C[1](CS(=O)(=O)c2ccccc2)C1	200000	225
TYK2_IA	CHEMBL4103698	CN(c1ncnc2[nH]ccc12)[1]1C[1](NS(=O)(=O)c2ccc(F)cc2)C1	200000	225
TYK2_IA	CHEMBL4104105	CN(c1ncnc2[nH]ccc12)[1]1C[1](CS(=O)(=O)N2CCCC2)C1	200000	225
TYK2_IA	CHEMBL4289149	N#Cc1cc(Nc2cc(NC3CC3)c3ncc(C(N)=O)n3n2)cc(C(F)(F)F)c1	50000	242
TYK2_IA	CHEMBL4285755	Cc1cc(C)cc(Nc2cc(NC3CC3)c3ncc(C(N)=O)n3n2)c1	50000	244
TYK2_IA	CHEMBL4440718	[2H]C([2H])([2H])NC(=O)c1nnc(Nc2ccccn2)cc1Nc1ccccc1S(C)(=O)=O	50000	244
TYK2_IA	CHEMBL4444178	[2H]C([2H])([2H])NC(=O)c1nnc(NC(=O)C2CC2)cc1Nc1ccccc1S(C)(=O)=O	50000	244
TYK2_IA	CHEMBL4526283	CNC(=O)c1cnc(Nc2ccc(F)cn2)cc1Nc1ccccc1S(C)(=O)=O	50000	244
TYK2_IA	CHEMBL4634634	C[1]1C[1]1C(=O)N1CCN(c2cnc(C#N)c(-c3cnn(C)c3)n2)C[1]1C	100000	275
TYK2_IA	CHEMBL4076767	COc1cc(N2CCOCC2)ccc1Nc1nc(NC2CCCCC2)c2cc[nH]c2n1	132000	318

2. Pharmacophore models

In this chapter, the pharmacophore models of the optimized models in complex with literature-known Janus kinase inhibitors from the respective training set are presented.

2.1 JAK1 MODELS

2.1.2 JAK1_SB2

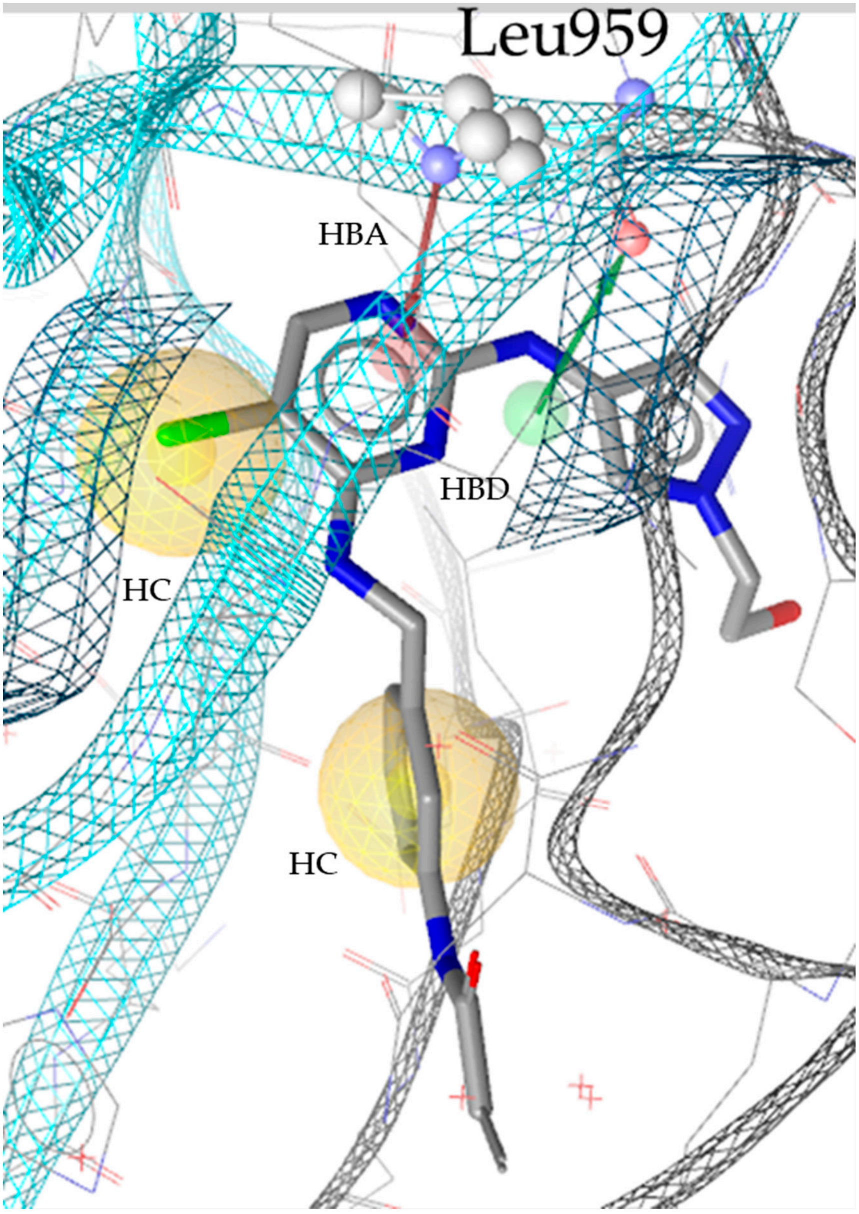

Figure S1. shows key interactions and structural features derived from a structure-based approach in complex with the co-crystallized ligand **SC1**. This model includes one HBA interaction and one HBD interaction with Leu959, two HCs, and 62 Xvols. This model was generated based on the X-ray coordinates of PDB: 6SMB. It includes three HBAs, two AIs, and 47 Xvols. Chemical features are color-coded: HBDs – green, HBAs – red, HCs – yellow, AIs – blue.

2.1.3 JAK1_SB3

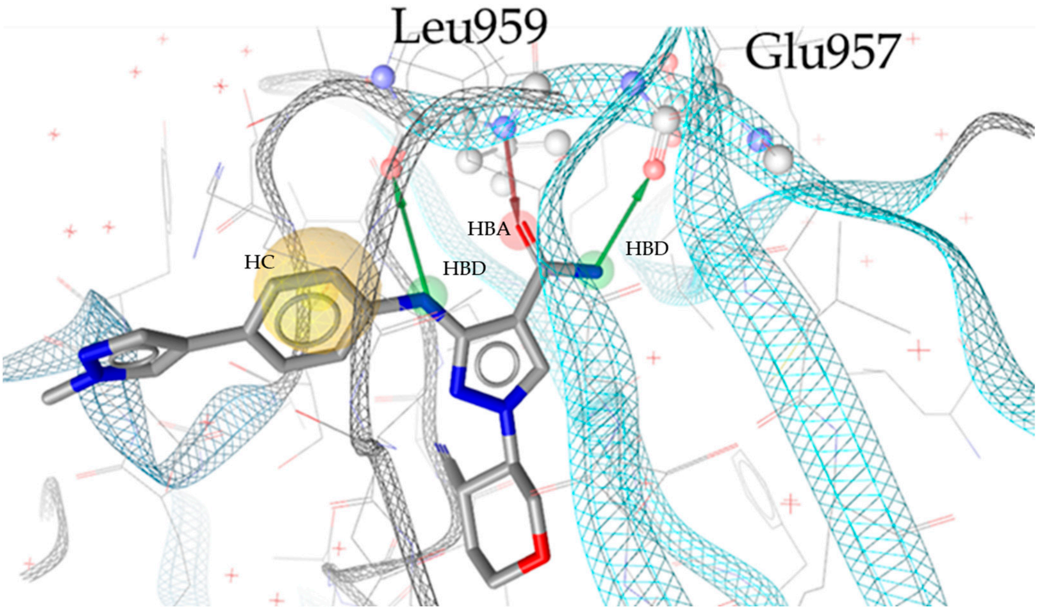

Figure S2. shows key interactions and structural features derived from a structure-based approach in complex with the co-crystallized ligand **SC2**. This model includes one HBD interaction and one HBA interaction with Leu959, one HBD interaction with Glu957, one HC, and 63 Xvols. This model was generated based on the X-ray coordinates of PDB: 5WO4.

2.1.1 JAK1_SB4

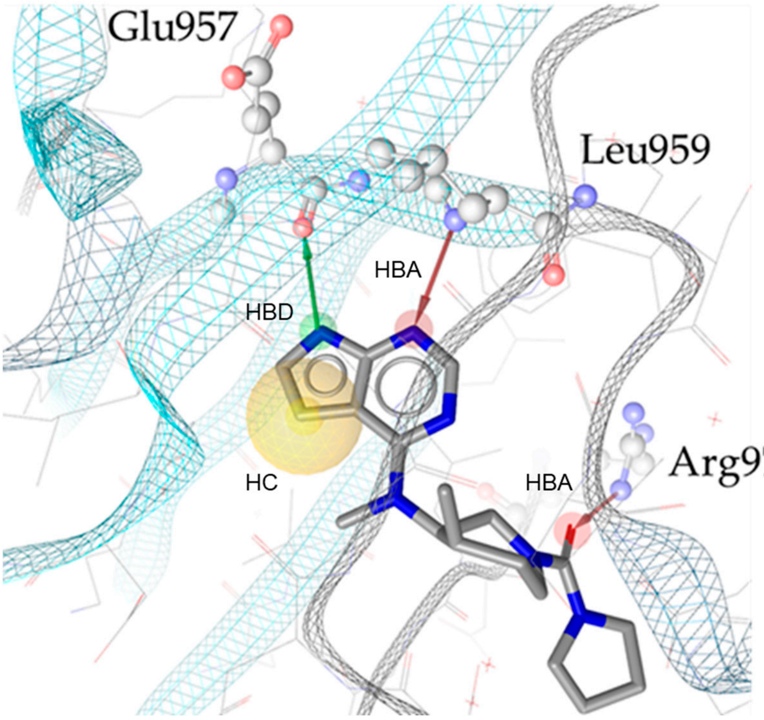

Figure S3. shows JAK1_SB1 in complex with the co-crystallized ligand **SC3**. This model includes one HBD interaction and one HBA interaction with Glu957, one HBA directed to Arg978, one HC, and 46 Xvols. This model was generated based on the X-ray coordinates of PDB: 4FK6.

2.1.6 JAK1_LB2

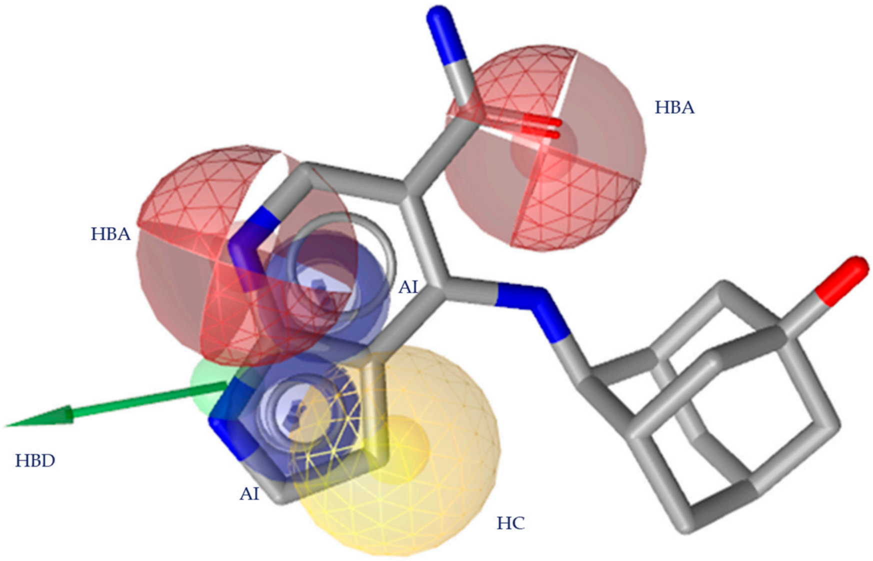

Figure S4 shows the optimized ligand-based (LB) pharmacophore model in complex with ligand **SC4**. This model was generated through alignment and merging of features from 4, **SC5**, and **SC6**. JAK1_LB2 includes one HBD, two HBAs, two AIs, one HC, and 58 Xvols.

2.1.5 JAK1_LB3

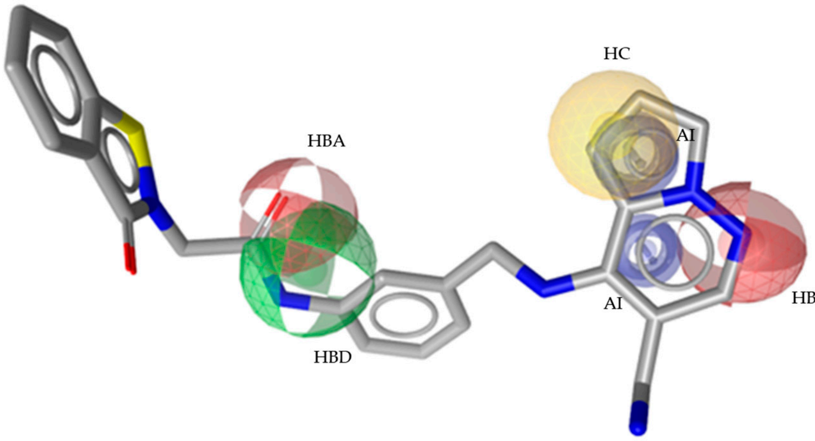

Figure S5 shows the optimized ligand-based (LB) pharmacophore model JAK1_LB3 in complex with ligand **SC7**. This model was generated through alignment and merging of features from **SC7** and **SC8**. JAK1_LB3 includes one HBD, two HBAs, two AIs, one HC, and 36 Xvols.

2.1.8 JAK1_LB4

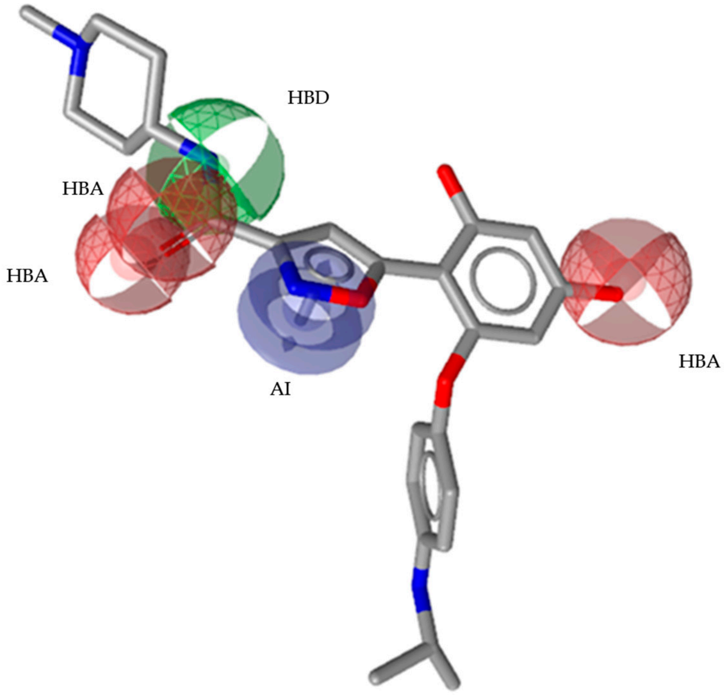

Figure S6 shows the optimized ligand-based (LB) pharmacophore model JAK1_LB4 in complex with ligand **SC9**. This model was generated through alignment and merging of features from **SC10**, **SC11**, and **SC12**. JAK1_LB4 includes one HBD, three HBAs, one AI, and 70 Xvols.

2.2 JAK2 MODELS

2.2.2 JAK2_SB2

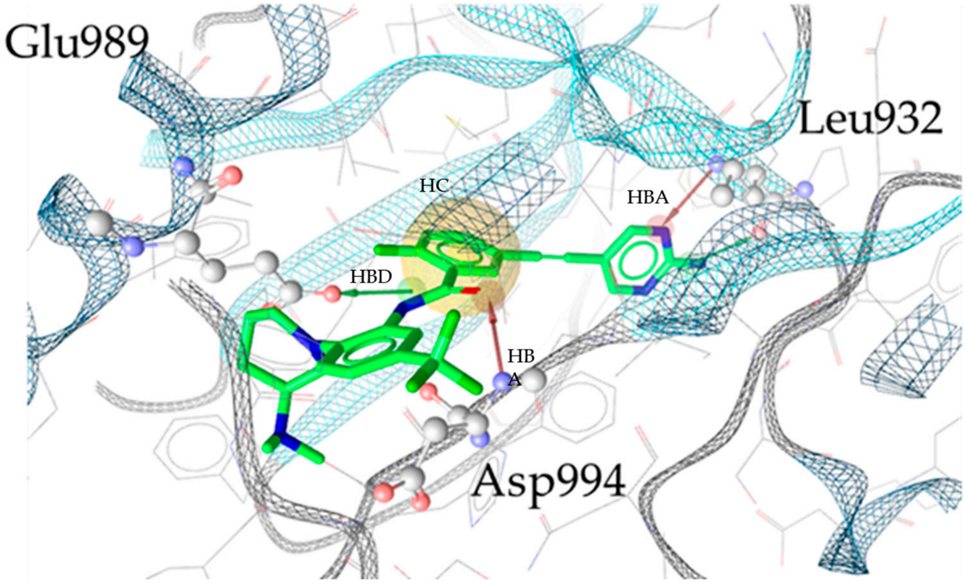

Figure S7. shows key interactions and structural features derived from a structure-based approach in complex with the co-crystallized ligand **SC13**. This model includes one HBD interaction with Glu989, two HBA interactions with Leu932 and Asp994, one HC, and thirteen Xvols. This model was generated based on the X-ray coordinates of PDB: 7TEU.

2.2.4 JAK2_LB2

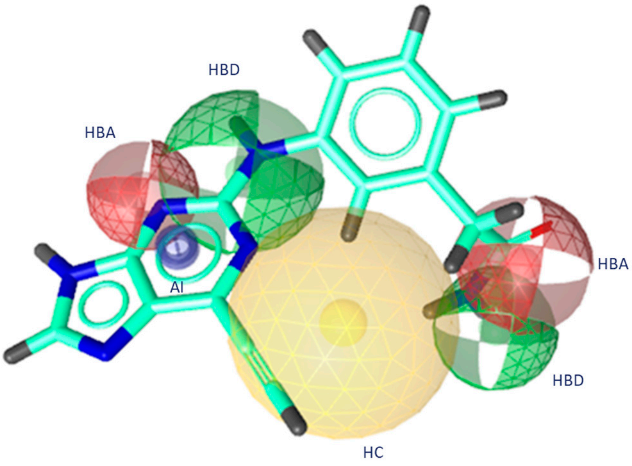

Figure S8. shows the optimized ligand-based (LB) pharmacophore model in complex with ligand **SC14**. This model was generated through alignment and merging of features from **SC15**, **SC14**, **10**, and **7**. JAK2_LB2 includes two HBDs, two HBAs, one HC, one AI, and 25 Xvols.

2.2.5 JAK2_LB3

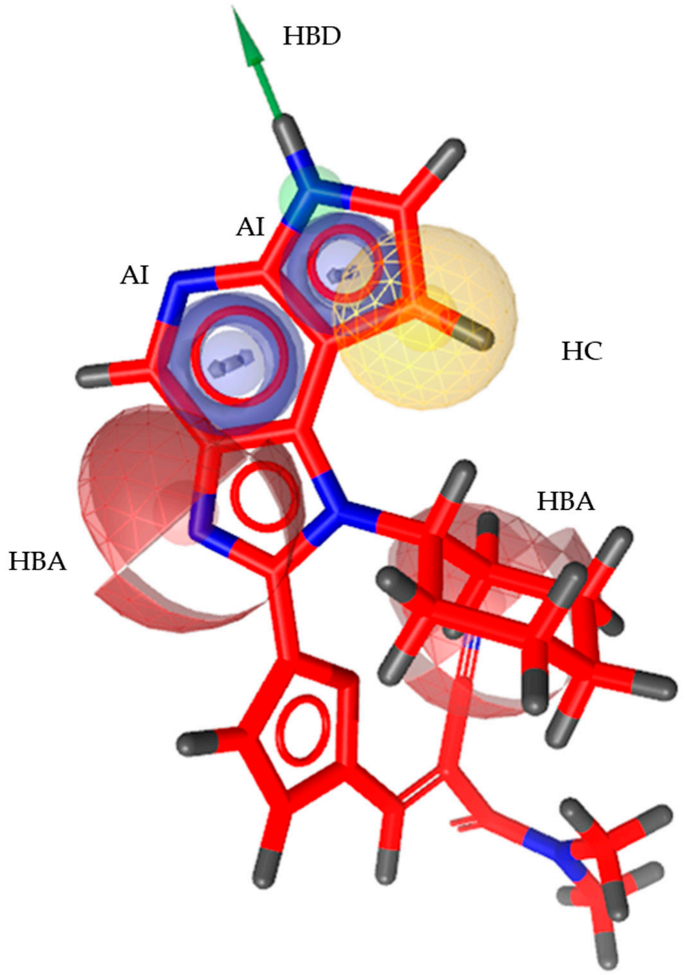

Figure S9. shows the optimized ligand-based (LB) pharmacophore model in complex with ligand **SC16**. This model was generated through alignment and merging of features from **SC17**, **SC16**, and **SC18**. JAK2_LB3 includes one HBD, two HBAs, two AIs, one HC, and one Xvol.

2.2.6 JAK2_LB4

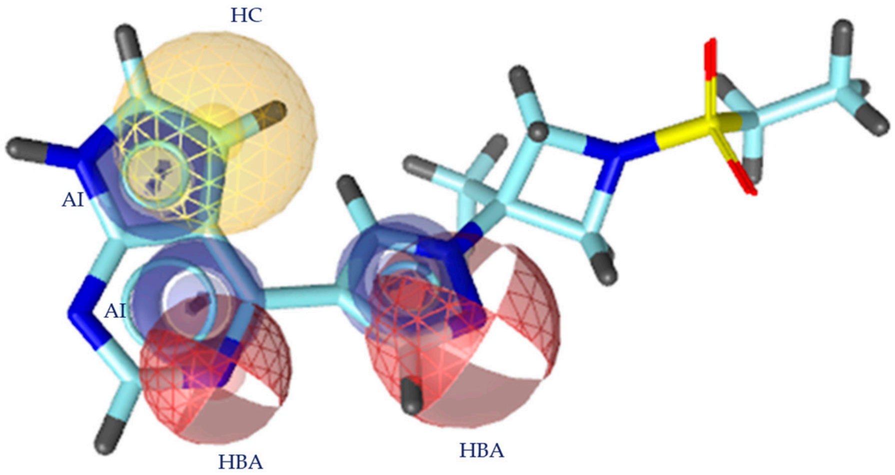

Figure S10. shows the optimized ligand-based (LB) pharmacophore model in complex with ligand **SC18**. This model was generated through alignment and merging of features from **SC19** and **SC20**. JAK2_LB4 includes two HBAs, three AIs, one HC, and two Xvols.

2.2.7 JAK2_LB5

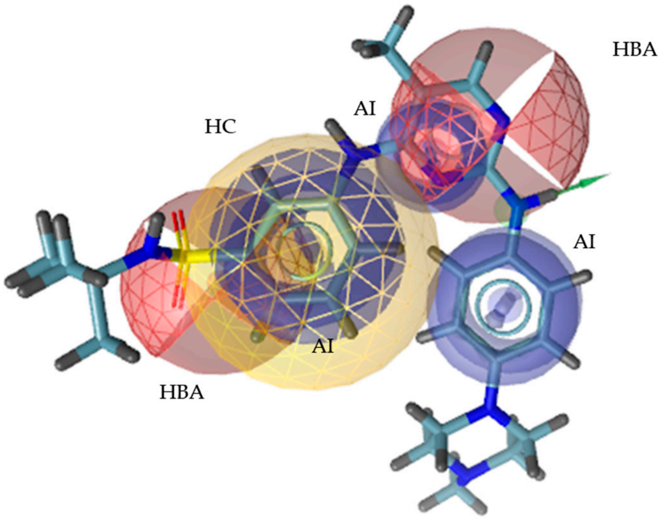

Figure S11. shows the optimized ligand-based (LB) pharmacophore model in complex with ligand **SC21**. This model was generated through alignment and merging of features from **SC22** and **SC23**. JAK2_LB5 includes one HBD, two HBAs, one HC, three AIs, and three Xvols.

2.2.8 JAK2_LB6

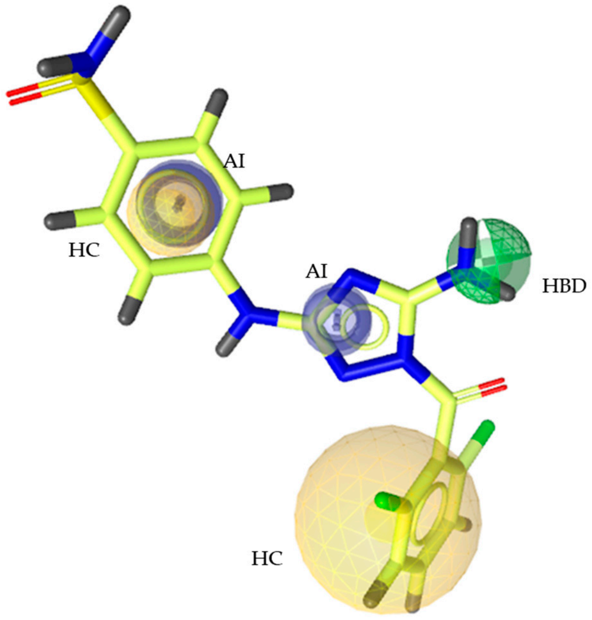

Figure S12. shows the optimized ligand-based (LB) pharmacophore model in complex with ligand **SC24**. This model was generated through alignment and merging of features from **SC25** and **SC26**. JAK2_LB6 includes one HBD, two AIs, two HCs, and six Xvols.

2.2.9 JAK2_LB7

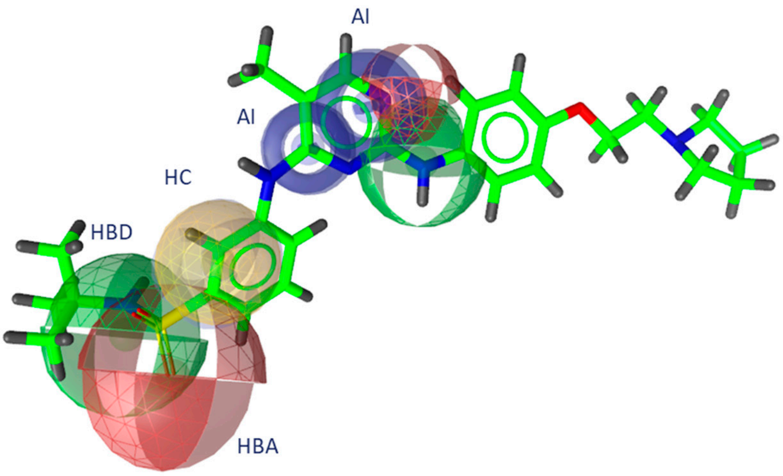

Figure S13. shows the optimized ligand-based (LB) pharmacophore model in complex with ligand **SC27**. This model was generated through alignment and merging of features from **SC28** and **SC29**. JAK2_LB7 includes two HBDs, two HBAs, one HC, two AIs, and four Xvols.

2.2.10 JAK2_LB8

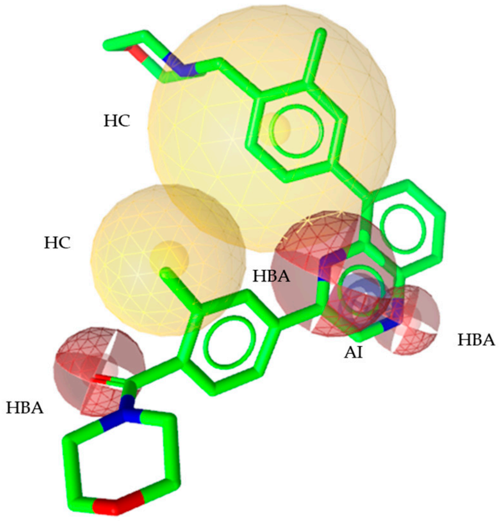

Figure S14. shows the optimized ligand-based (LB) pharmacophore model in complex with ligand **SC30**. This model was generated through alignment and merging of features from **SC30**, **SC11**, and **SC31**. JAK2_LB8 includes three HBAs, one AI, two HCs, and 17 Xvols.

2.3 JAK3 MODELS

2.3.1 JAK3_SB2

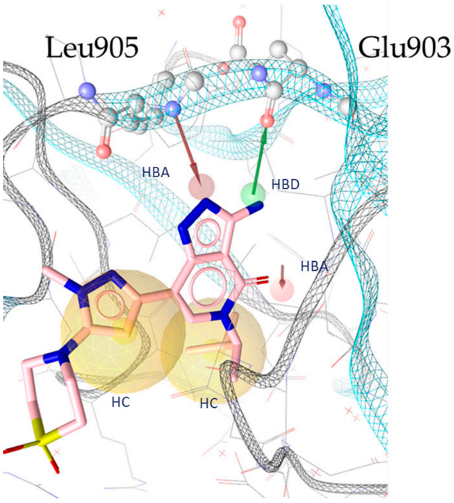

Figure S15. shows key interactions and structural features derived from a structure-based approach in complex with the co-crystallized ligand **SC32**. This model includes one HBD interaction with Glu903, two HBA interactions with Leu905 and H_2O, two HCs, and nineteen Xvols. This model was generated based on the X-ray coordinates of PDB: 3ZEP.

2.3.3 JAK3_SB3

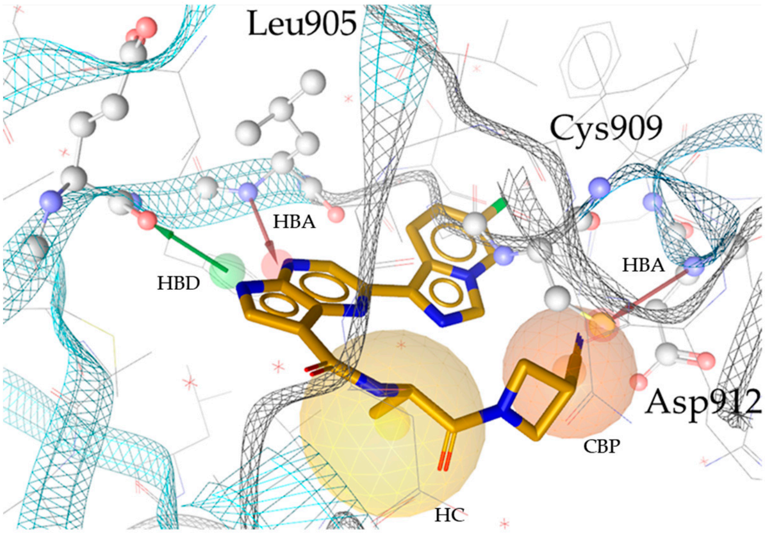

Figure S16. shows key interactions and structural features derived from a structure-based approach in complex with the co-crystallized ligand **SC33**. This model includes one HBD interaction with Glu903, two HBA interactions with Leu905 and Asp912, one RBP, one HC, and ten Xvols. This model was generated based on the X-ray coordinates of PDB: 5TTV.

2.3.4 JAK3_SB4

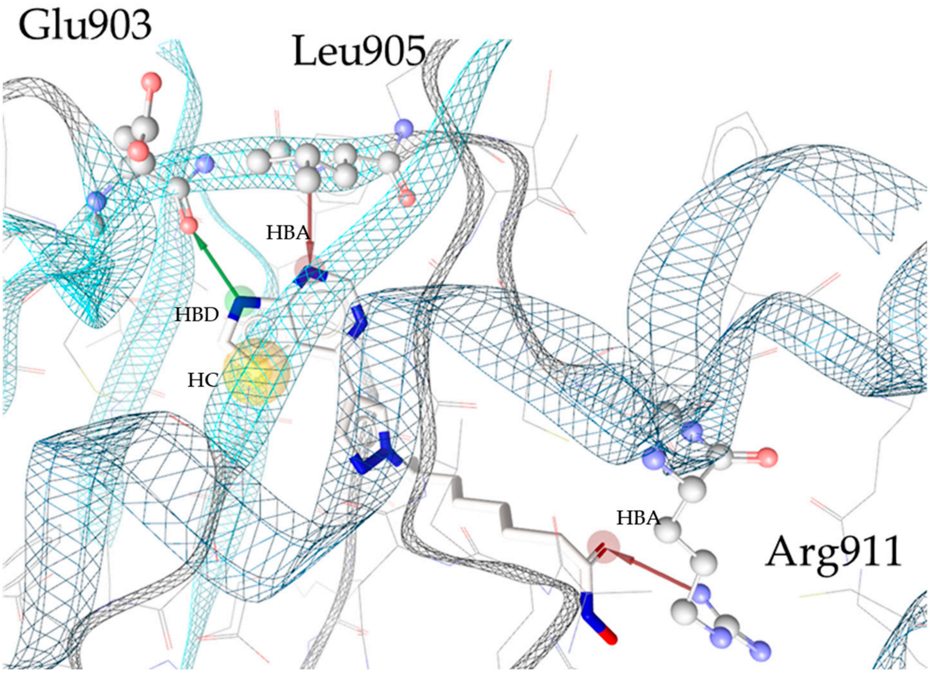

Figure 17. shows key interactions and structural features derived from a structure-based approach in complex with the co-crystallized ligand **SC34**. This model includes one HBD interaction with Glu903, two HBA interactions with Leu905 and Arg911, one HC, and nine Xvols. This model was generated based on the X-ray coordinates of PDB: 5LWM.

2.3.6 JAK3_LB2

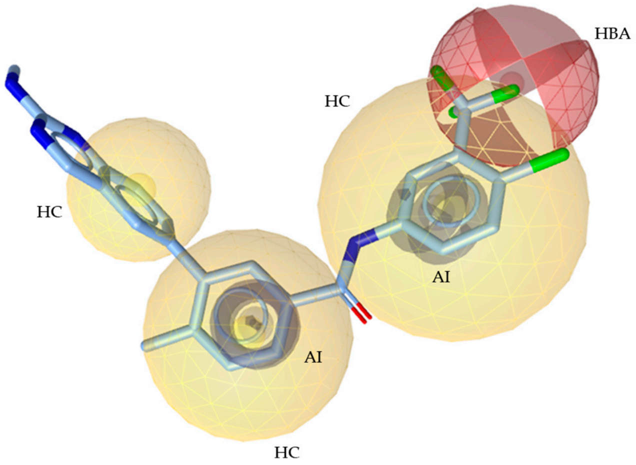

Figure S18. shows the optimized ligand-based (LB) pharmacophore model JAK3_LB2 in complex with ligand **SC35**. This model was generated through alignment and merging of features from **SC36**, **SC37**, and **SC38**. JAK3_LB2 includes one HBA, two AIs, three HCs, and 32 Xvols.

2.3.7 JAK3_LB3

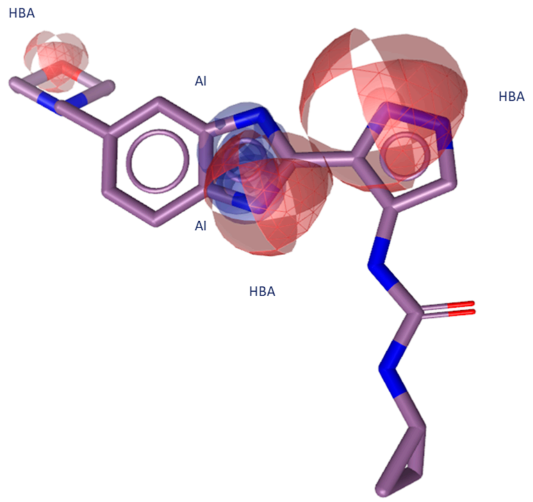

Figure S19. shows the optimized ligand-based (LB) pharmacophore model JAK3_LB3 in complex with ligand **SC37**. This model was generated through alignment and merging of features from **SC38**, **SC39**, **SC40**, and **12**. JAK3_LB3 includes three HBAs, two AIs, and 16 Xvols.

2.3.8 JAK3_LB4

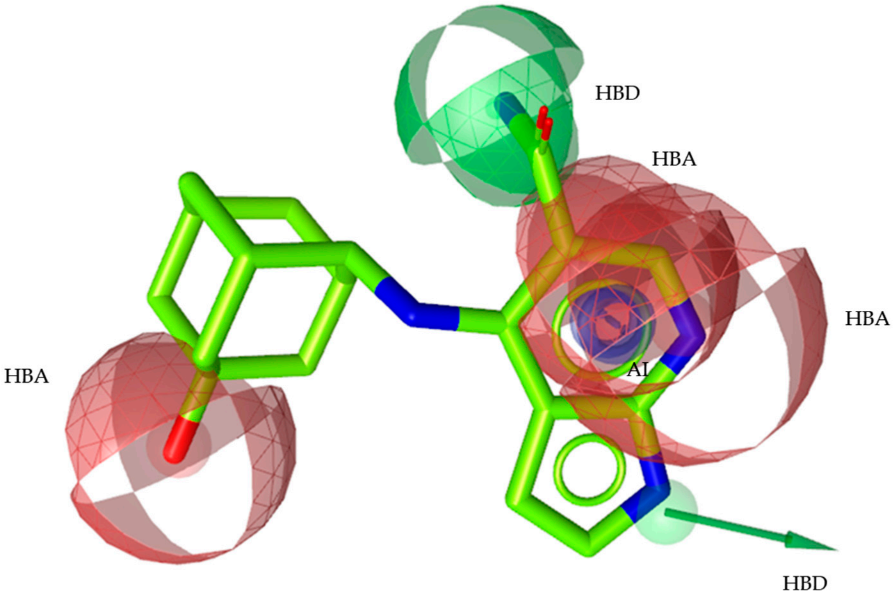

Figure S20. shows the optimized ligand-based (LB) pharmacophore model JAK3_LB4 in complex with ligand **SC41** This model was generated through alignment and merging of features from **SC41** and **SC42**. JAK3_LB4 includes two HBDs, three HBAs, one AI, and 22 Xvols.

2.3.9 JAK3_LB5

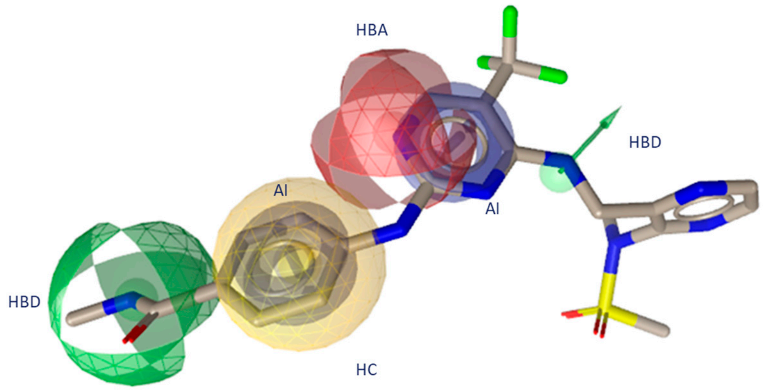

Figure S21. shows the optimized ligand-based (LB) pharmacophore model JAK3_LB5 in complex with ligand **SC43**. This model was generated through alignment and merging of features from **12** and **SC44**. JAK3_LB5 includes two HBDs, one HBA, two AIs, and 35 Xvols.

2.3.10 JAK3_LB6

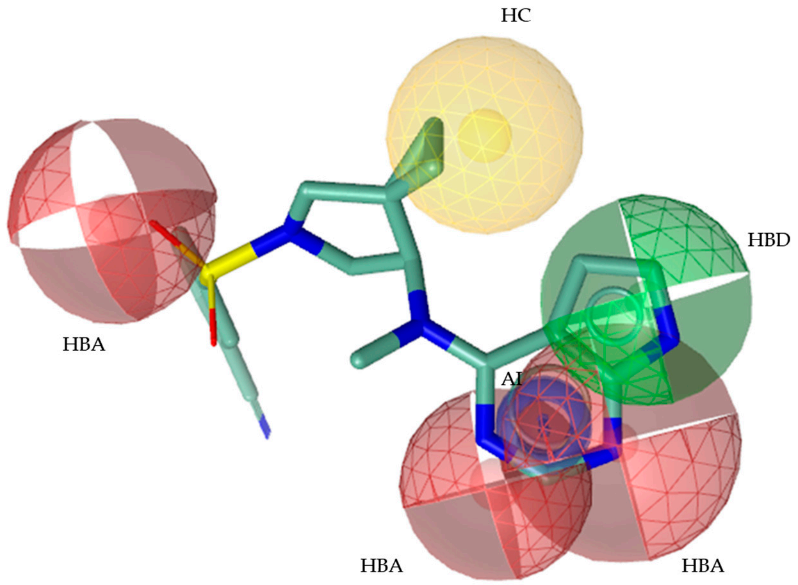

Figure S22. shows model JAK3_LB6 in complex with ligand **SC45**. This model was generated through alignment and merging of features from **SC46**, **SC47**, and **SC48**. It consists of one HBD, three HBAs, one AI, one HC, and 31 Xvols.

2.4 TYK2 MODELS

2.4.2 TYK2_SB2

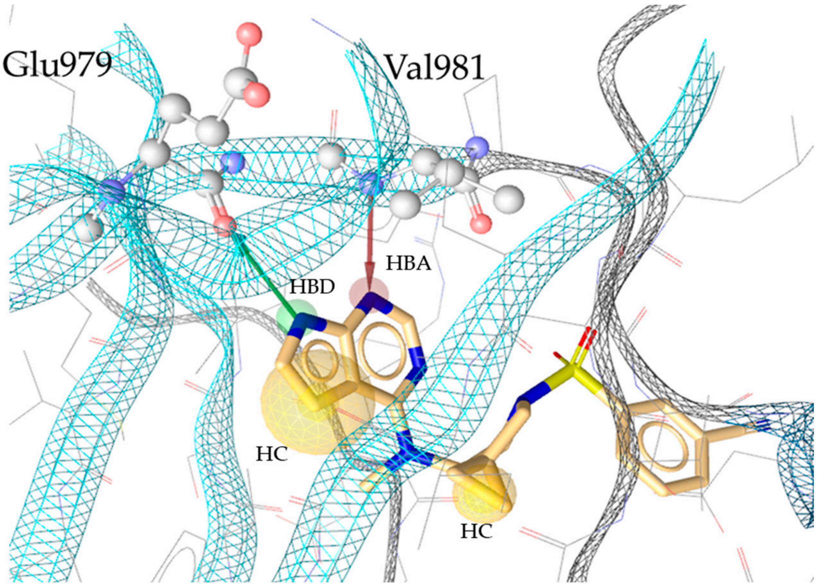

Figure S23. shows TYK2_SB2, illustrating key interactions and structural features derived from a structure-based approach in complex with the co-crystallized ligand **SC45**. This model includes one HBD interaction with Glu979, one HBA interaction with Val981, two HCs shown as yellow spheres, and 21 Xvols. This model was generated based on the X-ray coordinates of PDB: 3LXN.

2.4.3 TYK2_SB3

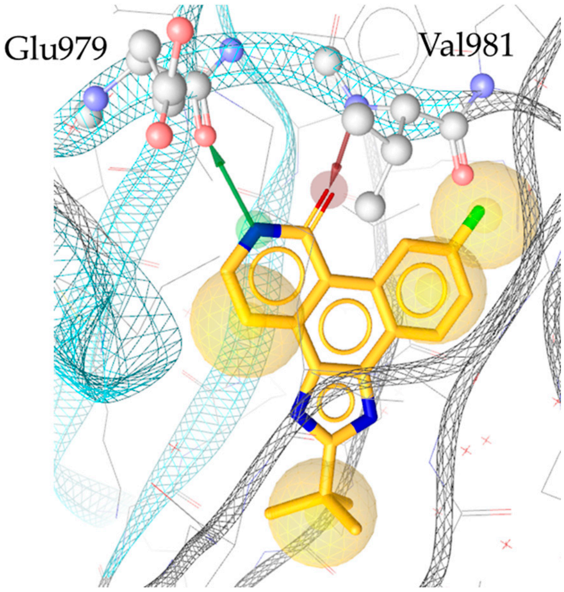

Figure S24. shows TYK2_SB3, illustrating key interactions and structural features derived from a structure-based approach in complex with the co-crystallized ligand **SC46**. This model includes one HBD interaction with Glu979, one HBA interaction with Val981, four HCs, and 30 Xvols. This model was generated based on the X-ray coordinates of PDB: 3NZ0.

2.4.4 TYK2_LB2

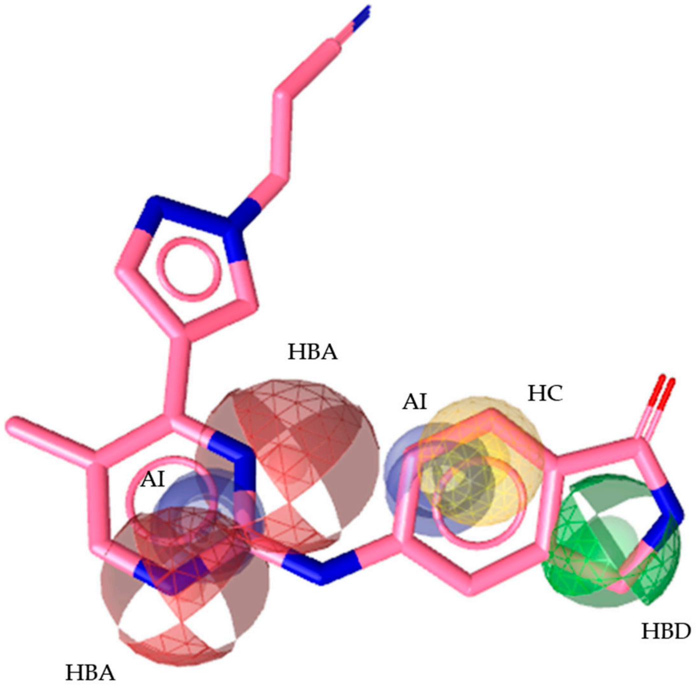

Figure S25. shows model TYK2_LB2 in complex with ligand **SC47**. This model was generated through alignment and merging of features from **SC48** and **SC15**. It consists of one HBD, two HBAs, one HC, two AIs, and 31 Xvols.

2.4.5 TYK2_LB3

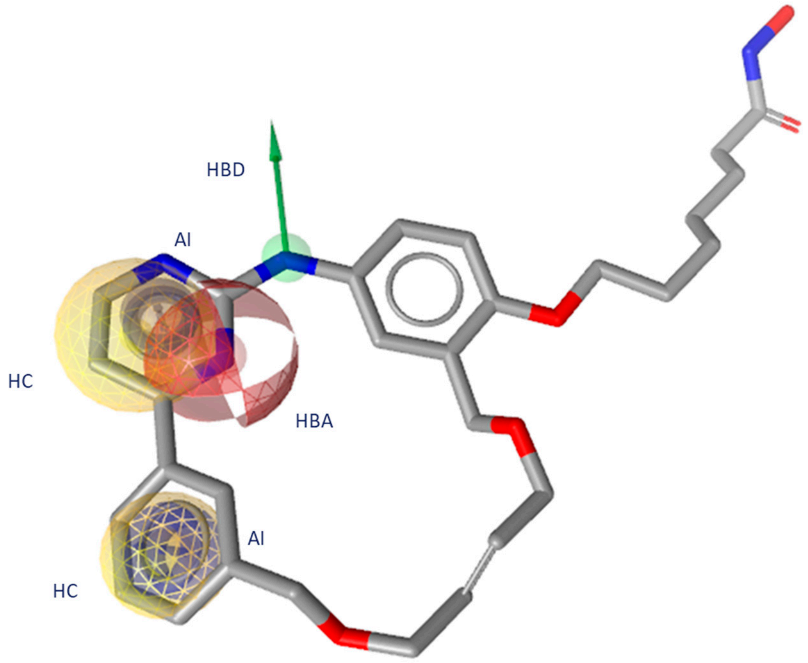

Figure S26. shows model TYK2_LB3 in complex with ligand **SC49**. This model was generated through alignment and merging of features from **SC50**, **SC51**, and **SC49**. It consists of one HBD, one HBA, two AIs, two HCs, and 42 Xvols.

2.4.6 TYK2_LB4

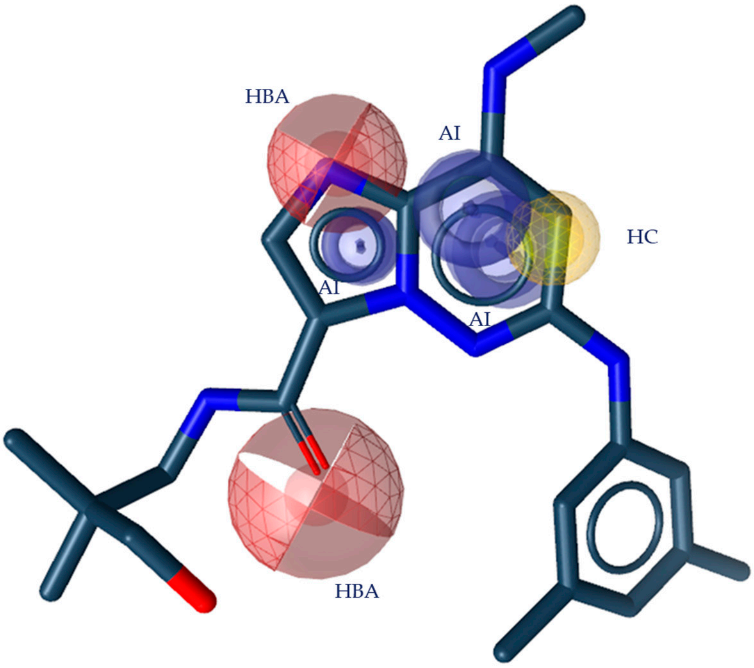

Figure S27. shows model TYK2_LB4 in complex with ligand **SC52**. This model was generated through alignment and merging of features from **SC53**, **SC54**, and **SC52**. It consists of two HBAs, three AIs, one HC, and 46 Xvols.

2.4.7 TYK2_LB5

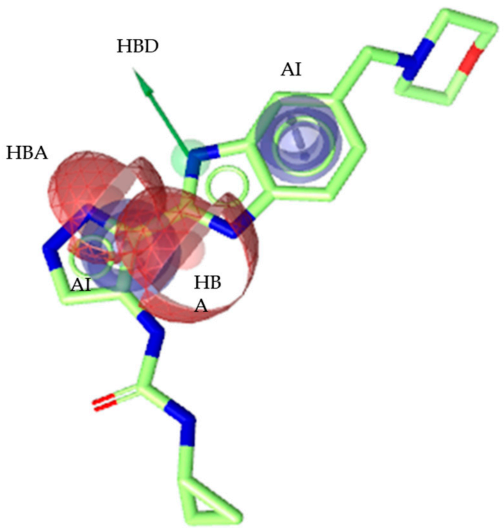

Figure S28. shows model TYK2_LB5 in complex with ligand **SC39**. This model was generated through alignment and merging of features from **SC55**, **SC56**, and **SC57**. TYK2_LB5 includes one HBD, two HBAs, two AIs, and 43 Xvols.

2.4.9 TYK2_LB6

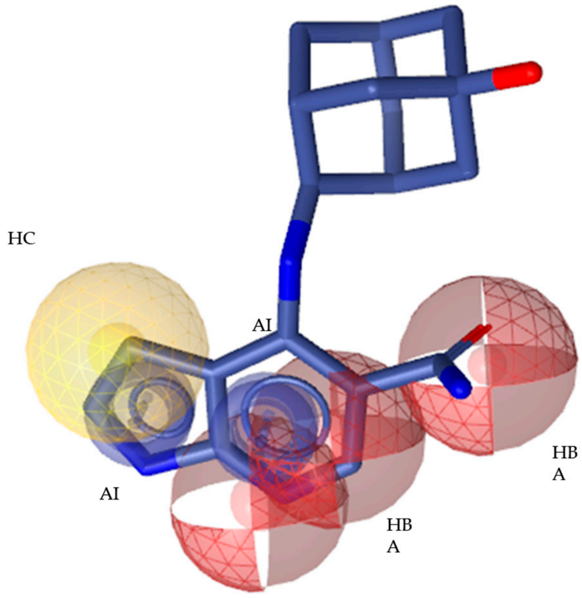

Figure S29. shows model TYK2_LB6 in complex with ligand **SC41**. This model was generated through alignment and merging of features from **SC58**, **SC59**, and **SC60**. It consists of three HBAs, two AIs, one HC, and 42 Xvols.

2.5 Pharmacophore features

Table S9. provides an overview of the models and the number of pharmacophore features, including hydrogen bond acceptors (HBAs), hydrogen bond donors (HBDs), aromatic interactions (AIs), hydrophobic contacts (HCs), covalent contacts (CCs), and exclusion volumes (Xvols).

MODEL	PDB	HBA	HBD	AR	HC	CC	Xvols
JAK1_SB1	5HX8	1	1		2		66
JAK1_SB2	6SMB	1	1		2		62
JAK1_SB3	5WO4	1	2		1		63
JAK1_SB4	4FK6	2	1		1		46
JAK1_LB1		3		2			47
JAK1_LB2		2	1	2	1		58
JAK1_LB3		2	1	2	1		36
JAK1_LB4		3	1	1			70
JAK2_SB1	6VNB	1	1		2		9
JAK2_SB2	7TEU	2	2		1		13
JAK2_LB1		2	1	2			9
JAK2_LB2		2	2	1	1		25
JAK2_LB3		2	1	2	1		1
JAK2_LB4		2		3	1		2
JAK2_LB5		2	1	3	1		3
JAK2_LB6			1	2	2		6
JAK2_LB7		2	2	2	1		4
JAK2_LB8		3		1	2		17
JAK3_SB1	4Z16	1	1		3		20
JAK3_SB2	3ZEP	2	1		2		19
JAK3_SB3	5TTV	2	1		1	1	10
JAK3_SB4	5LWM	2	1		1		9
JAK3_LB1		1		2	2		29
JAK3_LB2		1		2	3		32
JAK3_LB3		1		2	3		16
JAK3_LB4		3	2	1			22
JAK3_LB5		1	2	2	1		35
JAK3_LB6		3	1	1	1		32
TYK2_SB1	6VNS	3	1		2		26
TYK2_SB2	3LXN	1	1		2		21
TYK2_SB3	3NZ0	1	1		4		30
TYK2_LB1		2	1	1	1		35
TYK2_LB2		1	1	2	2		42
TYK2_LB3		2		3	1		46

TYK2_LB4		2	1	2	1		31
TYK2_LB5		2	1	2			43
TYK2_LB6		3	2		1		42

2.6. Amino acid interactions of the structure-based models

Table S10. shows the amino acid interaction profile of the structure-based pharmacophore models as displayed in LigandScout 4.4.5, including hydrogen bond acceptors (HBAs), hydrogen bond donors (HBDs), aromatic interactions (AIs), hydrophobic contacts (HCs), covalent contacts (CCs), and exclusion volumes (Xvols).

	**JAK1_SB1**	**JAK1_SB2**	**JAK1_SB3**	**JAK1_SB4**
**PDB**	5HX8	6SMB	5WO4	4FK6
**Resolution (Å)**	2,2	2,04	1,84	2,2
**Mutation**	NO	NO	NO	NO
LEU881	HC	HC	HC	HC
LEU959	HBA	HBA +HBD	HBA +HBD	HBA
VAL889	HC	HC		HC
ARG879				HBA
ALA906	HC	HC		HC
VAL938	HC	HC		HC
MET956	HC	HC		HC
GLU957	HBD		HBD	HBD
PHE958			HC	
LEU1010	HC	HC		HC
	**JAK2_SB1**	**JAK2_SB2**		
**PDB**	4FK6	7TEU		
**Resolution (Å)**	2,19	1,45		
**Mutation**	NO	YES		
LEU855	HC			
VAL863	HC			
ALA880	HC			
GLU898		HBD		
VAL911	HC	HC		
GLU930	HBD			
MET929	HC	HC		
TYR931	HC			
LEU932	HBA	HBA +HBD		
LEU983	HC			
ASP994		HBA		

	**JAK3_SB1**	**JAK3_SB2**	**JAK3_SB3**	**JAK3_SB4**
PDB	4Z16	3ZEP	5TTV	5LWM
Resolution (Å)	2.9	2.35	1.93	1.55
Mutation	NO	YES	YES	NO
LEU828	HC	HC	HC	
ALA853	HC		HC	HC
VAL836	HC	HC	HC	HC
VAL884			HC	HC
MET902	HC		HC	HC
GLU903		HBD	HC	HBD
ARG911				HBA
ASP912			HBA	
LEU905	HBA +HBD	HBA	CBP	HBA
LEU956	HC	HC	HC	HC
	**TYK2_SB1**	**TYK2_SB2**	**TYK2_SB3**	
**PDB**	6VNS	3LXN	3NZ0	
**Resolution (Å)**	2.09	2.5	2	
**Mutation**	YES	YES	YES	
LEU903			HC	
VAL911	HC	HC	HC	
ALA928	HC	HC	HC	
ILE960	HC	HC	HC	
MET978	HC	HC	HC	
GLU979		HBD	HBD	
TYR980			HC	
VAL981	HBA +HBD	HBA	HBA	
SER985	HBA	HC	HC	

3. Theoretical evaluation

In the following **Table S11 -14**, the theoretical evaluation of each model of JAK1, JAK2, JAK3, and TYK2 is shown, focusing on model accuracy, enrichment factor (EF), and yield of actives (YoA). The analysis considered true positives (TPs), false positives (FPs), true negatives (TNs), and false negatives (FNs). Additionally, the dataset sizes, including active compounds (ACs), inactive compounds (IAs), and decoys (DCs), are shown. Furthermore, the corresponding Receiver Operating Characteristic (ROC) curves are presented in Figure S30- 33.

3.1 JAK1

3.1.1 Results of the theoretical evaluation process (JAK1)

Table S11 shows the results of the theoretical evaluation of JAK1

**model intern ID**	**JAK1_SB 1**	**JAK1_SB 2**	**JAK1_SB 3**	**JAK1_SB 4**	**JAK1_LB 1**	**JAK1_LB 2**	**JAK1_LB 3**	**JAK1_LB 4**
# actives	13	12	10	23	22	20	11	20
# inactives	0	0	0	0	0	0	0	4
# decoys	9	9	4	6	11	10	14	20
True Positives	13	12	10	23	22	20	11	20
False Positives	9	9	4	6	11	10	14	24
True Negatives	3302	3302	3307	3305	3300	3301	3297	3287
# of actives in the database	105	105	105	105	105	105	105	105
# of inactives in the database	48	48	48	48	48	48	48	48
# of decoys in the database	3263	3263	3263	3263	3263	3263	3263	3263
Model Accuracy	0.97	0.97	0.97	0.97	0.97	0.97	0.97	0.97
Yield of Actives	0.59	0.57	0.71	0.79	0.67	0.67	0.44	0.45
Enrichment Factor	19.22	18.59	23.24	25.80	21.69	21.69	14.31	14.79
sensitivity	0.12	0.11	0.10	0.22	0.21	0.19	0.10	0.19
specificity	0.9722	0.9719	0.9713	0.9752	0.9749	0.9743	0.9716	0.9743

3.1.2 ROC-curves JAK1

These curves demonstrate the model performance of JAK1 models in terms of true positive rate (sensitivity) versus false positive rate (1-specificity) for each pharmacophore model.

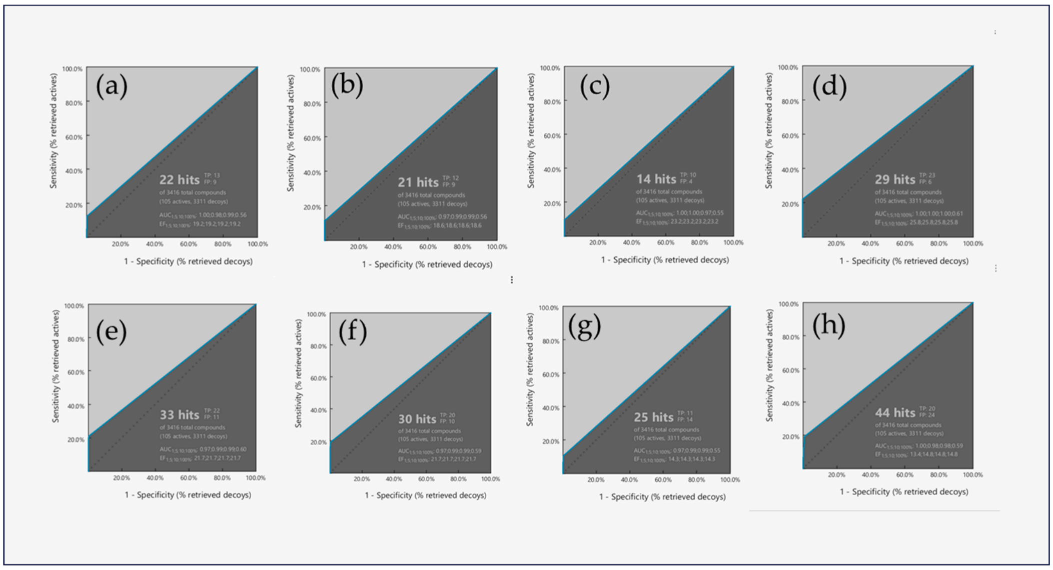

Figure S30. Receiver Operating Characteristic (ROC) curves of the individual pharmacophore models: (a) JAK1_SB1. (b) JAK1_SB2. (c) JAK1_SB3. (d) JAK1_SB4. (e) JAK1_LB1. (f) JAK1_LB2. (g) JAK1_LB3. and (h) JAK1_LB4.

3.2 *JAK2*

3.2.1 Results of the theoretical evaluation process (JAK2)

Table S12 shows the results of the theoretical evaluation of JAK2

model intern ID	JAK2_SB1	JAK2_SB2	JAK2_LB1	JAK2_LB2	JAK2_LB3	JAK2_LB4	JAK2_LB5	JAK2_LB6	JAK2_LB7	JAK2_LB8
# actives	20	13	77	17	31	27	49	15	26	32
# inactives	3	0	9	1	0	3	5	1	3	4
# decoys	2	10	29	4	1	5	8	8	4	8
TPs	20	13	77	17	31	27	49	15	26	32
FPs	5	10	38	5	1	8	13	9	7	12
TNs	2884	2879	2851	2884	2888	2881	2876	2880	2882	2877
# of ACs in the database	185	185	185	185	185	185	185	185	185	185
# of IAs in the database	49	49	49	49	49	49	49	49	49	49
# of DCs in the database	2840	2840	2840	2840	2840	2840	2840	2840	2840	2840
Model Accuracy	0.94	0.94	0.95	0.94	0.95	0.95	0.95	0.94	0.95	0.95
YoA	0.80	0.57	0.67	0.77	0.97	0.77	0.79	0.63	0.79	0.73
EF	13.29	9.39	11.13	12.84	16.10	12.82	13.13	10.39	13.09	12.08
sensitivity	0.11	0.07	0.42	0.09	0.17	0.15	0.26	0.08	0.14	0.17
specificity	0.94	0.94	0.96	0.94	0.95	0.95	0.95	0.94	0.94	0.95

3.2.2 ROC-curves JAK2

These curves demonstrate the model performance of JAK2 models in terms of true positive rate (sensitivity) versus false positive rate (1-specificity) for each pharmacophore model.

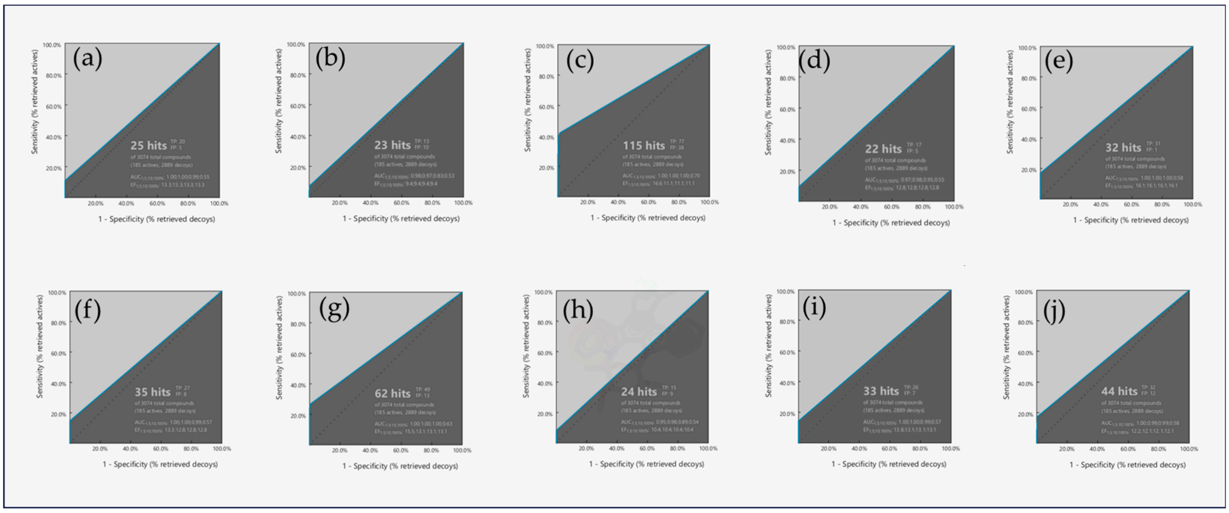

Figure S31. Receiver Operating Characteristic (ROC) curves of the individual pharmacophore models: (a) JAK2_SB1. (b) JAK2_SB2. (c) JAK2_LB1. (d) JAK2_LB2. (e) JAK2_LB3. (f) JAK2_LB4. (g) JAK2_LB5. (h) JAK2_LB6. (i) JAK2_LB7. and (j) JAK2_LB8.

3.3. *JAK3*

3.3.1 Results of the theoretical evaluation process (JAK3)

Table S13 shows the results of the theoretical evaluation of JAK3

model intern ID	JAK3_SB1	JAK3_SB2	JAK3_SB3	JAK3_SB4	JAK3_LB1	JAK3_LB2	JAK3_LB3	JAK3_LB4	JAK3_LB5	JAK3_LB6
# actives	15	9	37	25	31	13	32	20	17	57
# inactives	0	1	2	3	2	2	1	2	0	2
# decoys	8	22	30	28	51	29	51	17	3	59
True Positives	15	9	37	25	31	13	32	20	17	57
False Positives	8	23	32	31	53	31	52	19	3	61
True Negatives	4533	4518	4509	4510	4488	4510	4489	4522	4538	4480
# of actives in the database	129	129	129	129	129	129	129	129	129	129
# of inactives in the database	42	42	42	42	42	42	42	42	42	42
# of decoys in the database	4499	4499	4499	4499	4499	4499	4499	4499	4499	4499
Model Accuracy	0.97	0.97	0.97	0.97	0,97	0.97	0.97	0.97	0.98	0.97
Yield of Actives	0.65	0.28	0.54	0.45	0,37	0.30	0.38	0.51	0.85	0.48
Enrichment Factor	23.61	Okt.18	19.41	16.16	13,36	Okt.70	13.79	18.56	30.77	17.49
sensitivity	0.11	0.07	0.27	0.19	0,23	0.10	0.24	0.15	0.13	0.42
specificity	0.97	0.97	0.98	0.98	0,98	0.97	0.98	0.98	0.98	0.98

3.3.2 ROC-curves JAK3

These curves demonstrate the model performance of JAK3 models in terms of true positive rate (sensitivity) versus false positive rate (1-specificity) for each pharmacophore model

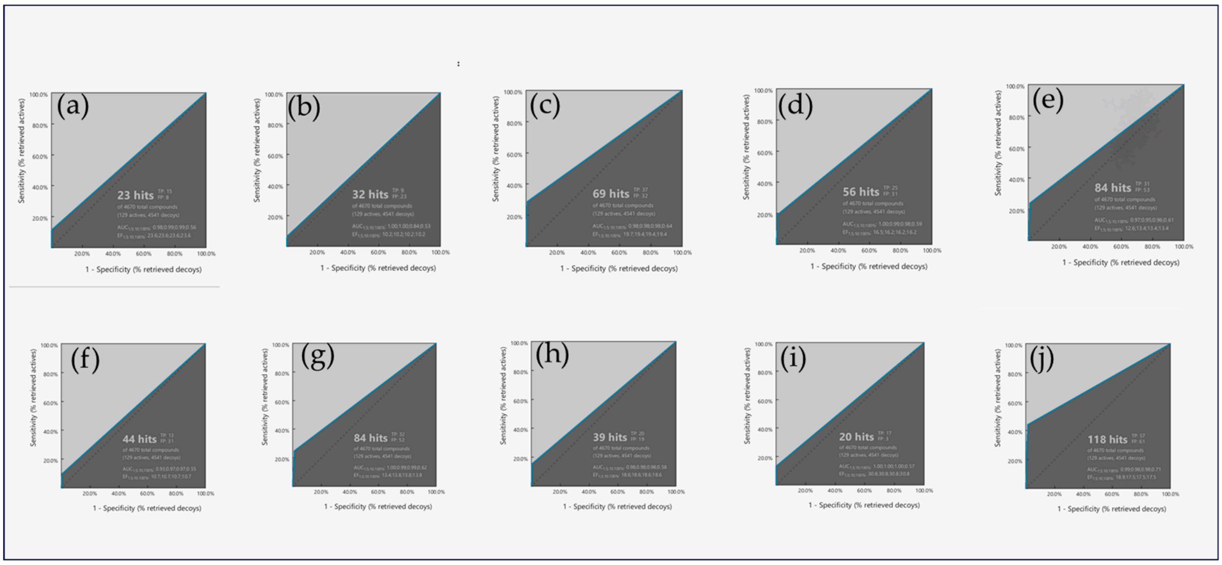

Figure S32. Receiver Operating Characteristic (ROC) curves of the individual pharmacophore models: (a) JAK3_SB1. (b) JAK3_SB2. (c) JAK3_SB3. (d) JAK3_SB4. (e) JAK3_LB1. (f) JAK3_LB2. (g) JAK3_LB3. (h) JAK3_LB4. (i) JAK3_LB5. and (j) JAK3_LB6.

3.4 TYK2

3.4.1 Results of the theoretical evaluation process (TYK2)

Table S14 shows the results of the theoretical evaluation of TYK2

model intern ID	TYK2_SB1	TYK2_SB2	TYK2_SB3	TYK2_LB1	TYK2_LB2	TYK2_LB3	TYK2_LB4	TYK2_LB5	TYK2_LB6
# actives	8	9	6	6	5	16	9	25	15
# inactives	2	22	0	14	0	2	0	1	6
# decoys	12	16	6	9	1	11	32	23	39
True Positives	8	9	6	6	5	16	9	25	15
False Positives	14	38	6	23	1	13	32	24	45
True Negatives	3097	3073	3105	3088	3110	3098	3079	3087	3066
# of actives in the database	75	75	75	75	75	75	75	75	75
# of inactives in the database	61	61	61	61	61	61	61	61	61
# of decoys in the database	3050	3050	3050	3050	3050	3050	3050	3050	3050
Model Accuracy	0.97	0.97	0.98	0.97	0.98	0.98	0.97	0.98	0.97
Yield of Actives	0.36	0.19	0.50	0.21	0.83	0.55	0.22	0.51	0.25
Enrichment Factor	15.45	8.13	21.24	8.79	35.40	23.44	9.32	21.67	10.62
sensitivity	0.11	0.12	0.08	0.08	0.07	0.21	0.12	0.33	0.20
specificity	0.98	0.98	0.98	0.98	0.98	0.98	0.98	0.98	0.98

3.4.2 ROC-curves TYK2

These curves demonstrate the model performance of TYK2 models in terms of true positive rate (sensitivity) versus false positive rate (1-specificity) for each pharmacophore model.

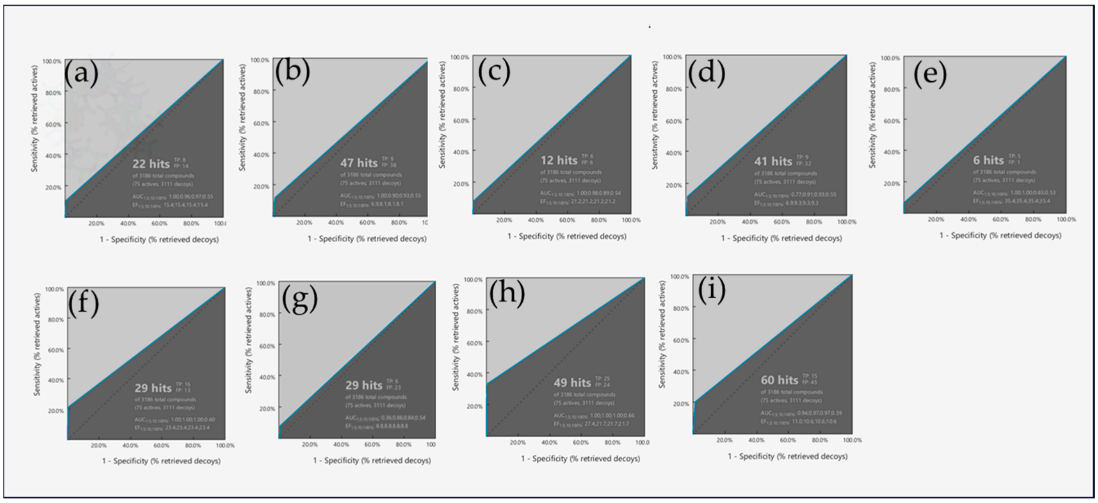

Figure S33. Receiver Operating Characteristic (ROC) curves of the individual pharmacophore models: (a) TYK2_SB1. (b) TYK2_SB2. (c) TYK2_SB3. (d) TYK2_LB1. (e) TYK2_LB2. (f) TYK2_LB3. (g) TYK2_LB4. (h) TYK2_LB5. and (i) TYK2_LB6.

4. Idetified Pesticides

In this chapter. the hit lists of the virtually identified pesticides are presented and assigned to the corresponding models.

4.1.JAK1 HITS

Table S15 shows all identified pesticides from JAK1 obtained through virtual screening of models against the LUXPEST database.

	JAK1_SB1	JAK1_LB4	JAK1_SB4	JAK1_LB3
2-Amino-4.6-dimethylpyrimidine			x	
3.5.6-Trichloro-2-pyridinol	x			
3.5-Dichloro-2.4-difluoroaniline			x	
Chlorpropham			x	
Cybutryne			x	
Deethylatrazine			x	
Deisopropylatrazine			x	
Desethylterbutylazine			x	
Diflufenican		x		
Florasulam				x
Novaluron		x		
Picolinafen		x		
Pymetrozine	x			

4.2 JAK2 HITS

Table S16 shows all identified pesticides from JAK2 obtained through virtual screening of models against the LUXPEST database.

	JAK2_LB1	JAK2_LB4	JAK2_LB8	JAK2_SB1
2-Hydroxyatrazine				x
4-((4.5-Dihydro-3-methoxy-4-methyl-5-oxo-1H-1.2.4-triazol-1-yl)carbonylsulfamoyl)-5-methyl-thiophene-3-carboxylic acid			x	
6-[2-[(E)-(5.6-Dihydro-1.4.2-dioxazin-3-yl)(methoxyimino)methyl]phenoxy]-5-fluoro-4(3H)-pyrimidinone			x	
Atrazine				x
Benzovindiflupyr	x			
Boscalid	x			
Chlorantraniliprole	x			
Cyanazine				x
Cyprodinil	x			
Diflufenican	x			
Fluconazole			x	
Flusilazole			x	
hifensulfuron methyl			x	
Iodosulfuron-methyl			x	
Isavuconazole			x	
Isoxaben	x			
Mepronil	x			
Metsulfuron-methyl			x	
Picolinafen	x			
Posaconazole			x	
Prosulfuron			x	
Pyrimethanil	x			
Ravuconazole			x	
Thiabendazole	x			
Triazoxide		x		
Tribenuron			x	
Tribenuron-methyl			x	
Voriconazole			x	

4.3 JAK3 HITS

Table S17 shows all identified pesticides from JAK3 obtained through virtual screening of models against the LUXPEST database.

	JAK3_LB 6	JAK3_SB 3	JAK3_SB 1	JAK3_LB 2	JAK3_LB 3	JAK3_LB 1	JAK3_SB 4	JAK3_LB 4
Diflufenican				X		X		
Topramezone	X							
Ravuconazole	X	X		X				
Florasulam					X			
Cybutryne			X					
Isavuconazole	X	X					X	
Sulfosulfuron					X			
Prosulfuron	X							
Cyprodinil			X			X		
Penoxsulam					X			
Mesosulfuron-methyl	X							
Benthiavalicarb-isopropyl							X	
Fluxapyroxad	X							
Atrazine			X					
Mesosulfuron	X							
Thiophanate-methyl		X						
Phenmedipham		X						
Terbutryn			X					
Pyrimethanil			X					
Mepanipyrim			X			X		
Fipronil amide								X
Cyanazine			X					
Picolinafen				X				

4.4 TYK2 HITS

Table S18 shows all identified pesticides from TYK2 obtained through virtual screening of models against the LUXPEST database.

	TYK2_LB 5	TYK2_LB 6	TYK2_LB 1	TYK2_SB 1	TYK2_LB 3	TYK2_SB 2	TYK2_SB 3
2-Hydroxyatrazine						X	
3.5-Dichloro-2.4-difluoroaniline							X
Atrazine						X	
Benomyl		X					
Benthiavalicarb		X					
Bixafen			X				
Bupirimate				X			
Cyanazine						X	
Cyprodinil	X						
Diflufenican			X				
Fenazaquin					X		
Flazasulfuron			X				
Florasulam						X	
Flufenoxuron							X
hifensulfuron methyl				X			
Iodosulfuron-methyl			X				
Isopyrazam				X			
Mesosulfuron			X				
Metosulam		X					
Proquinazid		X					
Pyrimethanil	X						
Pyroxsulam					X		
Sedaxane			X				
Sulfosulfuron		X					
Teflubenzuron				X			
Thiabendazole	X						
Triazoxide		X					

References

1. Macfarlane, E.; Carey, R.; Keegel, T.; El-Zaemay, S.; Fritschi, L. Dermal exposure associated with occupational end use of pesticides and the role of protective measures. Saf. Health Work. 2013, 4, 136–141. https://doi.org/10.1016/j.shaw.2013.07.004.
2. Zhang, X.; Wu, M.; Yao, H.; Yang, Y.; Cui, M.; Tu, Z.; Stallones, L.; Xiang, H. Pesticide poisoning and neurobehavioral function among farm workers in Jiangsu, People's Republic of China. Cortex 2016, 74, 396–404. https://doi.org/10.1016/j.cortex.2015.09.006.
3. Wang, L.; Liu, Z.; Zhang, J.; Wu, Y.; Sun, H. Chlorpyrifos exposure in farmers and urban adults: Metabolic characteristic, exposure estimation, and potential effect of oxidative damage. Environ. Res. 2016, 149, 164–170. https://doi.org/10.1016/j.envres.2016.05.011.
4. Damalas, C.A.; Koutroubas, S.D. Farmers' Exposure to Pesticides: Toxicity Types and Ways of Prevention. Toxics 2016, 4, 1. https://doi.org/10.3390/toxics4010001.
5. Kim, K.H.; Kabir, E.; Jahan, S.A. Exposure to pesticides and the associated human health effects. Sci. Total Environ. 2017, 575, 525–535. https://doi.org/10.1016/j.scitotenv.2016.09.009.
6. Syafrudin, M.; Kristanti, R.A.; Yuniarto, A.; Hadibarata, T.; Rhee, J.; Al-Onazi, W.A.; Algarni, T.S.; Almarri, A.H.; Al-Mohaimeed, A.M. Pesticides in Drinking Water—A Review. Int. J. Environ. Res. Public Health 2021, 18, 468. https://doi.org/10.3390/ijerph18020468.
7. Jensen, B.H.; Petersen, A.; Petersen, P.B.; Christensen, T.; Fagt, S.; Trolle, E.; Poulsen, M.E.; Andersen, J.H. Cumulative dietary risk assessment of pesticides in food for the Danish population for the period 2012–2017. Food Chem. Toxicol. 2022, 168, 113359. https://doi.org/10.1016/j.fct.2022.113359.
8. Fucic, A.; Duca, R.C.; Galea, K.S.; Maric, T.; Garcia, K.; Bloom, M.S.; Andersen, H.R.; Vena, J.E. Reproductive Health Risks Associated with Occupational and Environmental Exposure to Pesticides. Int. J. Environ. Res. Public Health 2021, 18, 6576. https://doi.org/10.3390/ijerph18126576.
9. Richardson, J.R.; Fitsanakis, V.; Westerink, R.H.S.; Kanthasamy, A.G. Neurotoxicity of pesticides. Acta Neuropathol. 2019, 138, 343–362. https://doi.org/10.1007/s00401-019-02033-9.
10. Alavanja, M.C.; Ross, M.K.; Bonner, M.R. Increased cancer burden among pesticide applicators and others due to pesticide exposure. CA Cancer J. Clin. 2013, 63, 120–142. https://doi.org/10.3322/caac.21170.
11. Purdue, M.P.; Hoppin, J.A.; Blair, A.; Dosemeci, M.; Alavanja, M.C. Occupational exposure to organochlorine insecticides and cancer incidence in the Agricultural Health Study. Int. J. Cancer 2007, 120, 642–649. https://doi.org/10.1002/ijc.22258.
12. Mahajan, R.; Blair, A.; Lynch, C.F.; Schroeder, P.; Hoppin, J.A.; Sandler, D.P.; Alavanja, M.C. Fonofos exposure and cancer incidence in the agricultural health study. Environ. Health Perspect. 2006, 114, 1838–1842. https://doi.org/10.1289/ehp.9301.
13. Mokarizadeh, A.; Faryabi, M.R.; Rezvanfar, M.A.; Abdollahi, M. A comprehensive review of pesticides and the immune dysregulation: Mechanisms, evidence and consequences. Toxicol. Mech. Methods 2015, 25, 258–278. https://doi.org/10.3109/15376516.2015.1020182.
14. Costa, C.; Rapisarda, V.; Catania, S.; Di Nola, C.; Ledda, C.; Fenga, C. Cytokine patterns in greenhouse workers occupationally exposed to α-cypermethrin: An observational study. Environ. Toxicol. Pharmacol. 2013, 36, 796–800. https://doi.org/10.1016/j.etap.2013.07.004.
15. Takeuchi, T. Cytokines and cytokine receptors as targets of immune-mediated inflammatory diseases—RA as a role model. Inflamm. Regen. 2022, 42, 35. https://doi.org/10.1186/s41232-022-00221-x.
16. Gadina, M.; Le, M.T.; Schwartz, D.M.; Silvennoinen, O.; Nakayamada, S.; Yamaoka, K.; O'shea, J.J. Janus kinases to jakinibs: From basic insights to clinical practice. Rheumatology 2019, 58, i4–i16. https://doi.org/10.1093/rheumatology/key432.
17. Zarrin, A.A.; Bao, K.; Lupardus, P.; Vucic, D. Kinase inhibition in autoimmunity and inflammation. Nat. Rev. Drug Discov. 2021, 20, 39–63. https://doi.org/10.1038/s41573-020-0082-8.
18. Xue, C.; Yao, Q.; Gu, X.; Shi, Q.; Yuan, X.; Chu, Q.; Bao, Z.; Lu, J.; Li, L. Evolving cognition of the JAK-STAT signaling pathway: Autoimmune disorders and cancer. Signal Transduct. Target. Ther. 2023, 8, 204. https://doi.org/10.1038/s41392-023-01468-7.
19. Clark, J.D.; Flanagan, M.E.; Telliez, J.B. Discovery and development of Janus kinase (JAK) inhibitors for inflammatory diseases. J. Med. Chem. 2014, 57, 5023–5038. https://doi.org/10.1021/jm401490p.
20. Hoisnard, L.; Lebrun-Vignes, B.; Maury, S.; Mahevas, M.; El Karoui, K.; Roy, L.; Zarour, A.; Michel, M.; Cohen, J.L.; Amiot, A.; et al. Adverse events associated with JAK inhibitors in 126,815 reports from the WHO pharmacovigilance database. Sci. Rep. 2022, 12, 7140. https://doi.org/10.1038/s41598-022-10777-w.
21. Giordano, D.; Biancaniello, C.; Argenio, M.A.; Facchiano, A. Drug Design by Pharmacophore and Virtual Screening Approach. Pharmaceuticals 2022, 15, 646. https://doi.org/10.3390/ph15050646.

22. Wermuth, C.G.; Ganellin, C.R.; Lindberg, P.; Mitscher, L.A. Glossary of terms used in medicinal chemistry (IUPAC Recommendations 1998). Pure Appl. Chem. 1998, 70, 1129–1143. https://doi.org/10.1351/pac199870051129.
23. Kaserer, T.; Beck, K.R.; Akram, M.; Odermatt, A.; Schuster, D. Pharmacophore Models and Pharmacophore-Based Virtual Screening: Concepts and Applications Exemplified on Hydroxysteroid Dehydrogenases. Molecules 2015, 20, 22799–22832. https://doi.org/10.3390/molecules201219880.
24. Schuster, D.; Wolber, G. Identification of bioactive natural products by pharmacophore-based virtual screening. Curr. Pharm. Des. 2010, 16, 1666–1681. https://doi.org/10.2174/138161210791164072.
25. Simov, V.; Deshmukh, S.V.; Dinsmore, C.J.; Elwood, F.; Fernandez, R.B.; Garcia, Y.; Gibeau, C.; Gunaydin, H.; Jung, J.; Katz, J.D.; et al. Structure-based design and development of (benz)imidazole pyridones as JAK1-selective kinase inhibitors. Bioorganic Med. Chem. Lett. 2016, 26, 1803–1808. https://doi.org/10.1016/j.bmcl.2016.02.035.
26. Nakajima, Y.; Aoyama, N.; Takahashi, F.; Sasaki, H.; Hatanaka, K.; Moritomo, A.; Inami, M.; Ito, M.; Nakamura, K.; Nakamori, F.; et al. Design, synthesis, and evaluation of 4,6-diaminonicotinamide derivatives as novel and potent immunomodulators targeting JAK3. Bioorganic Med. Chem. 2016, 24, 4711–4722. https://doi.org/10.1016/j.bmc.2016.08.007.
27. Spergel, S.H.; Mertzman, M.E.; Kempson, J.; Guo, J.; Stachura, S.; Haque, L.; Lippy, J.S.; Zhang, R.F.; Galella, M.; Pitt, S.; et al. Discovery of a JAK1/3 Inhibitor and Use of a Prodrug To Demonstrate Efficacy in a Model of Rheumatoid Arthritis. ACS Med. Chem. Lett. 2019, 10, 306–311. https://doi.org/10.1021/acsmedchemlett.8b00508.
28. Raghuvanshi, R.; Bharate, S.B. Recent Developments in the Use of Kinase Inhibitors for Management of Viral Infections. J. Med. Chem. 2022, 65, 893–921. https://doi.org/10.1021/acs.jmedchem.0c01467.
29. Davis, R.R.; Li, B.; Yun, S.Y.; Chan, A.; Nareddy, P.; Gunawan, S.; Ayaz, M.; Lawrence, H.R.; Reuther, G.W.; Lawrence, N.J.; et al. Structural Insights into JAK2 Inhibition by Ruxolitinib, Fedratinib, and Derivatives Thereof. J. Med. Chem. 2021, 64, 2228–2241. https://doi.org/10.1021/acs.jmedchem.0c01952.
30. Mesaros, E.F.; Dugan, B.J.; Dorsey, B.D.; Milkiewicz, K.L.; Curry, M.A.; Gingrich, D.E. Preparation and Uses of 1,2,4-triazolo [1,5a] Pyridine Derivatives. U.S. Patent 8633173-B2, 5 June 2009.
31. Mathison, C.J.N.; Chianelli, D.; Rucker, P.V.; Nelson, J.; Roland, J.; Huang, Z.; Yang, Y.; Jiang, J.; Xie, Y.F.; Epple, R.; et al. Efficacy and Tolerability of Pyrazolo[1,5-a]pyrimidine RET Kinase Inhibitors for the Treatment of Lung Adenocarcinoma. ACS Med. Chem. Lett. 2020, 11, 558–565. https://doi.org/10.1021/acsmedchemlett.0c00015.
32. Yin, Y.; Chen, C.J.; Yu, R.N.; Shu, L.; Zhang, T.T.; Zhang, D.Y. Discovery of novel selective Janus kinase 2 (JAK2) inhibitors bearing a 1H-pyrazolo[3,4-d]pyrimidin-4-amino scaffold. Bioorganic Med. Chem. 2019, 27, 1562–1576. https://doi.org/10.1016/j.bmc.2019.02.054.
33. Sloman, D.L.; Noucti, N.; Altman, M.D.; Chen, D.; Mislak, A.C.; Szewczak, A.; Hayashi, M.; Warren, L.; Dellovade, T.; Wu, Z.; et al. Optimization of microtubule affinity regulating kinase (MARK) inhibitors with improved physical properties. Bioorganic Med. Chem. Lett. 2016, 26, 4362–4366. https://doi.org/10.1016/j.bmcl.2016.02.003.
34. Ma, X.; Diao, Y.; Ge, H.; Xu, F.; Zhu, L.; Zhao, Z.; Li, H. Discovery and optimization of 2-aminopyridine derivatives as novel and selective JAK2 inhibitors. Bioorganic Med. Chem. Lett. 2020, 30, 127048. https://doi.org/10.1016/j.bmcl.2020.127048.
35. Tan, L.; Akahane, K.; McNally, R.; Reyskens, K.M.S.E.; Ficarro, S.B.; Liu, S.; Herter-Sprie, G.S.; Koyama, S.; Pattison, M.J.; Labella, K.; et al. Development of Selective Covalent Janus Kinase 3 Inhibitors. J. Med. Chem. 2015, 58, 6589–6606. https://doi.org/10.1021/acs.jmedchem.5b00710.
36. Bhide, R.S.; Keon, A.; Weigelt, C.; Sack, J.S.; Schmidt, R.J.; Lin, S.; Xiao, H.-Y.; Spergel, S.H.; Kempson, J.; Pitts, W.J.; et al. Discovery and structure-based design of 4,6-diaminonicotinamides as potent and selective IRAK4 inhibitors. Bioorganic Med. Chem. Lett. 2017, 27, 4908–4913. https://doi.org/10.1016/j.bmcl.2017.09.029.
37. Fensome, A.; Ambler, C.M.; Arnold, E.; Banker, M.E.; Clark, J.D.; Dowty, M.E.; Efremov, I.V.; Flick, A.; Gerstenberger, B.S.; Gifford, R.S.; et al. Design and optimization of a series of 4-(3-azabicyclo[3.1.0]hexan-3-yl)pyrimidin-2-amines: Dual inhibitors of TYK2 and JAK1. Bioorganic Med. Chem. 2020, 28, 115481. https://doi.org/10.1016/j.bmc.2020.115481.
38. Lawrence, H.R.; Mahajan, K.; Luo, Y.; Zhang, D.; Tindall, N.; Huseyin, M.; Gevariya, H.; Kazi, S.; Ozcan, S.; Mahajan, N.P.; et al. Development of novel ACK1/TNK2 inhibitors using a fragment-based approach. J. Med. Chem. 2015, 58, 2746–2763. https://doi.org/10.1021/jm501929n.
39. Menet, C.J.; Fletcher, S.R.; Van Lommen, G.; Geney, R.; Blanc, J.; Smits, K.; Jouannigot, N.; Deprez, P.; van der Aar, E.M.; Clement-Lacroix, P.; et al. Triazolopyridines as selective JAK1 inhibitors: From hit identification to GLPG0634. J. Med. Chem. 2014, 57, 9323–9342. https://doi.org/10.1021/jm501262q.

40. B.S.M. (US) and P.A. (US). National Center for Biotechnology Information (2024). PubChem Patent Summary for US-9868729-B2, Inhibitors of Protein Kinases. United States. Available online: https://pubchem.ncbi.nlm.nih.gov/patent/US-9868729-B2 (accessed on 16 December 2024).
41. Krier, J.; Singh, R.R.; Kondić, T.; Lai, A.; Diderich, P.; Zhang, J.; Thiessen, P.A.; Bolton, E.E.; Schymanski, E.L. Discovering pesticides and their TPs in Luxembourg waters using open cheminformatics approaches. Environ. Int. 2022, 158, 106885. https://doi.org/10.1016/j.envint.2021.106885.
42. Wishart, D.S.; Guo, A.; Oler, E.; Wang, F.; Anjum, A.; Peters, H.; Dizon, R.; Sayeeda, Z.; Tian, S.; Lee, B.L.; et al. HMDB 5.0: The Human Metabolome Database for 2022. Nucleic Acids Res. 2022, 50, D622–D631. https://doi.org/10.1093/nar/gkab1062.
43. Ghosh, S.; Nie, A.; An, J.; Huang, Z. Structure-based virtual screening of chemical libraries for drug discovery. Curr. Opin. Chem. Biol. 2006, 10, 194–202. https://doi.org/10.1016/j.cbpa.2006.04.002.
44. Sun, H. Pharmacophore-based virtual screening. Curr. Med. Chem. 2008, 15, 1018–1024. https://doi.org/10.2174/092986708784049630.
45. Xin, P.; Xu, X.; Deng, C.; Liu, S.; Wang, Y.; Zhou, X.; Ma, H.; Wei, D.; Sun, S. The role of JAK/STAT signaling pathway and its inhibitors in diseases. Int. Immunopharmacol. 2020, 80, 106210. https://doi.org/10.1016/j.intimp.2020.106210.
46. Cohen, S.; Radominski, S.C.; Gomez - Reino, J.J.; Wang, L.; Krishnaswami, S.; Wood, S.P.; Soma, K.; Nduaka, C.I.; Kwok, K.; Valdez, H.; et al. Analysis of infections and all-cause mortality in phase II, phase III, and long-term extension studies of tofacitinib in patients with rheumatoid arthritis. Arthritis Rheumatol. 2014, 66, 2924–2937. https://doi.org/10.1002/art.38779.
47. Russell, M.D.; Stovin, C.; Alveyn, E.; Adeyemi, O.; Chan, C.K.D.; Patel, V.; Adas, M.A.; Atzeni, F.; Ng, K.K.H.; Rutherford, A.I.; et al. JAK inhibitors and the risk of malignancy: A meta-analysis across disease indications. Ann. Rheum. Dis. 2023, 82, 1059–1067. https://doi.org/10.1136/ard-2023-224049.
48. Zhang, J.; Li, W.; Gong, M.; Gu, Y.; Zhang, H.; Dong, B.; Guo, Q.; Pang, X.; Xiang, Q.; He, X.; et al. Risk of venous thromboembolism with janus kinase inhibitors in inflammatory immune diseases: A systematic review and meta-analysis. Front. Pharmacol. 2023, 14, 1189389. https://doi.org/10.3389/fphar.2023.1189389.
49. Robinson, C.; Portier, C.J.; Čavoški, A.; Mesnage, R.; Roger, A.; Clausing, P.; Whaley, P.; Muilerman, H.; Lyssimachou, A. Achieving a High Level of Protection from Pesticides in Europe: Problems with the Current Risk Assessment Procedure and Solutions. Eur. J. Risk Regul. 2020, 11, 450–480. https://doi.org/10.1017/err.2020.18.
50. Gerken, J.; Vincent, G.T.; Zapata, D.; Barron, I.G.; Zapata, I. Comprehensive assessment of pesticide use patterns and increased cancer risk. Front. Cancer Control Soc. 2024, 2, 1368086. https://doi.org/10.3389/fcacs.2024.1368086.
51. Yamazoe, Y.; Yamamoto, S.; Yoshida, M.; Kawanishi, T.; Kumagai, S. Mepanipyrim (Pesticides). Food Saf. 2016, 4, 28–29. https://doi.org/10.14252/foodsafetyfscj.2016001s.
52. Zweigle, J.; Schmidt, A.; Bugsel, B.; Vogel, C.; Simon, F.; Zwiener, C. Perfluoroalkyl acid precursor or weakly fluorinated organic compound? A proof of concept for oxidative fractionation of PFAS and organofluorines. Anal. Bioanal. Chem. 2024, 416, 6799–6808. https://doi.org/10.1007/s00216-024-05590-5.
53. Weis, G.C.C.; Assmann, C.E.; Cadoná, F.C.; Bonadiman, B.D.S.R.; de Oliveira Alves, A.; Machado, A.K.; Duarte, M.M.M.F.; da Cruz, I.B.M.; Costabeber, I.H. Immunomodulatory effect of mancozeb, chlorothalonil, and thiophanate methyl pesticides on macrophage cells. Ecotoxicol. Environ. Saf. 2019, 182, 109420. https://doi.org/10.1016/j.ecoenv.2019.109420.
54. Jabusch, T.W.; Tjeerdema, R.S. Partitioning of penoxsulam, a new sulfonamide herbicide. J. Agric. Food Chem. 2005, 53, 7179–7183. https://doi.org/10.1021/jf050767g.
55. Patel, D.M.; Gyldenkærne, S.; Jones, R.R.; Olsen, S.F.; Tikellis, G.; Granström, C.; Dwyer, T.; Stayner, L.T.; Ward, M.H. Residential proximity to agriculture and risk of childhood leukemia and central nervous system tumors in the Danish national birth cohort. Environ. Int. 2020, 143, 105955. https://doi.org/10.1016/j.envint.2020.105955.
56. Abarikwu, S.O.; Mgbudom-Okah, C.J.; Ndufeiya-Kumasi, L.C.; Monye, V.E.; Aruoren, O.; Ezim, O.E.; Omeodu, S.I.; Charles, I.A. Influence of triazines and lipopolysaccharide coexposure on inflammatory response and histopathological changes in the testis and liver of BalB/c mice. Heliyon 2024, 10, e24431. https://doi.org/10.1016/j.heliyon.2024.e24431.
57. Laetz, C.A.; Baldwin, D.H.; Collier, T.K.; Hebert, V.; Stark, J.D.; Scholz, N.L. The synergistic toxicity of pesticide mixtures: Implications for risk assessment and the conservation of endangered Pacific salmon. Environ. Health Perspect. 2009, 117, 348–353. https://doi.org/10.1289/ehp.0800096.
58. Berman, H.M.; Westbrook, J.; Feng, Z.; Gilliland, G.; Bhat, T.N.; Weissig, H.; Shindyalov, I.N.; Bourne, P.E. The Protein Data Bank. Nucleic Acids Res. 2000, 28, 235–242. https://doi.org/10.1093/nar/28.1.235.

59. Gaulton, A.; Bellis, L.J.; Bento, A.P.; Chambers, J.; Davies, M.; Hersey, A.; Light, Y.; McGlinchey, S.; Michalovich, D.; Al-Lazikani, B.; et al. ChEMBL: A large-scale bioactivity database for drug discovery. Nucleic Acids Res. 2012, 40, D1100–D1107. https://doi.org/10.1093/nar/gkr777.
60. Kim, S.; Chen, J.; Cheng, T.; Gindulyte, A.; He, J.; He, S.; Li, Q.; A Shoemaker, B.; A Thiessen, P.; Yu, B.; et al. PubChem 2023 update. Nucleic Acids Res. 2023, 51, D1373–D1380. https://doi.org/10.1093/nar/gkac956.
61. Mysinger, M.M.; Carchia, M.; Irwin, J.J.; Shoichet, B.K. Directory of useful decoys, enhanced (DUD-E): Better ligands and decoys for better benchmarking. J. Med. Chem. 2012, 55, 6582–6594. https://doi.org/10.1021/jm300687e.
62. Labadie, S.; Dragovich, P.S.; Barrett, K.; Blair, W.S.; Bergeron, P.; Chang, C.; Deshmukh, G.; Eigenbrot, C.; Ghilardi, N.; Gibbons, P.; et al. Structure-based discovery of C-2 substituted imidazo-pyrrolopyridine JAK1 inhibitors with improved selectivity over JAK2. Bioorganic Med. Chem. Lett. 2012, 22, 7627–7633. https://doi.org/10.1016/j.bmcl.2012.10.008.
63. Su, Q.; Banks, E.; Bebernitz, G.; Bell, K.; Borenstein, C.F.; Chen, H.; Chuaqui, C.E.; Deng, N.; Ferguson, A.D.; Kawatkar, S.P.; et al. Discovery of (2R)-N-[3-[2-[(3-Methoxy-1-methyl-pyrazol-4-yl)amino]pyrimidin-4-yl]-1H-indol-7-yl]-2-(4-methylpiperazin-1-yl)propenamide (AZD4205) as a Potent and Selective Janus Kinase 1 Inhibitor. J. Med. Chem. 2020, 63, 4517–4527. https://doi.org/10.1021/acs.jmedchem.9b01392.
64. Siu, T.; Brubaker, J.; Fuller, P.; Torres, L.; Zeng, H.; Close, J.; Mampreian, D.M.; Shi, F.; Liu, D.; Fradera, X.; et al. The Discovery of 3-((4-Chloro-3-methoxyphenyl)amino)-1-((3R,4S)-4-cyanotetrahydro-2H-pyran-3-yl)-1H-pyrazole-4-carboxamide, a Highly Ligand Efficient and Efficacious Janus Kinase 1 Selective Inhibitor with Favorable Pharmacokinetic Properties. J. Med. Chem. 2017, 60, 9676–9690. https://doi.org/10.1021/acs.jmedchem.7b01135.
65. Arwood, M.L.; Liu, Y.; Harkins, S.K.; Weinstock, D.M.; Yang, L.; Stevenson, K.E.; Plana, O.D.; Dong, J.; Cirka, H.; Jones, K.L.; et al. New scaffolds for type II JAK2 inhibitors overcome the acquired G993A resistance mutation. Cell Chem. Biol. 2023, 30, 618–631.e12. https://doi.org/10.1016/j.chembiol.2023.05.007.
66. Jaime-Figueroa, S.; De Vicente, J.; Hermann, J.; Jahangir, A.; Jin, S.; Kuglstatter, A.; Lynch, S.M.; Menke, J.; Niu, L.; Patel, V.; et al. Discovery of a series of novel 5H-pyrrolo[2,3-b]pyrazine-2-phenyl ethers, as potent JAK3 kinase inhibitors. Bioorganic Med. Chem. Lett. 2013, 23, 2522–2526. https://doi.org/10.1016/j.bmcl.2013.03.015.
67. Thorarensen, A.; Dowty, M.E.; Banker, M.E.; Juba, B.; Jussif, J.; Lin, T.; Vincent, F.; Czerwinski, R.M.; Casimiro-Garcia, A.; Unwalla, R.; et al. Design of a Janus Kinase 3 (JAK3) Specific Inhibitor 1-((2S,5R)-5-((7H-Pyrrolo[2,3-d]pyrimidin-4-yl)amino)-2-methylpiperidin-1-yl)prop-2-en-1-one (PF-06651600) Allowing for the Interrogation of JAK3 Signaling in Humans. J. Med. Chem. 2017, 60, 1971–1993. https://doi.org/10.1021/acs.jmedchem.6b01694.
68. Forster, M.; Chaikuad, A.; Bauer, S.M.; Holstein, J.; Robers, M.B.; Corona, C.R.; Gehringer, M.; Pfaffenrot, E.; Ghoreschi, K.; Knapp, S.; et al. Selective JAK3 Inhibitors with a Covalent Reversible Binding Mode Targeting a New Induced Fit Binding Pocket. Cell Chem. Biol. 2016, 23, 1335–1340. https://doi.org/10.1016/j.chembiol.2016.10.008.
69. Adams, C.; Aldous, D.J.; Amendola, S.; Bamborough, P.; Bright, C.; Crowe, S.; Eastwood, P.; Fenton, G.; Foster, M.; Harrison, T.K.P.; et al. Mapping the kinase domain of janus kinase 3. Bioorg. Med. Chem. Lett. 2003, 13, 3105–3110. https://doi.org/10.1016/S0960-894X(03)00657-7.
70. Burns, C.J.; Bourke, D.G.; Andrau, L.; Bu, X.; Charman, S.A.; Donohue, A.C.; Fantino, E.; Farrugia, M.; Feutrill, J.T.; Joffe, M.; et al. Phenylaminopyrimidines as inhibitors of Janus kinases (JAKs). Bioorg. Med. Chem. Lett. 2009, 19, 5887–5892. https://doi.org/10.1016/j.bmcl.2009.08.071.
71. Cole, A.G.; Bohnstedt, A.C.; Paradkar, V.; Kingsbury, C.; Quintero, J.G.; Park, H.; Lu, Y.; You, M.; Neagu, I.; Diller, D.J.; et al. 2-Benzimidazolyl-9-(chroman-4-yl)-purinone derivatives as JAK3 inhibitors. Bioorg. Med. Chem. Lett. 2009, 19, 6788–6792. https://doi.org/10.1016/j.bmcl.2009.09.080.
72. Gerspacher, M.; Furet, P.; Pissot-Soldermann, C.; Gaul, C.; Holzer, P.; Vangrevelinghe, E.; Lang, M.; Erdmann, D.; Radimerski, T.; Regnier, C.H.; et al. 2-Amino-aryl-7-aryl-benzoxazoles as potent, selective and orally available JAK2 inhibitors. Bioorg. Med. Chem. Lett. 2010, 20, 1724–1727. https://doi.org/10.1016/j.bmcl.2010.01.069.
73. Pissot-Soldermann, C.; Gerspacher, M.; Furet, P.; Gaul, C.; Holzer, P.; McCarthy, C.; Radimerski, T.; Regnier, C.H.; Baffert, F.; Drueckes, P.; et al. Discovery and SAR of potent, orally available 2,8-diaryl-quinoxalines as a new class of JAK2 inhibitors. Bioorg. Med. Chem. Lett. 2010, 20, 2609–2613. https://doi.org/10.1016/j.bmcl.2010.02.056.
74. Fidanze, S.D.; Erickson, S.A.; Wang, G.T.; Mantei, R.; Clark, R.F.; Sorensen, B.K.; Bamaung, N.Y.; Kovar, P.; Johnson, E.F.; Swinger, K.K.; et al. Imidazo[2,1-b]thiazoles: Multitargeted inhibitors of both the insulin-like growth factor receptor and members of the epidermal growth factor family of receptor tyrosine kinases. Bioorg. Med. Chem. Lett. 2010, 20, 2452–2455. https://doi.org/10.1016/j.bmcl.2010.03.015.

75. Dart, M.L.; Machleidt, T.; Jost, E.; Schwinn, M.K.; Robers, M.B.; Shi, C.; Kirkland, T.A.; Killoran, M.P.; Wilkinson, J.M.; Hartnett, J.R.; et al. Homogeneous Assay for Target Engagement Utilizing Bioluminescent Thermal Shift. ACS Med. Chem. Lett. 2018, 9, 546–551. https://doi.org/10.1021/acsmedchemlett.8b00081.
76. National Center for Biotechnology Information (2024). PubChem Bioassay Record for AID 1699, S.T.S.R.I.M.S.C.R.D., 2024 from https://pubchem.ncbi.nlm.nih.gov/bioassay/1699 .
77. Xu, P.; Shen, P.; Yu, B.; Xu, X.; Ge, R.; Cheng, X.; Chen, Q.; Bian, J.; Li, Z.; Wang, J. Janus kinases (JAKs): The efficient therapeutic targets for autoimmune diseases and myeloproliferative disorders. Eur. J. Med. Chem. 2020, 192, 112155. https://doi.org/10.1016/j.ejmech.2020.112155.
78. Belanger, D.B.; Williams, M.J.; Curran, P.J.; Mandal, A.K.; Meng, Z.; Rainka, M.P.; Yu, T.; Shih, N.Y.; Siddiqui, M.A.; Liu, M.; et al. Discovery of orally bioavailable imidazo[1,2-a]pyrazine-based Aurora kinase inhibitors. Bioorg. Med. Chem. Lett. 2010, 20, 6739–6743. https://doi.org/10.1016/j.bmcl.2010.08.140.
79. Siu, T.; Kozina, E.S.; Jung, J.; Rosenstein, C.; Mathur, A.; Altman, M.D.; Chan, G.; Xu, L.; Bachman, E.; Mo, J.R.; et al. The discovery of tricyclic pyridone JAK2 inhibitors. Part 1: Hit to lead. Bioorg. Med. Chem. Lett. 2010, 20, 7421–7425. https://doi.org/10.1016/j.bmcl.2010.10.031.
80. Wityak, J.; Das, J.; Moquin, R.V.; Shen, Z.; Lin, J.; Chen, P.; Doweyko, A.M.; Pitt, S.; Pang, S.; Shen, D.R.; et al. Discovery and initial SAR of 2-amino-5-carboxamidothiazoles as inhibitors of the Src-family kinase p56(Lck). Bioorg. Med. Chem. Lett. 2003, 13, 4007–4010. https://doi.org/10.1016/j.bmcl.2003.08.054.
81. Ma, H. (US); Filip, S.V. (US); Stent, M.A.H. (US); Dolan, J.A. (US); Dietrich, B. (US). National Center for Biotechnology Information (2024). Patent US-8592415-B2, JAK3 inhibitors for the treatment of autoimmune and inflammatory disorders. (grant date: 26 November 2013).
82. Ren, X.; Duan, L.; He, Q.; Zhang, Z.; Zhou, Y.; Wu, D.; Pan, J.; Pei, D.; Ding, K. Identification of Niclosamide as a New Small-Molecule Inhibitor of the STAT3 Signaling Pathway. ACS Med. Chem. Lett. 2010, 1, 454–459. https://doi.org/10.1021/ml100146z.
83. Flanagan, M.E.; Blumenkopf, T.A.; Brissette, W.H.; Brown, M.F.; Casavant, J.M.; Shang-Poa, C.; Doty, J.L.; Elliott, E.A.; Fisher, M.B.; Hines, M.; et al. Discovery of CP-690,550: A potent and selective Janus kinase (JAK) inhibitor for the treatment of autoimmune diseases and organ transplant rejection. J. Med. Chem. 2010, 53, 8468–8484. https://doi.org/10.1021/jm1004286.
84. Ioannidis, S.; Lamb, M.L.; Wang, T.; Almeida, L.; Block, M.H.; Davies, A.M.; Peng, B.; Su, M.; Zhang, H.J.; Hoffmann, E.; et al. Discovery of 5-chloro-N2-[(1S)-1-(5-fluoropyrimidin-2-yl)ethyl]-N4-(5-methyl-1H-pyrazol-3-yl)pyrimidine-2,4-diamine (AZD1480) as a novel inhibitor of the Jak/Stat pathway. J. Med. Chem. 2011, 54, 262–276. https://doi.org/10.1021/jm1011319.
85. McDonnell, M.E.; Bian, H.; Wrobel, J.; Smith, G.R.; Liang, S.; Ma, H.; Reitz, A.B. Anilino-monoindolylmaleimides as potent and selective JAK3 inhibitors. Bioorg. Med. Chem. Lett. 2014, 24, 1116–1121. https://doi.org/10.1016/j.bmcl.2014.01.001.
86. Harikrishnan, L.S.; Kamau, M.G.; Wan, H.; Inghrim, J.A.; Zimmermann, K.; Sang, X.; Mastalerz, H.A.; Johnson, W.L.; Zhang, G.; Lombardo, L.J.; et al. Pyrrolo[1,2-f]triazines as JAK2 inhibitors: Achieving potency and selectivity for JAK2 over JAK3. Bioorg. Med. Chem. Lett. 2011, 21, 1425–1428. https://doi.org/10.1016/j.bmcl.2011.01.022.
87. Kim, M.H.; Kim, M.; Yu, H.; Kim, H.; Yoo, K.H.; Sim, T.; Hah, J.M. Structure based design and syntheses of amino-1H-pyrazole amide derivatives as selective Raf kinase inhibitors in melanoma cells. Bioorg. Med. Chem. 2011, 19, 1915–1923. https://doi.org/10.1016/j.bmc.2011.01.067.
88. Medina, J.R.; Blackledge, C.W.; Heerding, D.A.; Campobasso, N.; Ward, P.; Briand, J.; Wright, L.; Axten, J.M. Aminoindazole PDK1 Inhibitors: A Case Study in Fragment-Based Drug Discovery. ACS Med. Chem. Lett. 2010, 1, 439–442. https://doi.org/10.1021/ml100136n.
89. Wang, T.; Ioannidis, S.; Almeida, L.; Block, M.H.; Davies, A.M.; Lamb, M.L.; Scott, D.A.; Su, M.; Zhang, H.J.; Alimzhanov, M.; et al. In vitro and in vivo evaluation of 6-aminopyrazolyl-pyridine-3-carbonitriles as JAK2 kinase inhibitors. Bioorg. Med. Chem. Lett. 2011, 21, 2958–2961. https://doi.org/10.1016/j.bmcl.2011.03.053.
90. Thomas, M.; Huang, W.S.; Wen, D.; Zhu, X.; Wang, Y.; Metcalf, C.A.; Liu, S.; Chen, I.; Romero, J.; Zou, D.; et al. Discovery of 5-(arenethynyl) hetero-monocyclic derivatives as potent inhibitors of BCR-ABL including the T315I gatekeeper mutant. Bioorg. Med. Chem. Lett. 2011, 21, 3743–3748. https://doi.org/10.1016/j.bmcl.2011.04.060.
91. Shu, L.; Chen, C.; Huan, X.; Huang, H.; Wang, M.; Zhang, J.; Yan, Y.; Liu, J.; Zhang, T.; Zhang, D. Design, synthesis, and pharmacological evaluation of 4- or 6-phenyl-pyrimidine derivatives as novel and selective Janus kinase 3 inhibitors. Eur. J. Med. Chem. 2020, 191, 112148. https://doi.org/10.1016/j.ejmech.2020.112148.
92. Qiu, Q.; Chi, F.; Zhou, D.; Xie, Z.; Liu, Y.; Wu, H.; Yin, Z.; Shi, W.; Qian, H. Exploration of Janus Kinase (JAK) and Histone Deacetylase (HDAC) Bispecific Inhibitors Based on the Moiety of Fedratinib for Treatment of Both Hematologic Malignancies and Solid Cancers. J. Med. Chem. 2023, 66, 5753–5773. https://doi.org/10.1021/acs.jmedchem.3c00036.

93. Lim, J.; Taoka, B.; Otte, R.D.; Spencer, K.; Dinsmore, C.J.; Altman, M.D.; Chan, G.; Rosenstein, C.; Sharma, S.; Su, H.P.; et al. Discovery of 1-amino-5H-pyrido[4,3-b]indol-4-carboxamide inhibitors of Janus kinase 2 (JAK2) for the treatment of myeloproliferative disorders. J. Med. Chem. 2011, 54, 7334–7349. https://doi.org/10.1021/jm200909u.
94. Giraud, F.; Marchand, P.; Carbonnelle, D.; Sartor, M.; Lang, F.; Duflos, M. Synthesis of N-aryl-3-(indol-3-yl)propanamides and their immunosuppressive activities. Bioorg. Med. Chem. Lett. 2010, 20, 5203–5206. https://doi.org/10.1016/j.bmcl.2010.07.001.
95. Gao, C.; Cahya, S.; Nicolaou, C.A.; Wang, J.; Watson, I.A.; Cummins, D.J.; Iversen, P.W.; Vieth, M. Selectivity data: Assessment, predictions, concordance, and implications. J. Med. Chem. 2013, 56, 6991–7002. https://doi.org/10.1021/jm400798j.
96. Liosi, M.E.; Krimmer, S.G.; Newton, A.S.; Dawson, T.K.; Puleo, D.E.; Cutrona, K.J.; Suzuki, Y.; Schlessinger, J.; Jorgensen, W.L. Selective Janus Kinase 2 (JAK2) Pseudokinase Ligands with a Diaminotriazole Core. J. Med. Chem. 2020, 63, 5324–5340. https://doi.org/10.1021/acs.jmedchem.0c00192.
97. Poulsen, A.; Blanchard, S.; Soh, C.K.; Lee, C.; Williams, M.; Wang, H.; Dymock, B. Structure-based design of PDK1 inhibitors. Bioorg. Med. Chem. Lett. 2012, 22, 305–307. https://doi.org/10.1016/j.bmcl.2011.11.006.
98. Zificsak, C.A.; Gingrich, D.E.; Breslin, H.J.; Dunn, D.D.; Milkiewicz, K.L.; Theroff, J.P.; Thieu, T.V.; Underiner, T.L.; Weinberg, L.R.; Aimone, L.D.; et al. Optimization of a novel kinase inhibitor scaffold for the dual inhibition of JAK2 and FAK kinases. Bioorg. Med. Chem. Lett. 2012, 22, 133–137. https://doi.org/10.1016/j.bmcl.2011.11.049.
99. Schenkel, L.B.; Huang, X.; Cheng, A.; Deak, H.L.; Doherty, E.; Emkey, R.; Gu, Y.; Gunaydin, H.; Kim, J.L.; Lee, J.; et al. Discovery of potent and highly selective thienopyridine Janus kinase 2 inhibitors. J. Med. Chem. 2011, 54, 8440–8450. https://doi.org/10.1021/jm200911r.
100. Sonawane, Y.A.; Taylor, M.A.; Napoleon, J.V.; Rana, S.; Contreras, J.I.; Natarajan, A. Cyclin Dependent Kinase 9 Inhibitors for Cancer Therapy. J. Med. Chem. 2016, 59, 8667–8684. https://doi.org/10.1021/acs.jmedchem.6b00150.
101. William, A.D.; Lee, A.C.; Goh, K.C.; Blanchard, S.; Poulsen, A.; Teo, E.L.; Nagaraj, H.; Lee, C.P.; Wang, H.; Williams, M.; et al. Discovery of kinase spectrum selective macrocycle (16E)-14-methyl-20-oxa-5,7,14,26-tetraazatetracyclo[19.3.1.1(2,6).1(8,12)]heptacosa-1(25),2(26),3,5,8(27),9,11,16,21,23-decaene (SB1317/TG02), a potent inhibitor of cyclin dependent kinases (CDKs), Janus kinase 2 (JAK2), and fms-like tyrosine kinase-3 (FLT3) for the treatment of cancer. J. Med. Chem. 2012, 55, 169–196. https://doi.org/10.1021/jm201112g.
102. Menichincheri, M.; Ardini, E.; Magnaghi, P.; Avanzi, N.; Banfi, P.; Bossi, R.; Buffa, L.; Canevari, G.; Ceriani, L.; Colombo, M.; et al. Discovery of Entrectinib: A New 3-Aminoindazole As a Potent Anaplastic Lymphoma Kinase (ALK), c-ros Oncogene 1 Kinase (ROS1), and Pan-Tropomyosin Receptor Kinases (Pan-TRKs) inhibitor. J. Med. Chem. 2016, 59, 3392–3408. https://doi.org/10.1021/acs.jmedchem.6b00064.
103. Liu, Z.; Wang, P.; Chen, H.; Wold, E.A.; Tian, B.; Brasier, A.R.; Zhou, J. Drug Discovery Targeting Bromodomain-Containing Protein 4. J. Med. Chem. 2017, 60, 4533–4558. https://doi.org/10.1021/acs.jmedchem.6b01761.
104. Hoemann, M.; Wilson, N.; Argiriadi, M.; Banach, D.; Burchat, A.; Calderwood, D.; Clapham, B.; Cox, P.; Duignan, D.B.; Konopacki, D.; et al. Synthesis and optimization of furano[3,2-d]pyrimidines as selective spleen tyrosine kinase (Syk) inhibitors. Bioorg. Med. Chem. Lett. 2016, 26, 5562–5567. https://doi.org/10.1016/j.bmcl.2016.09.077.
105. Oza, V.; Ashwell, S.; Brassil, P.; Breed, J.; Ezhuthachan, J.; Deng, C.; Grondine, M.; Horn, C.; Liu, D.; Lyne, P.; et al. Synthesis and evaluation of triazolones as checkpoint kinase 1 inhibitors. Bioorg. Med. Chem. Lett. 2012, 22, 2330–2337. https://doi.org/10.1016/j.bmcl.2012.01.043.
106. Yang, E.G.; Mustafa, N.; Tan, E.C.; Poulsen, A.; Ramanujulu, P.M.; Chng, W.J.; Yen, J.J.; Dymock, B.W. Design and Synthesis of Janus Kinase 2 (JAK2) and Histone Deacetlyase (HDAC) Bispecific Inhibitors Based on Pacritinib and Evidence of Dual Pathway Inhibition in Hematological Cell Lines. J. Med. Chem. 2016, 59, 8233–8262. https://doi.org/10.1021/acs.jmedchem.6b00157.
107. Dugan, B.J.; Gingrich, D.E.; Mesaros, E.F.; Milkiewicz, K.L.; Curry, M.A.; Zulli, A.L.; Dobrzanski, P.; Serdikoff, C.; Jan, M.; Angeles, T.S.; et al. A selective, orally bioavailable 1,2,4-triazolo[1,5-a]pyridine-based inhibitor of Janus kinase 2 for use in anticancer therapy: Discovery of CEP-33779. J. Med. Chem. 2012, 55, 5243–5254. https://doi.org/10.1021/jm300248q.
108. Mitton-Fry, M.J. (US); Berlinski, P.J. (US); Birchmeier, M.J. (US); Bowman, J.W. (US); Gonzales, A.J. (US); et al. National Center for Biotechnology Information (2024). Patent US-9161939-B2, Pyrrolo[2,3-d]pyrimidine compounds. (grant date: 20 October 2015)
109. Yogo, T.; Nagamiya, H.; Seto, M.; Sasaki, S.; Shih-Chung, H.; Ohba, Y.; Tokunaga, N.; Lee, G.N.; Rhim, C.Y.; Yoon, C.H.; et al. Structure-Based Design and Synthesis of 3-Amino-1,5-dihydro-4H-pyrazolopyridin-4-one Derivatives as Tyrosine Kinase 2 Inhibitors. J. Med. Chem. 2016, 59, 733–749. https://doi.org/10.1021/acs.jmedchem.5b01857.
110. Kim, M.K.; Shin, H.; Park, K.S.; Kim, H.; Park, J.; Kim, K.; Nam, J.; Choo, H.; Chong, Y. Benzimidazole Derivatives as Potent JAK1-Selective Inhibitors. J. Med. Chem. 2015, 58, 7596–7602. https://doi.org/10.1021/acs.jmedchem.5b01263.

111. Chen, X.; Wilson, L.J.; Malaviya, R.; Argentieri, R.L.; Yang, S.M. Virtual screening to successfully identify novel janus kinase 3 inhibitors: A sequential focused screening approach. J. Med. Chem. 2008, 51, 7015–7019. https://doi.org/10.1021/jm800662z.
112. Fedorov, O.; Marsden, B.; Pogacic, V.; Rellos, P.; Müller, S.; Bullock, A.N.; Schwaller, J.; Sundström, M.; Knapp, S. A systematic interaction map of validated kinase inhibitors with Ser/Thr kinases. Proc. Natl. Acad. Sci. USA 2007, 104, 20523–20528. https://doi.org/10.1073/pnas.0708800104.
113. DiMauro, E.F.; Newcomb, J.; Nunes, J.J.; Bemis, J.E.; Boucher, C.; Buchanan, J.L.; Buckner, W.H.; Cee, V.J.; Chai, L.; Deak, H.L.; et al. Discovery of aminoquinazolines as potent, orally bioavailable inhibitors of Lck: Synthesis, SAR, and in vivo anti-inflammatory activity. J. Med. Chem. 2006, 49, 5671–5686. https://doi.org/10.1021/jm0605482.
114. Wang, T.; Lamb, M.L.; Block, M.H.; Davies, A.M.; Han, Y.; Hoffmann, E.; Ioannidis, S.; Josey, J.A.; Liu, Z.Y.; Lyne, P.D.; et al. Discovery of Disubstituted Imidazo[4,5-b]pyridines and Purines as Potent TrkA Inhibitors. ACS Med. Chem. Lett. 2012, 3, 705–709. https://doi.org/10.1021/ml300074j.
115. Liu, M.; Ju, X.; Zou, J.; Shi, J.; Jia, G. Recent researches for dual Aurora target inhibitors in antitumor field. Eur. J. Med. Chem. 2020, 203, 112498. https://doi.org/10.1016/j.ejmech.2020.112498.
116. Lawrence, H.R.; Martin, M.P.; Luo, Y.; Pireddu, R.; Yang, H.; Gevariya, H.; Ozcan, S.; Zhu, J.Y.; Kendig, R.; Rodriguez, M.; et al. Development of o-chlorophenyl substituted pyrimidines as exceptionally potent aurora kinase inhibitors. J. Med. Chem. 2012, 55, 7392–7416. https://doi.org/10.1021/jm300334d.
117. Hanan, E.J.; van Abbema, A.; Barrett, K.; Blair, W.S.; Blaney, J.; Chang, C.; Eigenbrot, C.; Flynn, S.; Gibbons, P.; Hurley, C.A.; et al. Discovery of potent and selective pyrazolopyrimidine janus kinase 2 inhibitors. J. Med. Chem. 2012, 55, 10090–10107. https://doi.org/10.1021/jm3012239.
118. William, A.D.; Lee, A.C.; Blanchard, S.; Poulsen, A.; Teo, E.L.; Nagaraj, H.; Tan, E.; Chen, D.; Williams, M.; Sun, E.T.; et al. Discovery of the macrocycle 11-(2-pyrrolidin-1-yl-ethoxy)-14,19-dioxa-5,7,26-triaza-tetracyclo[19.3.1.1(2,6).1(8,12)]heptacosa-1(25),2(26),3,5,8,10,12(27),16,21,23-decaene (SB1518), a potent Janus kinase 2/fms-like tyrosine kinase-3 (JAK2/FLT3) inhibitor for the treatment of myelofibrosis and lymphoma. J. Med. Chem. 2011, 54, 4638–4658. https://doi.org/10.1021/jm200326p.
119. Forsyth, T.; Kearney, P.C.; Kim, B.G.; Johnson, H.W.; Aay, N.; Arcalas, A.; Brown, D.S.; Chan, V.; Chen, J.; Du, H.; et al. SAR and in vivo evaluation of 4-aryl-2-aminoalkylpyrimidines as potent and selective Janus kinase 2 (JAK2) inhibitors. Bioorg. Med. Chem. Lett. 2012, 22, 7653–7658. https://doi.org/10.1016/j.bmcl.2012.10.007.
120. Chen, J.J.; Thakur, K.D.; Clark, M.P.; Laughlin, S.K.; George, K.M.; Bookland, R.G.; Davis, J.R.; Cabrera, E.J.; Easwaran, V.; De, B.; et al. Development of pyrimidine-based inhibitors of Janus tyrosine kinase 3. Bioorg. Med. Chem. Lett. 2006, 16, 5633–5638. https://doi.org/10.1016/j.bmcl.2006.08.022.
121. Liang, X.; Huang, Y.; Zang, J.; Gao, Q.; Wang, B.; Xu, W.; Zhang, Y. Design, synthesis and preliminary biological evaluation of 4-aminopyrazole derivatives as novel and potent JAKs inhibitors. Bioorg. Med. Chem. 2016, 24, 2660–2672. https://doi.org/10.1016/j.bmc.2016.04.030.
122. Yu, R.N.; Chen, C.J.; Shu, L.; Yin, Y.; Wang, Z.J.; Zhang, T.T.; Zhang, D.Y. Structure-based design and synthesis of pyrimidine-4,6-diamine derivatives as Janus kinase 3 inhibitors. Bioorg. Med. Chem. 2019, 27, 1646–1657. https://doi.org/10.1016/j.bmc.2019.03.009.
123. Elsayed, M.S.A.; Nielsen, J.J.; Park, S.; Park, J.; Liu, Q.; Kim, C.H.; Pommier, Y.; Agama, K.; Low, P.S.; Cushman, M. Application of Sequential Palladium Catalysis for the Discovery of Janus Kinase Inhibitors in the Benzo[1]pyrrolo[2,3- h][1,6]naphthyridin-5-one (BPN) Series. J. Med. Chem. 2018, 61, 10440–10462. https://doi.org/10.1021/acs.jmedchem.8b00510.
124. Uckun, F.M.; Dibirdik, I.; Qazi, S.; Vassilev, A.; Ma, H.; Mao, C.; Benyumov, A.; Emami, K.H. Anti-breast cancer activity of LFM-A13, a potent inhibitor of Polo-like kinase (PLK). Bioorg. Med. Chem. 2007, 15, 800–814. https://doi.org/10.1016/j.bmc.2006.10.050.
125. Van Epps, S.; Fiamengo, B.; Edmunds, J.; Ericsson, A.; Frank, K.; Friedman, M.; George, D.; George, J.; Goedken, E.; Kotecki, B.; et al. Design and synthesis of tricyclic cores for kinase inhibition. Bioorg. Med. Chem. Lett. 2013, 23, 693–698. https://doi.org/10.1016/j.bmcl.2012.11.108.
126. Soth, M.; Hermann, J.C.; Yee, C.; Alam, M.; Barnett, J.W.; Berry, P.; Browner, M.F.; Frank, K.; Frauchiger, S.; Harris, S.; et al. 3-Amido pyrrolopyrazine JAK kinase inhibitors: Development of a JAK3 vs JAK1 selective inhibitor and evaluation in cellular and in vivo models. J. Med. Chem. 2013, 56, 345–356. https://doi.org/10.1021/jm301646k.
127. Yang, S.M.; Malaviya, R.; Wilson, L.J.; Argentieri, R.; Chen, X.; Yang, C.; Wang, B.; Cavender, D.; Murray, W.V. Simplified staurosporine analogs as potent JAK3 inhibitors. Bioorg. Med. Chem. Lett. 2007, 17, 326–331. https://doi.org/10.1016/j.bmcl.2006.10.062.

128. Lynch, S.M.; DeVicente, J.; Hermann, J.C.; Jaime-Figueroa, S.; Jin, S.; Kuglstatter, A.; Li, H.; Lovey, A.; Menke, J.; Niu, L.; et al. Strategic use of conformational bias and structure based design to identify potent JAK3 inhibitors with improved selectivity against the JAK family and the kinome. Bioorg. Med. Chem. Lett. 2013, 23, 2793–2800. https://doi.org/10.1016/j.bmcl.2013.02.012.
129. Guan, H.; Lamb, M.L.; Peng, B.; Huang, S.; Degrace, N.; Read, J.; Hussain, S.; Wu, J.; Rivard, C.; Alimzhanov, M.; et al. Discovery of novel Jak2-Stat pathway inhibitors with extended residence time on target. Bioorg. Med. Chem. Lett. 2013, 23, 3105–3110. https://doi.org/10.1016/j.bmcl.2013.02.111.
130. Marsilje, T.H.; Pei, W.; Chen, B.; Lu, W.; Uno, T.; Jin, Y.; Jiang, T.; Kim, S.; Li, N.; Warmuth, M.; et al. Synthesis, structure-activity relationships, and in vivo efficacy of the novel potent and selective anaplastic lymphoma kinase (ALK) inhibitor 5-chloro-N2-(2-isopropoxy-5-methyl-4-(piperidin-4-yl)phenyl)-N4-(2-(isopropylsulfonyl)phenyl)pyrimidine-2,4-diamine (LDK378) currently in phase 1 and phase 2 clinical trials. J. Med. Chem. 2013, 56, 5675–5690. https://doi.org/10.1021/jm400402q.
131. Martin, M.W.; Newcomb, J.; Nunes, J.J.; Bemis, J.E.; McGowan, D.C.; White, R.D.; Buchanan, J.L.; DiMauro, E.F.; Boucher, C.; Faust, T.; et al. Discovery of novel 2,3-diarylfuro[2,3-b]pyridin-4-amines as potent and selective inhibitors of Lck: Synthesis, SAR, and pharmacokinetic properties. Bioorg. Med. Chem. Lett. 2007, 17, 2299–2304. https://doi.org/10.1016/j.bmcl.2007.01.048.
132. Siu, M.; Pastor, R.; Liu, W.; Barrett, K.; Berry, M.; Blair, W.S.; Chang, C.; Chen, J.Z.; Eigenbrot, C.; Ghilardi, N.; et al. 2-Amino-[1,2,4]triazolo[1,5-a]pyridines as JAK2 inhibitors. Bioorg. Med. Chem. Lett. 2013, 23, 5014–5021. https://doi.org/10.1016/j.bmcl.2013.06.008.
133. Brasca, M.G.; Mantegani, S.; Amboldi, N.; Bindi, S.; Caronni, D.; Casale, E.; Ceccarelli, W.; Colombo, N.; De Ponti, A.; Donati, D.; et al. Discovery of NMS-E973 as novel, selective and potent inhibitor of heat shock protein 90 (Hsp90). Bioorg. Med. Chem. 2013, 21, 7047–7063. https://doi.org/10.1016/j.bmc.2013.09.018.
134. DiMauro, E.F.; Newcomb, J.; Nunes, J.J.; Bemis, J.E.; Boucher, C.; Buchanan, J.L.; Buckner, W.H.; Cheng, A.; Faust, T.; Hsieh, F.; et al. Discovery of 4-amino-5,6-biaryl-furo[2,3-d]pyrimidines as inhibitors of Lck: Development of an expedient and divergent synthetic route and preliminary SAR. Bioorg. Med. Chem. Lett. 2007, 17, 2305–2309. https://doi.org/10.1016/j.bmcl.2007.01.057.
135. Côté, B.; Boulet, L.; Brideau, C.; Claveau, D.; Ethier, D.; Frenette, R.; Gagnon, M.; Giroux, A.; Guay, J.; Guiral, S.; et al. Substituted phenanthrene imidazoles as potent, selective, and orally active mPGES-1 inhibitors. Bioorg. Med. Chem. Lett. 2007, 17, 6816–6820. https://doi.org/10.1016/j.bmcl.2007.10.033.
136. Seerden, J.P.; Leusink-Ionescu, G.; Woudenberg-Vrenken, T.; Dros, B.; Molema, G.; Kamps, J.A.; Kellogg, R.M. Synthesis and structure-activity relationships of 4-fluorophenyl-imidazole p38α MAPK, CK1δ and JAK2 kinase inhibitors. Bioorg. Med. Chem. Lett. 2014, 24, 3412–3418. https://doi.org/10.1016/j.bmcl.2014.05.080.
137. Zhong, Y.; Qiu, R.Z.; Sun, S.L.; Zhao, C.; Fan, T.Y.; Chen, M.; Li, N.G.; Shi, Z.H. Small-Molecule Fms-like Tyrosine Kinase 3 Inhibitors: An Attractive and Efficient Method for the Treatment of Acute Myeloid Leukemia. J. Med. Chem. 2020, 63, 12403–12428. https://doi.org/10.1021/acs.jmedchem.0c00696.
138. Zhao, H.; Caflisch, A. Discovery of ZAP70 inhibitors by high-throughput docking into a conformation of its kinase domain generated by molecular dynamics. Bioorg. Med. Chem. Lett. 2013, 23, 5721–5726. https://doi.org/10.1016/j.bmcl.2013.08.009.
139. Su, Q.; Ioannidis, S.; Chuaqui, C.; Almeida, L.; Alimzhanov, M.; Bebernitz, G.; Bell, K.; Block, M.; Howard, T.; Huang, S.; et al. Discovery of 1-methyl-1H-imidazole derivatives as potent Jak2 inhibitors. J. Med. Chem. 2014, 57, 144–158. https://doi.org/10.1021/jm401546n.
140. Siu, T.; Kumarasinghe, S.E.; Altman, M.D.; Katcher, M.; Northrup, A.; White, C.; Rosenstein, C.; Mathur, A.; Xu, L.; Chan, G.; et al. The discovery of reverse tricyclic pyridone JAK2 inhibitors. Part 2: Lead optimization. Bioorg. Med. Chem. Lett. 2014, 24, 1466–1471. https://doi.org/10.1016/j.bmcl.2014.02.011.
141. Huang, Q.; Johnson, T.W.; Bailey, S.; Brooun, A.; Bunker, K.D.; Burke, B.J.; Collins, M.R.; Cook, A.S.; Cui, J.J.; Dack, K.N.; et al. Design of potent and selective inhibitors to overcome clinical anaplastic lymphoma kinase mutations resistant to crizotinib. J. Med. Chem. 2014, 57, 1170–1187. https://doi.org/10.1021/jm401805h.
142. Shen, P.; Wang, Y.; Jia, X.; Xu, P.; Qin, L.; Feng, X.; Li, Z.; Qiu, Z. Dual-target Janus kinase (JAK) inhibitors: Comprehensive review on the JAK-based strategies for treating solid or hematological malignancies and immune-related diseases. Eur. J. Med. Chem. 2022, 239, 114551. https://doi.org/10.1016/j.ejmech.2022.114551.
143. Wang, S.; Zhang, R.H.; Zhang, H.; Wang, Y.C.; Yang, D.; Zhao, Y.L.; Yan, G.Y.; Xu, G.B.; Guan, H.Y.; Zhou, Y.H.; et al. Design, synthesis, and biological evaluation of 2,4-diamino pyrimidine derivatives as potent FAK inhibitors with anti-cancer and anti-angiogenesis activities. Eur. J. Med. Chem. 2021, 222, 113573. https://doi.org/10.1016/j.ejmech.2021.113573.
144. Haidle, A.M.; Zabierek, A.A.; Childers, K.K.; Rosenstein, C.; Mathur, A.; Altman, M.D.; Chan, G.; Xu, L.; Bachman, E.; Mo, J.R.; et al. Thiophene carboxamide inhibitors of JAK2 as potential treatments for myleoproliferative neoplasms. Bioorg. Med. Chem. Lett. 2014, 24, 1968–1973. https://doi.org/10.1016/j.bmcl.2014.02.064.

145. Costales, A.; Mathur, M.; Ramurthy, S.; Lan, J.; Subramanian, S.; Jain, R.; Atallah, G.; Setti, L.; Lindvall, M.; Appleton, B.A.; et al. 2-Amino-7-substituted benzoxazole analogs as potent RSK2 inhibitors. Bioorg. Med. Chem. Lett. 2014, 24, 1592–1596. https://doi.org/10.1016/j.bmcl.2014.01.058.
146. Park, E.; Lee, S.J.; Moon, H.; Park, J.; Jeon, H.; Hwang, J.S.; Hwang, H.; Hong, K.B.; Han, S.H.; Choi, S.; et al. Discovery and Biological Evaluation of. J. Med. Chem. 2021, 64, 958–979. https://doi.org/10.1021/acs.jmedchem.0c01026.
147. Ren, J.; Shi, W.; Zhao, D.; Wang, Q.; Chang, X.; He, X.; Wang, X.; Gao, Y.; Lu, P.; Zhang, X.; et al. Design and synthesis of boron-containing diphenylpyrimidines as potent BTK and JAK3 dual inhibitors. Bioorg. Med. Chem. 2020, 28, 115236. https://doi.org/10.1016/j.bmc.2019.115236.
148. Brasca, M.G.; Nesi, M.; Avanzi, N.; Ballinari, D.; Bandiera, T.; Bertrand, J.; Bindi, S.; Canevari, G.; Carenzi, D.; Casero, D.; et al. Pyrrole-3-carboxamides as potent and selective JAK2 inhibitors. Bioorg. Med. Chem. 2014, 22, 4998–5012. https://doi.org/10.1016/j.bmc.2014.06.025.
149. Hanan, E.J.; Eigenbrot, C.; Bryan, M.C.; Burdick, D.J.; Chan, B.K.; Chen, Y.; Dotson, J.; Heald, R.A.; Jackson, P.S.; La, H.; et al. Discovery of selective and noncovalent diaminopyrimidine-based inhibitors of epidermal growth factor receptor containing the T790M resistance mutation. J. Med. Chem. 2014, 57, 10176–10191. https://doi.org/10.1021/jm501578n.
150. Goodwin, N.C.; Cianchetta, G.; Burgoon, H.A.; Healy, J.; Mabon, R.; Strobel, E.D.; Allen, J.; Wang, S.; Hamman, B.D.; Rawlins, D.B. Discovery of a Type III Inhibitor of LIM Kinase 2 That Binds in a DFG-Out Conformation. ACS Med. Chem. Lett. 2015, 6, 53–57. https://doi.org/10.1021/ml500242y.
151. Duan, J.J.; Lu, Z.; Jiang, B.; Yang, B.V.; Doweyko, L.M.; Nirschl, D.S.; Haque, L.E.; Lin, S.; Brown, G.; Hynes, J.; et al. Discovery of pyrrolo[1,2-b]pyridazine-3-carboxamides as Janus kinase (JAK) inhibitors. Bioorg. Med. Chem. Lett. 2014, 24, 5721–5726. https://doi.org/10.1016/j.bmcl.2014.10.061.
152. Henderson, J.L.; Kormos, B.L.; Hayward, M.M.; Coffman, K.J.; Jasti, J.; Kurumbail, R.G.; Wager, T.T.; Verhoest, P.R.; Noell, G.S.; Chen, Y.; et al. Discovery and preclinical profiling of 3-[4-(morpholin-4-yl)-7H-pyrrolo[2,3-d]pyrimidin-5-yl]benzonitrile (PF-06447475), a highly potent, selective, brain penetrant, and in vivo active LRRK2 kinase inhibitor. J. Med. Chem. 2015, 58, 419–432. https://doi.org/10.1021/jm5014055.
153. Brasca, M.G.; Gnocchi, P.; Nesi, M.; Amboldi, N.; Avanzi, N.; Bertrand, J.; Bindi, S.; Canevari, G.; Casero, D.; Ciomei, M.; et al. Novel pyrrole carboxamide inhibitors of JAK2 as potential treatment of myeloproliferative disorders. Bioorg. Med. Chem. 2015, 23, 2387–2407. https://doi.org/10.1016/j.bmc.2015.03.059.
154. Wan, H.; Schroeder, G.M.; Hart, A.C.; Inghrim, J.; Grebinski, J.; Tokarski, J.S.; Lorenzi, M.V.; You, D.; Mcdevitt, T.; Penhallow, B.; et al. Discovery of a Highly Selective JAK2 Inhibitor, BMS-911543, for the Treatment of Myeloproliferative Neoplasms. ACS Med. Chem. Lett. 2015, 6, 850–855. https://doi.org/10.1021/acsmedchemlett.5b00226.
155. Zimmermann, K.; Sang, X.; Mastalerz, H.A.; Johnson, W.L.; Zhang, G.; Liu, Q.; Batt, D.; Lombardo, L.J.; Vyas, D.; Trainor, G.L.; et al. 9H-Carbazole-1-carboxamides as potent and selective JAK2 inhibitors. Bioorg. Med. Chem. Lett. 2015, 25, 2809–2812. https://doi.org/10.1016/j.bmcl.2015.04.101.
156. Yamagishi, H.; Shirakami, S.; Nakajima, Y.; Tanaka, A.; Takahashi, F.; Hamaguchi, H.; Hatanaka, K.; Moritomo, A.; Inami, M.; Higashi, Y.; et al. Discovery of 3,6-dihydroimidazo[4,5-d]pyrrolo[2,3-b]pyridin-2(1H)-one derivatives as novel JAK inhibitors. Bioorg. Med. Chem. 2015, 23, 4846–4859. https://doi.org/10.1016/j.bmc.2015.05.028.
157. Nakajima, Y.; Inoue, T.; Nakai, K.; Mukoyoshi, K.; Hamaguchi, H.; Hatanaka, K.; Sasaki, H.; Tanaka, A.; Takahashi, F.; Kunikawa, S.; et al. Synthesis and evaluation of novel 1H-pyrrolo[2,3-b]pyridine-5-carboxamide derivatives as potent and orally efficacious immunomodulators targeting JAK3. Bioorg. Med. Chem. 2015, 23, 4871–4883. https://doi.org/10.1016/j.bmc.2015.05.034.
158. Jang, W.D.; Kim, J.T.; Son, H.Y.; Park, S.Y.; Cho, Y.S.; Koo, T.S.; Lee, H.; Kang, N.S. Discovery of Tyk2 inhibitors via the virtual site-directed fragment-based drug design. Bioorg. Med. Chem. Lett. 2015, 25, 3947–3952. https://doi.org/10.1016/j.bmcl.2015.07.037.
159. Liu, Q.; Batt, D.G.; Lippy, J.S.; Surti, N.; Tebben, A.J.; Muckelbauer, J.K.; Chen, L.; An, Y.; Chang, C.; Pokross, M.; et al. Design and synthesis of carbazole carboxamides as promising inhibitors of Bruton's tyrosine kinase (BTK) and Janus kinase 2 (JAK2). Bioorg. Med. Chem. Lett. 2015, 25, 4265–4269. https://doi.org/10.1016/j.bmcl.2015.07.102.
160. Hart, A.C.; Schroeder, G.M.; Wan, H.; Grebinski, J.; Inghrim, J.; Kempson, J.; Guo, J.; Pitts, W.J.; Tokarski, J.S.; Sack, J.S.; et al. Structure-Based Design of Selective Janus Kinase 2 Imidazo[4,5-d]pyrrolo[2,3-b]pyridine Inhibitors. ACS Med. Chem. Lett. 2015, 6, 845–849. https://doi.org/10.1021/acsmedchemlett.5b00225.
161. Gehringer, M.; Laufer, S.A. Emerging and Re-Emerging Warheads for Targeted Covalent Inhibitors: Applications in Medicinal Chemistry and Chemical Biology. J. Med. Chem. 2019, 62, 5673–5724. https://doi.org/10.1021/acs.jmedchem.8b01153.

162. ChEMBL Database, EMBL-EBI (2024). ChEMBL Document: Affinity Phenotypic Cellular Literature for EUbOPEN Chemogenomic Library (CHEMBL5444388) - ChEMBL(20 January 2022)
163. Choi, J.S.; Hwang, H.J.; Kim, S.W.; Lee, B.I.; Lee, J.; Song, H.J.; Koh, J.S.; Kim, J.H.; Lee, P.H. Highly potent and selective pyrazolylpyrimidines as Syk kinase inhibitors. Bioorg. Med. Chem. Lett. 2015, 25, 4441–4446. https://doi.org/10.1016/j.bmcl.2015.09.011.
164. Curry, M.A. (US); Dorsey, B.D. (US); Dugan, B.J. (US); Gingrich, D.E. (US); Mesaros, E.F. (US); et al. National Center for Biotechnology Information (2024).Patent US-8633173-B2, Preparation and uses of 1,2,4-triazolo[1,5a]pyridine derivatives. (grant date: 21 January 2014).
165. Jones, P.; Storer, R.I.; Sabnis, Y.A.; Wakenhut, F.M.; Whitlock, G.A.; England, K.S.; Mukaiyama, T.; Dehnhardt, C.M.; Coe, J.W.; Kortum, S.W.; et al. Design and Synthesis of a Pan-Janus Kinase Inhibitor Clinical Candidate (PF-06263276) Suitable for Inhaled and Topical Delivery for the Treatment of Inflammatory Diseases of the Lungs and Skin. J. Med. Chem. 2017, 60, 767–786. https://doi.org/10.1021/acs.jmedchem.6b01634.
166. Casimiro-Garcia, A.; Trujillo, J.I.; Vajdos, F.; Juba, B.; Banker, M.E.; Aulabaugh, A.; Balbo, P.; Bauman, J.; Chrencik, J.; Coe, J.W.; et al. Identification of Cyanamide-Based Janus Kinase 3 (JAK3) Covalent Inhibitors. J. Med. Chem. 2018, 61, 10665–10699. https://doi.org/10.1021/acs.jmedchem.8b01308.
167. Bauer, S.M. (US); Jia, Z.J. (US); Mehrotra, M. (US); Song, Y. (US); Xu, Q. (US); et al. National Center for Biotechnology Information (2024). Patent US-8501944-B2, Inhibitors of syk and/or JAK kinase. (grant date: 6 August 2013).
168. Purandare, A.V. (US); Batt, D.G. (US); Liu, Q. (US); Mastalerz, H. (US); Zimmermann, K. (US); et al. National Center for Biotechnology Information (2024). Patent US-8815840-B2, Carbazole and carboline kinase inhibitors. United States. (grant date: 26 August 2014).
169. Woo, H.C. (US); Cash, B. (US); Ahearn, S.P. (US); Dinsmore, C. (US); Jung, J. (US); Pu, Q. (US); Rivkin, A. (US); Scott, M.E. (US); Witter, D.J. (US); et al. National Center for Biotechnology Information (2024).Patent US-8993756-B2, Pyrrolopyrimidines as Janus kinase inhibitors. (grant date: 31 March 2015).
170. Forster, M.; Gehringer, M.; Laufer, S.A. Recent advances in JAK3 inhibition: Isoform selectivity by covalent cysteine targeting. Bioorg. Med. Chem. Lett. 2017, 27, 4229–4237. https://doi.org/10.1016/j.bmcl.2017.07.079.
171. M.A. (US); Xie, J. (US); Acker, B.A. (US); Hartmann, S.J. (US); Wolfson, S.G. (US); Huang, H.-C. (US); Jacobsen, E.J. (US). National Center for Biotechnology Information (2024). Patent US-8633206-B2, Pyrrolo[2,3-d]pyrimidine compounds as Janus kinase inhibitors. (grant date: 21 January 2014).
172. Brown, M.F. (US); Fenwick, A.E. (US); Flanagan, M.E. (US); Gonzales, A. (US); Johnson, T.A. (US); et al. National Center for Biotechnology Information (2024). Patent US-9035074-B2, Pyrrolo[2,3-D]pyrimidine derivatives. (grant date: 19 May 2015).
173. McAllister, A. (IN); Murone, M. (IN); Sengupta, S. (IN); Shetty, S.J. (IN); et al. National Center for Biotechnology Information (2024). Patent US-8440679-B2, Bicyclic compounds and their uses as dual c-SRC/JAK inhibitors. (grant date: 21 May 2013).
174. Davies, A., Ioannidis, S., Lamb, M., Su, M., Wang, T., & Zhang, H. (2013). 9-(Pyrazol-3-yl)-9H-purine-2-amine and 3-(pyrazol-3-yl)-3H-imidazo[4,5-B] pyridin-5-amine derivatives and their use for the treatment of cancer. U.S. Patent No. US8486966B2. Filed March 13, 2012; granted July 16, 2013.
175. De Vicente Fidalgo, J. (US); Hermann, J.C. (US); Lemoine, R. (US); Li, H. (US); Lovey, A.J. (US); et al. Patent US-8618103-B2, Inhibitors of JAK for the treatment of autoimmune and inflammatory diseases. United States. Available online: https://pubchem.ncbi.nlm.nih.gov/patent/US-8618103-B2 (grant date: 31 December 2013).
176. Lin, S. (US); Lu, Z. (US); Spergel, S.H. (US); Tokarski, J.S. (US); Wu, H. (US); Yang, B.V. (US); et al. Pyrrolopyridazine JAK3 inhibitors and their use for the treatment of inflammatory and autoimmune diseases. Patent US-8921368-B2 (grant date: 30 December 2014).
177. Hynes, J.; Wu, H.; Kempson, J.; Duan, J.J.; Lu, Z.; Jiang, B.; Stachura, S.; Tokarski, J.S.; Sack, J.S.; Khan, J.A.; et al. Discovery of potent and efficacious pyrrolopyridazines as dual JAK1/3 inhibitors. Bioorg. Med. Chem. Lett. 2017, 27, 3101–3106. https://doi.org/10.1016/j.bmcl.2017.05.043.
178. Wrobleski, S.T. (US); Brown, G.D. (US); Doweyko, L.M. (US); Duan, J. (US); Guo, J. (US). National Center for Biotechnology Information (2024). Patent US-8921368-B2, Pyrrolopyridazine JAK3 inhibitors and their use for the treatment of inflammatory and autoimmune diseases. United States. (grant date: 30 December 2014).
179. Allen, S. (US); Andrews, S.W. (US); Condroski, K.R. (US); Haas, J. (US); Huang, L. (US); et al. National Center for Biotechnology Information (2024). Patent US-8791123-B2, Substituted pyrazolo[1,5-a]pyrimidine compounds as Trk kinase inhibitors. (grant date: 29 July 2014).

180. Noji, S. (JP); Shiozaki, M. (JP); Miura, T. (JP); Hara, Y. (JP); Yamanaka, H. (JP); Maeda, K. (JP); Hori, A. (JP); Inoue, M. (JP); Hase, Y. (JP). National Center for Biotechnology Information (2024). Patent US-8609647-B2, (grant date: 17 December 2013).
181. Combs, A. P., Sparks, R. B., Yue, E. W. T., Feng, H., & Bower, M. J. (2014). Macrocyclic compounds and their use as kinase inhibitors. U.S. Patent No. US8765727B2. Granted July 1, 2014.
182. Brubaker, J., Close, J. T., Jung, J., Martinez, M., & White, C. (2016). Acyclic cyanoethylpyrazoles as Janus kinase inhibitors. U.S. Patent No. US9493441B2. Granted November 15, 2016.
183. Hansen, B.B.; Jepsen, T.H.; Larsen, M.; Sindet, R.; Vifian, T.; Burhardt, M.N.; Larsen, J.; Seitzberg, J.G.; Carnerup, M.A.; Jerre, A.; et al. Fragment-Based Discovery of Pyrazolopyridones as JAK1 Inhibitors with Excellent Subtype Selectivity. J. Med. Chem. 2020, 63, 7008–7032. https://doi.org/10.1021/acs.jmedchem.0c00359.
184. Vasbinder, M.M.; Alimzhanov, M.; Augustin, M.; Bebernitz, G.; Bell, K.; Chuaqui, C.; Deegan, T.; Ferguson, A.D.; Goodwin, K.; Huszar, D.; et al. Identification of azabenzimidazoles as potent JAK1 selective inhibitors. Bioorg. Med. Chem. Lett. 2016, 26, 60–67. https://doi.org/10.1016/j.bmcl.2015.11.031.
185. Stauffer, F.; Cowan-Jacob, S.W.; Scheufler, C.; Furet, P. Identification of a 5-[3-phenyl-(2-cyclic-ether)-methylether]-4-amino-pyrrolo[2,3-d]pyrimidine series of IGF-1R inhibitors. Bioorg. Med. Chem. Lett. 2016, 26, 2065–2067. https://doi.org/10.1016/j.bmcl.2016.02.074.
186. Wang, T.; Liu, X.; Hao, M.; Qiao, J.; Ju, C.; Xue, L.; Zhang, C. Design, synthesis and evaluation of pyrrolo[2,3-d]pyrimidine-phenylamide hybrids as potent Janus kinase 2 inhibitors. Bioorg. Med. Chem. Lett. 2016, 26, 2936–2941. https://doi.org/10.1016/j.bmcl.2016.04.027.
187. Kim, H.; Kim, M.K.; Choo, H.; Chong, Y. Novel JAK1-selective benzimidazole inhibitors with enhanced membrane permeability. Bioorg. Med. Chem. Lett. 2016, 26, 3213–3215. https://doi.org/10.1016/j.bmcl.2016.05.078.
188. Cee, V.J.; Albrecht, B.K.; Geuns-Meyer, S.; Hughes, P.; Bellon, S.; Bready, J.; Caenepeel, S.; Chaffee, S.C.; Coxon, A.; Emery, M.; et al. Alkynylpyrimidine amide derivatives as potent, selective, and orally active inhibitors of Tie-2 kinase. J. Med. Chem. 2007, 50, 627–640. https://doi.org/10.1021/jm061112p.
189. Martin, M.W.; Newcomb, J.; Nunes, J.J.; McGowan, D.C.; Armistead, D.M.; Boucher, C.; Buchanan, J.L.; Buckner, W.; Chai, L.; Elbaum, D.; et al. Novel 2-aminopyrimidine carbamates as potent and orally active inhibitors of Lck: Synthesis, SAR, and in vivo antiinflammatory activity. J. Med. Chem. 2006, 49, 4981–4991. https://doi.org/10.1021/jm060435i.
190. Phuangsawai, O.; Beswick, P.; Ratanabunyong, S.; Tabtimmai, L.; Suphakun, P.; Obounchoey, P.; Srisook, P.; Horata, N.; Chuckowree, I.; Hannongbua, S.; et al. Evaluation of the anti-malarial activity and cytotoxicity of 2,4-diamino-pyrimidine-based kinase inhibitors. Eur. J. Med. Chem. 2016, 124, 896–905. https://doi.org/10.1016/j.ejmech.2016.08.055.
191. Hou, W.; Ren, Y.; Zhang, Z.; Sun, H.; Ma, Y.; Yan, B. Novel quinazoline derivatives bearing various 6-benzamide moieties as highly selective and potent EGFR inhibitors. Bioorg. Med. Chem. 2018, 26, 1740–1750. https://doi.org/10.1016/j.bmc.2018.02.022.
192. Narayan, S.; Ramisetti, S.; Jaiswal, A.S.; Law, B.K.; Singh-Pillay, A.; Singh, P.; Amin, S.; Sharma, A.K. ASR352, A potent anticancer agent: Synthesis, preliminary SAR, and biological activities against colorectal cancer bulk, 5-fluorouracil/oxaliplatin resistant and stem cells. Eur. J. Med. Chem. 2019, 161, 456–467. https://doi.org/10.1016/j.ejmech.2018.10.052.
193. Vankayalapati, H. (US); Yerramreddy, V.K. (US); Gangireddy, P. (US); Appalaneni, R.P. (US); et al. National Center for Biotechnology Information (2024). PubChem Patent Summary for US-9206188-B2, Substituted pyrrolo[2,3-b]pyridines as ITK and JAK inhibitors. United States. Available online: https://pubchem.ncbi.nlm.nih.gov/patent/US-9206188-B2 (grant date: 8 December 2015)
194. Wang, Y.; Huang, W.; Xin, M.; Chen, P.; Gui, L.; Zhao, X.; Tang, F.; Wang, J.; Liu, F. Identification of 4-(2-furanyl)pyrimidin-2-amines as Janus kinase 2 inhibitors. Bioorg. Med. Chem. 2017, 25, 75–83. https://doi.org/10.1016/j.bmc.2016.10.011.
195. Batt, D.G.; Bertrand, M.B.; Delucca, G.; Galella, M.A.; Ko, S.S. (2016). Substituted tetrahydrocarbazole and carbazole carboxamide compounds. United States Patent US-9334290-B2, granted 10 May 2016. Assignee: Bristol Myers Squibb Co. Available online: https://patents.google.com/patent/US9334290B2.
196. Hayashi, K.; Watanabe, T.; Toyama, K.; Kamon, J.; Minami, M. (2015). Substituted pyrrolo[2,3-h][1,6]naphthyridines and compositions thereof as JAK inhibitors. United States Patent US-9216999-B2, granted 22 December 2015. Assignee(s): Hayashi Keishi, Watanabe Tsuneo, Toyama Koji, Kamon Junji, Minami Masataka. Available online: https://patents.google.com/patent/US9216999B2.
197. Yang, B.V.; Brown, G.D.; Gupta, A.K.; Pitts, W.J.; et al. (2016). National Center for Biotechnology Information (2024). PubChem Patent Summary for US-9428511-B2, Imidazopyridazine JAK3 inhibitors and their use for the treatment of inflammatory and autoimmune diseases. United States. Available online: https://pubchem.ncbi.nlm.nih.gov/patent/US-9428511-B2 (grant date: 30 August 2016).

198. Czodrowski, P.; Mallinger, A.; Wienke, D.; Esdar, C.; Pöschke, O.; Busch, M.; Rohdich, F.; Eccles, S.A.; Ortiz-Ruiz, M.J.; Schneider, R.; et al. Structure-Based Optimization of Potent, Selective, and Orally Bioavailable CDK8 Inhibitors Discovered by High-Throughput Screening. J. Med. Chem. 2016, 59, 9337–9349. https://doi.org/10.1021/acs.jmedchem.6b00597.
199. Clark, M.P.; George, K.M.; Bookland, R.G.; Chen, J.; Laughlin, S.K.; Thakur, K.D.; Lee, W.; Davis, J.R.; Cabrera, E.J.; Brugel, T.A.; et al. Development of new pyrrolopyrimidine-based inhibitors of Janus kinase 3 (JAK3). Bioorg. Med. Chem. Lett. 2007, 17, 1250–1253. https://doi.org/10.1016/j.bmcl.2006.12.018.
200. Mitton-Fry, M.J.; Berlinski, P.J.; Birchmeier, M.J.; Bowman, J.W.; Gonzales, A.J. (2015). National Center for Biotechnology Information (2024). PubChem Patent Summary for US-9161939-B2, Pyrrolo[2,3-d]pyrimidine compounds. United States. Available online: https://pubchem.ncbi.nlm.nih.gov/patent/US-9161939-B2 (grant date: 20 October 2015)
201. Smaill, J.B.; Gonzales, A.J.; Spicer, J.A.; Lee, H.; Reed, J.E.; Sexton, K.; Althaus, I.W.; Zhu, T.; Black, S.L.; Blaser, A.; et al. Tyrosine Kinase Inhibitors. 20. Optimization of Substituted Quinazoline and Pyrido[3,4-d]pyrimidine Derivatives as Orally Active, Irreversible Inhibitors of the Epidermal Growth Factor Receptor Family. J. Med. Chem. 2016, 59, 8103–8124. https://doi.org/10.1021/acs.jmedchem.6b00883.
202. Huang, Taisheng; Xue, Chu-Biao; Li, Hui-Yin; Li, Qun. (2014). Piperidin-4-yl azetidine derivatives as JAK1 inhibitors. United States Patent US-8765734-B2, granted 1 July 2014. Available online: https://patents.google.com/patent/US8765734B2.
203. Takahashi, K. (JP); Watanabe, T. (JP); Hayashi, K. (JP); Kurihara, K. (JP); Nakamura, T. (JP); Yamamoto, A. (JP); Nishimura, T. (JP); Kamiyama, T. (JP); Hidaka, Y. (JP). National Center for Biotechnology Information (2024). PubChem Patent Summary for US-9475813-B2, Tricyclic pyrrolopyridine compound, and JAK inhibitor. United States. Available online: https://pubchem.ncbi.nlm.nih.gov/patent/US-9475813-B2 (grant date: 25 October 2016) 02/08.
204. Xi, Ning (US); Li, Minxiong (CN); Hu, Haiyang (CN); Dai, Weilong (CN). National Center for Biotechnology Information (2024). PubChem Patent Summary for US-9403801-B2, Substituted heteroaryl compounds and methods of use. United States. Available online: https://pubchem.ncbi.nlm.nih.gov/patent/US-9403801-B2 (grant date: 2 August 2016).
205. Goldstein, David M.; Brameld, Kenneth Albert; Verner, Erik. National Center for Biotechnology Information (2024). PubChem Patent Summary for US-9187487-B2, Azaindole derivatives as tyrosine kinase inhibitors. United States. Available online: https://pubchem.ncbi.nlm.nih.gov/patent/US-9187487-B2 (grant date: 17 November 2015).
206. Ge, Y.; Jin, Y.; Wang, C.; Zhang, J.; Tang, Z.; Peng, J.; Liu, K.; Li, Y.; Zhou, Y.; Ma, X. Discovery of Novel Bruton's Tyrosine Kinase (BTK) Inhibitors Bearing a. ACS Med. Chem. Lett. 2016, 7, 1050–1055. https://doi.org/10.1021/acsmedchemlett.6b00235.
207. Li, Yun-Long (US); Rodgers, James D. (US). National Center for Biotechnology Information (2024). PubChem Patent Summary for US-9216984-B2, 3-[4-(7H-pyrrolo[2,3-d]pyrimidin-4-yl)-1H-pyrazol-1-yl]octanenitrile and heptanenitrile as JAK inhibitors. United States. Available online: https://pubchem.ncbi.nlm.nih.gov/patent/US-9216984-B2 (grant date: 22 December 2015).
208. Su, Wei-Guo (CN); Deng, Wei (CN); Li, Jinshui (CN); Ji, Jianguo (CN). National Center for Biotechnology Information (2024). PubChem Patent Summary for US-9346810-B2, Pyrrolopyrimidine compounds and uses thereof. United States. Available online: https://pubchem.ncbi.nlm.nih.gov/patent/US-9346810-B2 (grant date: 24 May 2016).
209. Brubaker, Jason; Childers, Matthew Lloyd; Christopher, Matthew; Close, Joshua T.; Katz, Jason David et al. (US). National Center for Biotechnology Information (2024). PubChem Patent Summary for US-9328099-B2, Cyanomethylpyrazole carboxamides as Janus kinase inhibitors. United States. Available online: https://pubchem.ncbi.nlm.nih.gov/patent/US-9328099-B2 (grant date: 3 May 2016)
210. Reddy, E Premkumar; Reddy, M V Ramana (US). Temple University—Of the Commonwealth System of Higher Education (US) (2014). Substituted pyrido[2,3-d]pyrimidin-7(8H)-ones and therapeutic uses thereof. (grant date: 18 November 2014).
211. Lam, B.; Arikawa, Y.; Cramlett, J.; Dong, Q.; de Jong, R.; Feher, V.; Grimshaw, C.E.; Farrell, P.J.; Hoffman, I.D.; Jennings, A.; et al. Discovery of TAK-659 an orally available investigational inhibitor of Spleen Tyrosine Kinase (SYK). Bioorg. Med. Chem. Lett. 2016, 26, 5947–5950. https://doi.org/10.1016/j.bmcl.2016.10.087.
212. Blomgren, P.; Chandrasekhar, J.; Di Paolo, J.A.; Fung, W.; Geng, G.; Ip, C.; Jones, R.; Kropf, J.E.; Lansdon, E.B.; Lee, S.; et al. Discovery of Lanraplenib (GS-9876): A Once-Daily Spleen Tyrosine Kinase Inhibitor for Autoimmune Diseases. ACS Med. Chem. Lett. 2020, 11, 506–513. https://doi.org/10.1021/acsmedchemlett.9b00621.
213. Abdel-Magid, A.F. Janus-Associated Kinase 1 (JAK1) Inhibitors as Potential Treatment for Immune Disorders. ACS Med. Chem. Lett. 2017, 8, 598–600. https://doi.org/10.1021/acsmedchemlett.7b00209.
214. Kempson, J.; Ovalle, D.; Guo, J.; Wrobleski, S.T.; Lin, S.; Spergel, S.H.; Duan, J.J.; Jiang, B.; Lu, Z.; Das, J.; et al. Discovery of highly potent, selective, covalent inhibitors of JAK3. Bioorg. Med. Chem. Lett. 2017, 27, 4622–4625. https://doi.org/10.1016/j.bmcl.2017.09.023.

215. Hanan, E.J.; Liang, J.; Wang, X.; Blake, R.A.; Blaquiere, N.; Staben, S.T. Monomeric Targeted Protein Degraders. J. Med. Chem. 2020, 63, 11330–11361. https://doi.org/10.1021/acs.jmedchem.0c00093.
216. Brameld, K.A.; Owens, T.D.; Verner, E.; Venetsanakos, E.; Bradshaw, J.M.; Phan, V.T.; Tam, D.; Leung, K.; Shu, J.; LaStant, J.; et al. Discovery of the Irreversible Covalent FGFR Inhibitor 8-(3-(4-Acryloylpiperazin-1-yl)propyl)-6-(2,6-dichloro-3,5-dimethoxyphenyl)-2-(methylamino)pyrido[2,3-d]pyrimidin-7(8H)-one (PRN1371) for the Treatment of Solid Tumors. J. Med. Chem. 2017, 60, 6516–6527. https://doi.org/10.1021/acs.jmedchem.7b00360.
217. Frankfurt donated chemical probe project: TP-030-2 was donated by Takeda. Website: https://www.sgc-ffm.uni-frankfurt.de/#!specificprobeoverview/TP-030-2. Control: TP-030n. References: 1. Yoshikawa, Masato, Morihisa Saitoh, Taisuke Katoh, Tomohiro Seki, Simone V. Bigi, Yuji Shimizu, Tsuyoshi Ishii, Takuro Okai, Masako Kuno, Harumi Hattori, Etsuro Watanabe, Kumar S. Saikatendu, Hua Zou, Masanori Nakakariya, Takayuki Tatamiya, Yoshihisa Nakada, and Takatoshi Yogo. 2018. 'Discovery of 7-Oxo-2,4,5,7-Tetrahydro-6 H-Pyrazolo[3,4- c]Pyridine Derivatives as Potent, Orally Available, and Brain-Penetrating Receptor Interacting Protein 1 (RIP1) Kinase Inhibitors: Analysis of Structure-Kinetic Relationships'. Journal of Medicinal Chemistry 61(6):2384–2409. PMID: 29485864 (Compound 60).<.
218. Data for DCP probe TP-030-2. Available online: https://www.ebi.ac.uk/chembl/explore/document/CHEMBL4507326 (accessed on 2nd February 2023).
219. Yao, L.; Mustafa, N.; Tan, E.C.; Poulsen, A.; Singh, P.; Duong-Thi, M.D.; Lee, J.X.T.; Ramanujulu, P.M.; Chng, W.J.; Yen, J.J.Y.; et al. Design and Synthesis of Ligand Efficient Dual Inhibitors of Janus Kinase (JAK) and Histone Deacetylase (HDAC) Based on Ruxolitinib and Vorinostat. J. Med. Chem. 2017, 60, 8336–8357. https://doi.org/10.1021/acs.jmedchem.7b00678.
220. Fischer, T.; Krüger, T.; Najjar, A.; Totzke, F.; Schächtele, C.; Sippl, W.; Ritter, C.; Hilgeroth, A. Discovery of novel substituted benzo-anellated 4-benzylamino pyrrolopyrimidines as dual EGFR and VEGFR2 inhibitors. Bioorg. Med. Chem. Lett. 2017, 27, 2708–2712. https://doi.org/10.1016/j.bmcl.2017.04.053.
221. Vazquez, M.L.; Kaila, N.; Strohbach, J.W.; Trzupek, J.D.; Brown, M.F.; Flanagan, M.E.; Mitton-Fry, M.J.; Johnson, T.A.; TenBrink, R.E.; Arnold, E.P.; et al. Identification of N-{cis-3-[Methyl(7H-pyrrolo[2,3-d]pyrimidin-4-yl)amino]cyclobutyl}propane-1-sulfonamide (PF-04965842): A Selective JAK1 Clinical Candidate for the Treatment of Autoimmune Diseases. J. Med. Chem. 2018, 61, 1130–1152. https://doi.org/10.1021/acs.jmedchem.7b01598.
222. Brubaker, J., Close, J., Siu, T., Smith, G. F., & Torres, L. E. (2016). Pyrazole carboxamides as Janus kinase inhibitors. U.S. Patent No. US9394282B2. Granted July 19, 2016.
223. Juillerat-Jeanneret, L.; Aubert, J.D.; Mikulic, J.; Golshayan, D. Fibrogenic Disorders in Human Diseases: From Inflammation to Organ Dysfunction. J. Med. Chem. 2018, 61, 9811–9840. https://doi.org/10.1021/acs.jmedchem.8b00294.
224. Huang, Y.; Dong, G.; Li, H.; Liu, N.; Zhang, W.; Sheng, C. Discovery of Janus Kinase 2 (JAK2) and Histone Deacetylase (HDAC) Dual Inhibitors as a Novel Strategy for the Combinational Treatment of Leukemia and Invasive Fungal Infections. J. Med. Chem. 2018, 61, 6056–6074. https://doi.org/10.1021/acs.jmedchem.8b00393.
225. Grimster, N.P.; Anderson, E.; Alimzhanov, M.; Bebernitz, G.; Bell, K.; Chuaqui, C.; Deegan, T.; Ferguson, A.D.; Gero, T.; Harsch, A.; et al. Discovery and Optimization of a Novel Series of Highly Selective JAK1 Kinase Inhibitors. J. Med. Chem. 2018, 61, 5235–5244. https://doi.org/10.1021/acs.jmedchem.8b00076.
226. Chough, C.; Joung, M.; Lee, S.; Lee, J.; Kim, J.H.; Kim, B.M. Development of selective inhibitors for the treatment of rheumatoid arthritis: (R)-3-(3-(Methyl(7H-pyrrolo[2,3-d]pyrimidin-4-yl)amino)pyrrolidin-1-yl)-3-oxopropanenitrile as a JAK1-selective inhibitor. Bioorg. Med. Chem. 2018, 26, 1495–1510. https://doi.org/10.1016/j.bmc.2018.01.021.
227. Yu, T.; Zhang, Y.; Kerekes, A.D.; Tagat, J.R.; Doll, R.J.; Xiao, Y.; Esposite, S.; Hruza, A.; Belanger, D.B.; Voss, M.; et al. Discovery of a highly potent orally bioavailable imidazo-[1, 2-a]pyrazine Aurora inhibitor. Bioorg. Med. Chem. Lett. 2018, 28, 1397–1403. https://doi.org/10.1016/j.bmcl.2018.02.037.
228. El-Gamal, M.I.; Al-Ameen, S.K.; Al-Koumi, D.M.; Hamad, M.G.; Jalal, N.A.; Oh, C.H. Recent Advances of Colony-Stimulating Factor-1 Receptor (CSF-1R) Kinase and Its Inhibitors. J. Med. Chem. 2018, 61, 5450–5466. https://doi.org/10.1021/acs.jmedchem.7b00873.
229. Wang, Y.; Huang, W.; Xin, M.; Chen, P.; Gui, L.; Zhao, X.; Zhu, X.; Luo, H.; Cong, X.; Wang, J.; et al. Discovery of potent anti-inflammatory 4-(4,5,6,7-tetrahydrofuro[3,2-c]pyridin-2-yl) pyrimidin-2-amines for use as Janus kinase inhibitors. Bioorg. Med. Chem. 2019, 27, 2592–2597. https://doi.org/10.1016/j.bmc.2019.03.048.
230. Zheng, J.; Wu, J.; Ding, X.; Shen, H.C.; Zou, G. Small molecule approaches to treat autoimmune and inflammatory diseases (Part I): Kinase inhibitors. Bioorg. Med. Chem. Lett. 2021, 38, 127862. https://doi.org/10.1016/j.bmcl.2021.127862.

231. Yao, L.; Ramanujulu, P.M.; Poulsen, A.; Ohlson, S.; Dymock, B.W. Merging of ruxolitinib and vorinostat leads to highly potent inhibitors of JAK2 and histone deacetylase 6 (HDAC6). Bioorg. Med. Chem. Lett. 2018, 28, 2636–2640. https://doi.org/10.1016/j.bmcl.2018.06.037.
232. Liu, Q.; Batt, D.G.; Chaudhry, C.; Lippy, J.S.; Pattoli, M.A.; Surti, N.; Xu, S.; Carter, P.H.; Burke, J.R.; Tino, J.A. Conversion of carbazole carboxamide based reversible inhibitors of Bruton's tyrosine kinase (BTK) into potent, selective irreversible inhibitors in the carbazole, tetrahydrocarbazole, and a new 2,3-dimethylindole series. Bioorg. Med. Chem. Lett. 2018, 28, 3080–3084. https://doi.org/10.1016/j.bmcl.2018.07.041.
233. Pippione, A.C.; Sainas, S.; Federico, A.; Lupino, E.; Piccinini, M.; Kubbutat, M.; Contreras, J.M.; Morice, C.; Barge, A.; Ducime, A.; et al. -Acetyl-3-aminopyrazoles block the non-canonical NF-kB cascade by selectively inhibiting NIK. Medchemcomm 2018, 9, 963–968. https://doi.org/10.1039/c8md00068a.
234. Yin, L.; Li, H.; Liu, W.; Yao, Z.; Cheng, Z.; Zhang, H.; Zou, H. A highly potent CDK4/6 inhibitor was rationally designed to overcome blood brain barrier in gliobastoma therapy. Eur. J. Med. Chem. 2018, 144, 1–28. https://doi.org/10.1016/j.ejmech.2017.12.003.
235. Chough, C.; Lee, S.; Joung, M.; Lee, J.; Kim, J.H.; Kim, B.M. Design, synthesis and evaluation of (Medchemcomm 2018, 9, 477–489. https://doi.org/10.1039/c7md00568g.
236. Chu-Farseeva, Y.Y.; Mustafa, N.; Poulsen, A.; Tan, E.C.; Yen, J.J.Y.; Chng, W.J.; Dymock, B.W. Design and synthesis of potent dual inhibitors of JAK2 and HDAC based on fusing the pharmacophores of XL019 and vorinostat. Eur. J. Med. Chem. 2018, 158, 593–619. https://doi.org/10.1016/j.ejmech.2018.09.024.
237. Zhang, K.; Ye, K.; Tang, H.; Qi, Z.; Wang, T.; Mao, J.; Zhang, X.; Jiang, S. Development and Therapeutic Implications of Tyrosine Kinase 2 Inhibitors. J. Med. Chem. 2023, 66, 4378–4416. https://doi.org/10.1021/acs.jmedchem.2c01800.
238. Henry, S.P.; Jorgensen, W.L. Progress on the Pharmacological Targeting of Janus Pseudokinases. J. Med. Chem. 2023, 66, 10959–10990. https://doi.org/10.1021/acs.jmedchem.3c00926.
239. Ge, Y.; Wang, C.; Song, S.; Huang, J.; Liu, Z.; Li, Y.; Meng, Q.; Zhang, J.; Yao, J.; Liu, K.; et al. Identification of highly potent BTK and JAK3 dual inhibitors with improved activity for the treatment of B-cell lymphoma. Eur. J. Med. Chem. 2018, 143, 1847–1857. https://doi.org/10.1016/j.ejmech.2017.10.080.
240. Moslin, R.; Gardner, D.; Santella, J.; Zhang, Y.; Duncia, J.V.; Liu, C.; Lin, J.; Tokarski, J.S.; Strnad, J.; Pedicord, D.; et al. Identification of imidazo[1,2-. Medchemcomm 2017, 8, 700–712. https://doi.org/10.1039/c6md00560h.
241. Noji, S.; Hara, Y.; Miura, T.; Yamanaka, H.; Maeda, K.; Hori, A.; Yamamoto, H.; Obika, S.; Inoue, M.; Hase, Y.; et al. Discovery of a Janus Kinase Inhibitor Bearing a Highly Three-Dimensional Spiro Scaffold: JTE-052 (Delgocitinib) as a New Dermatological Agent to Treat Inflammatory Skin Disorders. J. Med. Chem. 2020, 63, 7163–7185. https://doi.org/10.1021/acs.jmedchem.0c00450.
242. Li, Y.; Ye, T.; Xu, L.; Dong, Y.; Luo, Y.; Wang, C.; Han, Y.; Chen, K.; Qin, M.; Liu, Y.; et al. Discovery of 4-piperazinyl-2-aminopyrimidine derivatives as dual inhibitors of JAK2 and FLT3. Eur. J. Med. Chem. 2019, 181, 111590. https://doi.org/10.1016/j.ejmech.2019.111590.
243. EUbOPEN. Selectivity Literature for EUbOPEN Chemogenomic Library. Available online: https://doi.org/10.6019/CHEMBL5465560 (accessed on 2nd July 2023).
244. Miao, Q.; Ma, K.; Chen, D.; Wu, X.; Jiang, S. Targeting tropomyosin receptor kinase for cancer therapy. Eur. J. Med. Chem. 2019, 175, 129–148. https://doi.org/10.1016/j.ejmech.2019.04.053.
245. Moslin, R.; Zhang, Y.; Wrobleski, S.T.; Lin, S.; Mertzman, M.; Spergel, S.; Tokarski, J.S.; Strnad, J.; Gillooly, K.; McIntyre, K.W.; et al. Identification of N-Methyl Nicotinamide and N-Methyl Pyridazine-3-Carboxamide Pseudokinase Domain Ligands as Highly Selective Allosteric Inhibitors of Tyrosine Kinase 2 (TYK2). J. Med. Chem. 2019, 62, 8953–8972. https://doi.org/10.1021/acs.jmedchem.9b00443.
246. Bryan, M.C.; Rajapaksa, N.S. Kinase Inhibitors for the Treatment of Immunological Disorders: Recent Advances. J. Med. Chem. 2018, 61, 9030–9058. https://doi.org/10.1021/acs.jmedchem.8b00667.
247. Zhang, C.; Qi, W.; Li, Y.; Tang, M.; Yang, T.; Liu, K.; Chen, Y.; Deng, D.; Xiang, M.; Chen, L. Discovery of 3-(4-(2-((1H-Indol-5-yl)amino)-5-fluoropyrimidin-4-yl)-1H-pyrazol-1-yl)propanenitrile Derivatives as Selective TYK2 Inhibitors for the Treatment of Inflammatory Bowel Disease. J. Med. Chem. 2021, 64, 1966–1988. https://doi.org/10.1021/acs.jmedchem.0c01468.
248. Shi, C.; Wang, Q.; Liao, X.; Ge, H.; Huo, G.; Zhang, L.; Chen, N.; Zhai, X.; Hong, Y.; Wang, L.; et al. Discovery of 6-(2-(dimethylamino)ethyl)-N-(5-fluoro-4-(4-fluoro-1-isopropyl-2-methyl-1H-benzo[d]imidazole-6-yl)pyrimidin-2-yl)-5,6,7,8-tetrahydro-1,6-naphthyridin-2-amine as a highly potent cyclin-dependent kinase 4/6 inhibitor for treatment of cancer. Eur. J. Med. Chem. 2019, 178, 352–364. https://doi.org/10.1016/j.ejmech.2019.06.005.

249. Liang, X.; Zang, J.; Li, X.; Tang, S.; Huang, M.; Geng, M.; Chou, C.J.; Li, C.; Cao, Y.; Xu, W.; et al. Discovery of Novel Janus Kinase (JAK) and Histone Deacetylase (HDAC) Dual Inhibitors for the Treatment of Hematological Malignancies. J. Med. Chem. 2019, 62, 3898–3923. https://doi.org/10.1021/acs.jmedchem.8b01597.
250. Liang, X.; Zang, J.; Zhu, M.; Gao, Q.; Wang, B.; Xu, W.; Zhang, Y. Design, Synthesis, and Antitumor Evaluation of 4-Amino-(1. ACS Med. Chem. Lett. 2016, 7, 950–955. https://doi.org/10.1021/acsmedchemlett.6b00247.
251. Bach, J.; Eastwood, P.; González, J.; Gómez, E.; Alonso, J.A.; Fonquerna, S.; Lozoya, E.; Orellana, A.; Maldonado, M.; Calaf, E.; et al. Identification of 2-Imidazopyridine and 2-Aminopyridone Purinones as Potent Pan-Janus Kinase (JAK) Inhibitors for the Inhaled Treatment of Respiratory Diseases. J. Med. Chem. 2019, 62, 9045–9060. https://doi.org/10.1021/acs.jmedchem.9b00533.
252. Tang, G.; Liu, L.; Wang, X.; Pan, Z. Discovery of 7H-pyrrolo[2,3-d]pyrimidine derivatives as selective covalent irreversible inhibitors of interleukin-2-inducible T-cell kinase (Itk). Eur. J. Med. Chem. 2019, 173, 167–183. https://doi.org/10.1016/j.ejmech.2019.03.055.
253. CoE, J.W.; Dehnhardt, C.M.; Jones, P.; Korturn, S.W.; Sabnis, Y.A.; Wakenhut, F.M.; Whitlock, G.A. JAK inhibitors for the treatment of inflammatory diseases. U.S. Patent US8895544B2, 25 November 2014.
254. Garton, N.S.; Barker, M.D.; Davis, R.P.; Douault, C.; Hooper-Greenhill, E.; Jones, E.; Lewis, H.D.; Liddle, J.; Lugo, D.; McCleary, S.; et al. Optimisation of a novel series of potent and orally bioavailable azanaphthyridine SYK inhibitors. Bioorg. Med. Chem. Lett. 2016, 26, 4606–4612. https://doi.org/10.1016/j.bmcl.2016.08.070.
255. Leonard, K.A.; Madge, L.A.; Krawczuk, P.J.; Wang, A.; Kreutter, K.D.; Bacani, G.M.; Chai, W.; Smith, R.C.; Tichenor, M.S.; Harris, M.C.; et al. Discovery of a Gut-Restricted JAK Inhibitor for the Treatment of Inflammatory Bowel Disease. J. Med. Chem. 2020, 63, 2915–2929. https://doi.org/10.1021/acs.jmedchem.9b01439.
256. Shi, L.; Zhong, Z.; Li, X.; Zhou, Y.; Pan, Z. Discovery of an Orally Available Janus Kinase 3 Selective Covalent Inhibitor. J. Med. Chem. 2019, 62, 1054–1066. https://doi.org/10.1021/acs.jmedchem.8b01823.
257. National Center for Biotechnology Information (2024). PubChem Substance Record for SID 395763535, S., Source: PATENTSCOPE (WIPO). Retrieved December 18, 2024 from https://pubchem.ncbi.nlm.nih.gov/substance/395763535 .
258. Yuan, X.; Wu, H.; Bu, H.; Zhou, J.; Zhang, H. Targeting the immunity protein kinases for immuno-oncology. Eur. J. Med. Chem. 2019, 163, 413–427. https://doi.org/10.1016/j.ejmech.2018.11.072.
259. Ritzén, A.; Sørensen, M.D.; Dack, K.N.; Greve, D.R.; Jerre, A.; Carnerup, M.A.; Rytved, K.A.; Bagger-Bahnsen, J. Fragment-Based Discovery of 6-Arylindazole JAK Inhibitors. ACS Med. Chem. Lett. 2016, 7, 641–646. https://doi.org/10.1021/acsmedchemlett.6b00087.
260. Fensome, A.; Ambler, C.M.; Arnold, E.; Banker, M.E.; Brown, M.F.; Chrencik, J.; Clark, J.D.; Dowty, M.E.; Efremov, I.V.; Flick, A.; et al. Dual Inhibition of TYK2 and JAK1 for the Treatment of Autoimmune Diseases: Discovery of ((S)-2,2-Difluorocyclopropyl)((1 R,5 S)-3-(2-((1-methyl-1 H-pyrazol-4-yl)amino)pyrimidin-4-yl)-3,8-diazabicyclo[3.2.1]octan-8-yl)methanone (PF-06700841). J. Med. Chem. 2018, 61, 8597–8612. https://doi.org/10.1021/acs.jmedchem.8b00917.
261. Chen, P.; Norris, D.; Das, J.; Spergel, S.H.; Wityak, J.; Leith, L.; Zhao, R.; Chen, B.C.; Pitt, S.; Pang, S.; et al. Discovery of novel 2-(aminoheteroaryl)-thiazole-5-carboxamides as potent and orally active Src-family kinase p56(Lck) inhibitors. Bioorg Med Chem Lett 2004, 14, 6061-6066, doi:10.1016/j.bmcl.2004.09.093.
262. Yang, T.; Hu, M.; Qi, W.; Yang, Z.; Tang, M.; He, J.; Chen, Y.; Bai, P.; Yuan, X.; Zhang, C.; et al. Discovery of Potent and Orally Effective Dual Janus Kinase 2/FLT3 Inhibitors for the Treatment of Acute Myelogenous Leukemia and Myeloproliferative Neoplasms. J. Med. Chem. 2019, 62, 10305–10320. https://doi.org/10.1021/acs.jmedchem.9b01348.
263. National Center for Biotechnology Information (2024). PubChem Bioassay Record for AID 1791642, N.S.F.J., Source: ChEMBL. Retrieved December 25, 2024 from https://pubchem.ncbi.nlm.nih.gov/bioassay/1791642 .
264. Lee, S.M.; Yoon, K.B.; Lee, H.J.; Kim, J.; Chung, Y.K.; Cho, W.J.; Mukai, C.; Choi, S.; Kang, K.W.; Han, S.Y.; et al. The discovery of 2,5-isomers of triazole-pyrrolopyrimidine as selective Janus kinase 2 (JAK2) inhibitors versus JAK1 and JAK3. Bioorg. Med. Chem. 2016, 24, 5036–5046. https://doi.org/10.1016/j.bmc.2016.08.008.
265. Luo, Z.; Wang, L.; Fu, Z.; Shuai, B.; Luo, M.; Hu, G.; Chen, J.; Sun, J.; Wang, J.; Li, J.; et al. Discovery and optimization of selective RET inhibitors via scaffold hopping. Bioorg. Med. Chem. Lett. 2021, 47, 128149. https://doi.org/10.1016/j.bmcl.2021.128149.
266. Egyed, A.; Bajusz, D.; Keserű, G.M. The impact of binding site waters on the activity/selectivity trade-off of Janus kinase 2 (JAK2) inhibitors. Bioorg. Med. Chem. 2019, 27, 1497–1508. https://doi.org/10.1016/j.bmc.2019.02.029. 10.1016/j.bmcl.2015.09.011
267. Chen, Y.; Li, H.; Yen, R.; Heckrodt, T.J.; McMurtrie, D.; Singh, R.; Taylor, V.; Masuda, E.S.; Park, G.; Payan, D.G. Optimization of Pyrimidine Compounds as Potent JAK1 Inhibitors and the Discovery of R507 as a Clinical Candidate. ACS Med. Chem. Lett. 2022, 13, 1805–1811. https://doi.org/10.1021/acsmedchemlett.2c00411.

268. Liu, L.; Norman, M.H.; Lee, M.; Xi, N.; Siegmund, A.; Boezio, A.A.; Booker, S.; Choquette, D.; D'Angelo, N.D.; Germain, J.; et al. Structure-based design of novel class II c-Met inhibitors: 2. SAR and kinase selectivity profiles of the pyrazolone series. J. Med. Chem. 2012, 55, 1868–1897. https://doi.org/10.1021/jm201331s.
269. Schlapbach, A.; Feifel, R.; Hawtin, S.; Heng, R.; Koch, G.; Moebitz, H.; Revesz, L.; Scheufler, C.; Velcicky, J.; Waelchli, R.; et al. Pyrrolo-pyrimidones: A novel class of MK2 inhibitors with potent cellular activity. Bioorg. Med. Chem. Lett. 2008, 18, 6142–6146. https://doi.org/10.1016/j.bmcl.2008.10.039.
270. Bauer, D.; Whittington, D.A.; Coxon, A.; Bready, J.; Harriman, S.P.; Patel, V.F.; Polverino, A.; Harmange, J.C. Evaluation of indazole-based compounds as a new class of potent KDR/VEGFR-2 inhibitors. Bioorg. Med. Chem. Lett. 2008, 18, 4844–4848. https://doi.org/10.1016/j.bmcl.2008.07.080.
271. Zhou, H.; McGowan, M.A.; Lipford, K.; Christopher, M.; Fradera, X.; Witter, D.; Lesburg, C.A.; Li, C.; Methot, J.L.; Lampe, J.; et al. Discovery and optimization of heteroaryl piperazines as potent and selective PI3Kδ inhibitors. Bioorg. Med. Chem. Lett. 2020, 30, 126715. https://doi.org/10.1016/j.bmcl.2019.126715.
272. Shah, R.R.; Redmond, J.M.; Mihut, A.; Menon, M.; Evans, J.P.; Murphy, J.A.; Bartholomew, M.A.; Coe, D.M. Hi-JAK-ing the ubiquitin system: The design and physicochemical optimisation of JAK PROTACs. Bioorg. Med. Chem. 2020, 28, 115326. https://doi.org/10.1016/j.bmc.2020.115326.
273. Zhu, Y.; Zheng, X.; Wang, C.; Sun, X.; Sun, H.; Ma, T.; Li, Y.; Liu, K.; Chen, L.; Ma, X. Synthesis and biological activity of thieno[3,2-d]pyrimidines as potent JAK3 inhibitors for the treatment of idiopathic pulmonary fibrosis. Bioorg. Med. Chem. 2020, 28, 115254. https://doi.org/10.1016/j.bmc.2019.115254.
274. Sasaki, Y.; Tokuhara, H.; Ohba, Y.; Okabe, A.; Nakayama, M.; Nakagawa, H.; Skene, R.; Hoffman, I.; Zou, H.; Yoshida, M. Efficient synthesis of tert-butyl 3-cyano-3-cyclopropyl-2-oxopyrrolidine-4-carboxylates: Highly functionalized 2-pyrrolidinone enabling access to novel macrocyclic Tyk2 inhibitors. Bioorg. Med. Chem. Lett. 2020, 30, 126963. https://doi.org/10.1016/j.bmcl.2020.126963.
275. Lu, K.; Wu, W.; Zhang, C.; Liu, Z.; Xiao, B.; Yuan, Z.; Li, A.; Chen, D.; Zhai, X.; Jiang, Y. Discovery of triazolo [1,5-a] pyridine derivatives as novel JAK1/2 inhibitors. Bioorg. Med. Chem. Lett. 2020, 30, 127225. https://doi.org/10.1016/j.bmcl.2020.127225.
276. Zhu, Y.; Ma, Y.; Zu, W.; Song, J.; Wang, H.; Zhong, Y.; Li, H.; Zhang, Y.; Gao, Q.; Kong, B.; et al. Identification of. J. Med. Chem. 2020, 63, 6748–6773. https://doi.org/10.1021/acs.jmedchem.0c00055.
277. Wang, X.; Xue, G.; Pan, Z. Design, synthesis and structure-activity relationship of indolylindazoles as potent and selective covalent inhibitors of interleukin-2 inducible T-cell kinase (ITK). Eur. J. Med. Chem. 2020, 187, 111918. https://doi.org/10.1016/j.ejmech.2019.111918.
278. Gerstenberger, B.S.; Ambler, C.; Arnold, E.P.; Banker, M.E.; Brown, M.F.; Clark, J.D.; Dermenci, A.; Dowty, M.E.; Fensome, A.; Fish, S.; et al. Discovery of Tyrosine Kinase 2 (TYK2) Inhibitor (PF-06826647) for the Treatment of Autoimmune Diseases. J. Med. Chem. 2020, 63, 13561–13577. https://doi.org/10.1021/acs.jmedchem.0c00948.
279. Zak, M.; Hanan, E.J.; Lupardus, P.; Brown, D.G.; Robinson, C.; Siu, M.; Lyssikatos, J.P.; Romero, F.A.; Zhao, G.; Kellar, T.; et al. Discovery of a class of highly potent Janus Kinase 1/2 (JAK1/2) inhibitors demonstrating effective cell-based blockade of IL-13 signaling. Bioorg. Med. Chem. Lett. 2019, 29, 1522–1531. https://doi.org/10.1016/j.bmcl.2019.04.008.
280. Baillache, D.J.; Unciti-Broceta, A. Recent developments in anticancer kinase inhibitors based on the pyrazolo[3,4-d]pyrimidine scaffold. RSC Med. Chem. 2020, 11, 1112–1135. https://doi.org/10.1039/d0md00227e.
281. Thoma, G.; Blanz, J.; Bühlmayer, P.; Drückes, P.; Kittelmann, M.; Smith, A.B.; van Eis, M.; Vangrevelinghe, E.; Zerwes, H.G.; Che, J.J.; et al. Syk inhibitors with high potency in presence of blood. Bioorg. Med. Chem. Lett. 2014, 24, 2278–2282. https://doi.org/10.1016/j.bmcl.2014.03.075.
282. Luo, L.; Jia, J.J.; Zhong, Q.; Zhong, X.; Zheng, S.; Wang, G.; He, L. Synthesis and anticancer activity evaluation of naphthalene-substituted triazole spirodienones. Eur. J. Med. Chem. 2021, 213, 113039. https://doi.org/10.1016/j.ejmech.2020.113039.
283. Yang, T.; Hu, M.; Chen, Y.; Xiang, M.; Tang, M.; Qi, W.; Shi, M.; He, J.; Yuan, X.; Zhang, C.; et al. -(Pyrimidin-2-yl)-1,2,3,4-tetrahydroisoquinolin-6-amine Derivatives as Selective Janus Kinase 2 Inhibitors for the Treatment of Myeloproliferative Neoplasms. J. Med. Chem. 2020, 63, 14921–14936. https://doi.org/10.1021/acs.jmedchem.0c01488.
284. Matheson, C.J.; Coxon, C.R.; Bayliss, R.; Boxall, K.; Carbain, B.; Fry, A.M.; Hardcastle, I.R.; Harnor, S.J.; Mas-Droux, C.; Newell, D.R.; et al. 2-Arylamino-6-ethynylpurines are cysteine-targeting irreversible inhibitors of Nek2 kinase. RSC Med. Chem. 2020, 11, 707–731. https://doi.org/10.1039/d0md00074d.
285. Liu, C.; Lin, J.; Langevine, C.; Smith, D.; Li, J.; Tokarski, J.S.; Khan, J.; Ruzanov, M.; Strnad, J.; Zupa-Fernandez, A.; et al. Discovery of BMS-986202: A Clinical Tyk2 Inhibitor that Binds to Tyk2 JH2. J. Med. Chem. 2021, 64, 677–694. https://doi.org/10.1021/acs.jmedchem.0c01698.

286. Zheng, Y.G.; Wang, J.A.; Meng, L.; Pei, X.; Zhang, L.; An, L.; Li, C.L.; Miao, Y.L. Design, synthesis, biological activity evaluation of 3-(4-phenyl-1H-imidazol-2-yl)-1H-pyrazole derivatives as potent JAK 2/3 and aurora A/B kinases multi-targeted inhibitors. Eur. J. Med. Chem. 2021, 209, 112934. https://doi.org/10.1016/j.ejmech.2020.112934.
287. Sanachai, K.; Aiebchun, T.; Mahalapbutr, P.; Seetaha, S.; Tabtimmai, L.; Maitarad, P.; Xenikakis, I.; Geronikaki, A.; Choowongkomon, K.; Rungrotmongkol, T. Discovery of novel JAK2 and EGFR inhibitors from a series of thiazole-based chalcone derivatives. RSC Med. Chem. 2021, 12, 430–438. https://doi.org/10.1039/d0md00436g.
288. Wu, L.; Zhang, C.; He, C.; Qian, D.; Lu, L.; Sun, Y.; Xu, M.; Zhuo, J.; Liu, P.C.C.; Klabe, R.; et al. Discovery of Pemigatinib: A Potent and Selective Fibroblast Growth Factor Receptor (FGFR) Inhibitor. J. Med. Chem. 2021, 64, 10666–10679. https://doi.org/10.1021/acs.jmedchem.1c00713.
289. Xu, P.; Shen, P.; Wang, H.; Qin, L.; Ren, J.; Sun, Q.; Ge, R.; Bian, J.; Zhong, Y.; Li, Z.; et al. Discovery of imidazopyrrolopyridines derivatives as novel and selective inhibitors of JAK2. Eur. J. Med. Chem. 2021, 218, 113394. https://doi.org/10.1016/j.ejmech.2021.113394.
290. Rao, D.; Li, H.; Ren, X.; Sun, Y.; Wen, C.; Zheng, M.; Huang, H.; Tang, W.; Xu, S. Discovery of a potent, selective, and covalent ZAP-70 kinase inhibitor. Eur. J. Med. Chem. 2021, 219, 113393. https://doi.org/10.1016/j.ejmech.2021.113393.
291. Howard, S.; Berdini, V.; Boulstridge, J.A.; Carr, M.G.; Cross, D.M.; Curry, J.; Devine, L.A.; Early, T.R.; Fazal, L.; Gill, A.L.; et al. Fragment-based discovery of the pyrazol-4-yl urea (AT9283), a multitargeted kinase inhibitor with potent aurora kinase activity. J. Med. Chem. 2009, 52, 379–388. https://doi.org/10.1021/jm800984v.
292. Pollard, J.R.; Mortimore, M. Discovery and development of aurora kinase inhibitors as anticancer agents. J. Med. Chem. 2009, 52, 2629–2651. https://doi.org/10.1021/jm8012129.
293. Yang, T.; Cui, X.; Tang, M.; Qi, W.; Zhu, Z.; Shi, M.; Yang, L.; Pei, H.; Zhang, W.; Xie, L.; et al. Identification of a Novel 2,8-Diazaspiro[4.5]decan-1-one Derivative as a Potent and Selective Dual TYK2/JAK1 Inhibitor for the Treatment of Inflammatory Bowel Disease. J. Med. Chem. 2022, 65, 3151–3172. https://doi.org/10.1021/acs.jmedchem.1c01137.
294. Liang, X.; Tang, S.; Liu, X.; Liu, Y.; Xu, Q.; Wang, X.; Saidahmatov, A.; Li, C.; Wang, J.; Zhou, Y.; et al. Discovery of Novel Pyrrolo[2,3-. J. Med. Chem. 2022, 65, 1243–1264. https://doi.org/10.1021/acs.jmedchem.0c02111.
295. Wellaway, C.R.; Baldwin, I.R.; Bamborough, P.; Barker, D.; Bartholomew, M.A.; Chung, C.W.; Dümpelfeld, B.; Evans, J.P.; Fazakerley, N.J.; Homes, P.; et al. Investigation of Janus Kinase (JAK) Inhibitors for Lung Delivery and the Importance of Aldehyde Oxidase Metabolism. J. Med. Chem. 2022, 65, 633–664. https://doi.org/10.1021/acs.jmedchem.1c01765.
296. Wu, S.; Liao, M.; Li, M.; Sun, M.; Xi, N.; Zeng, Y. Structure-based discovery of potent inhibitors of Axl: Design, synthesis, and biological evaluation. RSC Med. Chem. 2022, 13, 1246–1264. https://doi.org/10.1039/d2md00153e.
297. Mao, W.; Wu, H.; Guo, Q.; Zheng, X.; Wei, C.; Liao, Y.; Shen, L.; Mi, J.; Li, J.; Chen, S.; et al. Synthesis and evaluation of hydrazinyl-containing pyrrolo[2,3-d]pyrimidine series as potent, selective and oral JAK1 inhibitors for the treatment of rheumatoid arthritis. Bioorg. Med. Chem. Lett. 2022, 74, 128905. https://doi.org/10.1016/j.bmcl.2022.128905.
298. Cascioferro, S.; Parrino, B.; Spanò, V.; Carbone, A.; Montalbano, A.; Barraja, P.; Diana, P.; Cirrincione, G. An overview on the recent developments of 1,2,4-triazine derivatives as anticancer compounds. Eur. J. Med. Chem. 2017, 142, 328–375. https://doi.org/10.1016/j.ejmech.2017.08.009.
299. Asati, V.; Anant, A.; Patel, P.; Kaur, K.; Gupta, G.D. Pyrazolopyrimidines as anticancer agents: A review on structural and target-based approaches. Eur. J. Med. Chem. 2021, 225, 113781. https://doi.org/10.1016/j.ejmech.2021.113781.
300. Soltan, O.M.; Shoman, M.E.; Abdel-Aziz, S.A.; Narumi, A.; Konno, H.; Abdel-Aziz, M. Molecular hybrids: A five-year survey on structures of multiple targeted hybrids of protein kinase inhibitors for cancer therapy. Eur. J. Med. Chem. 2021, 225, 113768. https://doi.org/10.1016/j.ejmech.2021.113768.
301. Yamagishi, H.; Inoue, T.; Nakajima, Y.; Maeda, J.; Tominaga, H.; Usuda, H.; Hondo, T.; Moritomo, A.; Nakamori, F.; Ito, M.; et al. Discovery of tricyclic dipyrrolopyridine derivatives as novel JAK inhibitors. Bioorg. Med. Chem. 2017, 25, 5311–5326. https://doi.org/10.1016/j.bmc.2017.07.043.
302. Kiss, R.; Polgár, T.; Kirabo, A.; Sayyah, J.; Figueroa, N.C.; List, A.F.; Sokol, L.; Zuckerman, K.S.; Gali, M.; Bisht, K.S.; et al. Identification of a novel inhibitor of JAK2 tyrosine kinase by structure-based virtual screening. Bioorg. Med. Chem. Lett. 2009, 19, 3598–3601. https://doi.org/10.1016/j.bmcl.2009.04.138.
303. Wilson, L.J.; Malaviya, R.; Yang, C.; Argentieri, R.; Wang, B.; Chen, X.; Murray, W.V.; Cavender, D. Synthetic staurosporines via a ring closing metathesis strategy as potent JAK3 inhibitors and modulators of allergic responses. Bioorg. Med. Chem. Lett. 2009, 19, 3333–3338. https://doi.org/10.1016/j.bmcl.2009.04.039.

304. Ioannidis, S.; Lamb, M.L.; Davies, A.M.; Almeida, L.; Su, M.; Bebernitz, G.; Ye, M.; Bell, K.; Alimzhanov, M.; Zinda, M. Discovery of pyrazol-3-ylamino pyrazines as novel JAK2 inhibitors. Bioorg. Med. Chem. Lett. 2009, 19, 6524–6528. https://doi.org/10.1016/j.bmcl.2009.10.054.
305. Ledeboer, M.W.; Pierce, A.C.; Duffy, J.P.; Gao, H.; Messersmith, D.; Salituro, F.G.; Nanthakumar, S.; Come, J.; Zuccola, H.J.; Swenson, L.; et al. 2-Aminopyrazolo[1,5-a]pyrimidines as potent and selective inhibitors of JAK2. Bioorg. Med. Chem. Lett. 2009, 19, 6529–6533. https://doi.org/10.1016/j.bmcl.2009.10.053.
306. Wang, T.; Ledeboer, M.W.; Duffy, J.P.; Salituro, F.G.; Pierce, A.C.; Zuccola, H.J.; Block, E.; Shlyakter, D.; Hogan, J.K.; Bennani, Y.L. A novel chemotype of kinase inhibitors: Discovery of 3,4-ring fused 7-azaindoles and deazapurines as potent JAK2 inhibitors. Bioorg. Med. Chem. Lett. 2010, 20, 153–156. https://doi.org/10.1016/j.bmcl.2009.11.021.
307. Wei, H.; Myers, M. R.; Hanney, B.; Spada, A. P.; Bilder, G.; Galzcinski, H.; Amin, D.; Needle, S.; Page, K.; Jayyosi, Z.; Perrone, M. H. Potent Quinoxaline-Based Inhibitors of PDGF Receptor Tyrosine Kinase Activity. Part 2: The Synthesis and Biological Activities of RPR127963, an Orally Bioavailable Inhibitor. Bioorg. Med. Chem. Lett. 2003, 13, 3097–3100. https://doi.org/10.1016/S0960-894X(03)00655-3.
308. Serafim, R.A.M.; Sorrell, F.J.; Berger, B.T.; Collins, R.J.; Vasconcelos, S.N.S.; Massirer, K.B.; Knapp, S.; Bennett, J.; Fedorov, O.; Patel, H.; et al. Discovery of a Potent Dual SLK/STK10 Inhibitor Based on a Maleimide Scaffold. J. Med. Chem. 2021, 64, 13259–13278. https://doi.org/10.1021/acs.jmedchem.0c01579.
309. Ruel, R.; Thibeault, C.; L'Heureux, A.; Martel, A.; Cai, Z.W.; Wei, D.; Qian, L.; Barrish, J.C.; Mathur, A.; D'Arienzo, C.; et al. Discovery and preclinical studies of 5-isopropyl-6-(5-methyl-1,3,4-oxadiazol-2-yl)-N-(2-methyl-1H-pyrrolo[2,3-b]pyridin-5-yl)pyrrolo[2,1-f][1,2,4]triazin-4-amine (BMS-645737), an in vivo active potent VEGFR-2 inhibitor. Bioorg. Med. Chem. Lett. 2008, 18, 2985–2989. https://doi.org/10.1016/j.bmcl.2008.03.057.
310. Das, J.; Moquin, R.V.; Pitt, S.; Zhang, R.; Shen, D.R.; McIntyre, K.W.; Gillooly, K.; Doweyko, A.M.; Sack, J.S.; Zhang, H.; et al. Pyrazolo-pyrimidines: A novel heterocyclic scaffold for potent and selective p38 alpha inhibitors. Bioorg. Med. Chem. Lett. 2008, 18, 2652–2657. https://doi.org/10.1016/j.bmcl.2008.03.019.
311. Bhide, R.S.; Cai, Z.W.; Zhang, Y.Z.; Qian, L.; Wei, D.; Barbosa, S.; Lombardo, L.J.; Borzilleri, R.M.; Zheng, X.; Wu, L.I.; et al. Discovery and preclinical studies of (R)-1-(4-(4-fluoro-2-methyl-1H-indol-5-yloxy)-5- methylpyrrolo[2,1-f][1,2,4]triazin-6-yloxy)propan- 2-ol (BMS-540215), an in vivo active potent VEGFR-2 inhibitor. J. Med. Chem. 2006, 49, 2143–2146. https://doi.org/10.1021/jm051106d.
312. Borzilleri, R.M.; Bhide, R.S.; Barrish, J.C.; D'Arienzo, C.J.; Derbin, G.M.; Fargnoli, J.; Hunt, J.T.; Jeyaseelan, R.; Kamath, A.; Kukral, D.W.; et al. Discovery and evaluation of N-cyclopropyl- 2,4-difluoro-5-((2-(pyridin-2-ylamino)thiazol-5- ylmethyl)amino)benzamide (BMS-605541), a selective and orally efficacious inhibitor of vascular endothelial growth factor receptor-2. J. Med. Chem. 2006, 49, 3766–3769. https://doi.org/10.1021/jm060347y.
313. Liu, C.; Wrobleski, S.T.; Lin, J.; Ahmed, G.; Metzger, A.; Wityak, J.; Gillooly, K.M.; Shuster, D.J.; McIntyre, K.W.; Pitt, S.; et al. 5-Cyanopyrimidine derivatives as a novel class of potent, selective, and orally active inhibitors of p38alpha MAP kinase. J. Med. Chem. 2005, 48, 6261–6270. https://doi.org/10.1021/jm0503594.
314. Sugimoto, Y.; Sawant, D.B.; Fisk, H.A.; Mao, L.; Li, C.; Chettiar, S.; Li, P.K.; Darby, M.V.; Brueggemeier, R.W. Novel pyrrolopyrimidines as Mps1/TTK kinase inhibitors for breast cancer. Bioorg. Med. Chem. 2017, 25, 2156–2166. https://doi.org/10.1016/j.bmc.2017.02.030.
315. Poli, G.; Seidel, T.; Langer, T. Conformational Sampling of Small Molecules With iCon: Performance Assessment in Comparison With OMEGA. Front. Chem. 2018, 6, 229. https://doi.org/10.3389/fchem.2018.00229.
316. Wolber, G.; Langer, T. LigandScout: 3-D pharmacophores derived from protein-bound ligands and their use as virtual screening filters. J. Chem. Inf. Model. 2005, 45, 160–169. https://doi.org/10.1021/ci049885e.
317. Chrencik, J.E.; Patny, A.; Leung, I.K.; Korniski, B.; Emmons, T.L.; Hall, T.; Weinberg, R.A.; Gormley, J.A.; Williams, J.M.; Day, J.E.; et al. Structural and thermodynamic characterization of the TYK2 and JAK3 kinase domains in complex with CP-690550 and CMP-6. J. Mol. Biol. 2010, 400, 413–433. https://doi.org/10.1016/j.jmb.2010.05.020.
318. Tsui, V.; Gibbons, P.; Ultsch, M.; Mortara, K.; Chang, C.; Blair, W.; Pulk, R.; Stanley, M.; Starovasnik, M.; Williams, D.; et al. A new regulatory switch in a JAK protein kinase. Proteins 2011, 79, 393–401. https://doi.org/10.1002/prot.22889.
